# The Effect of Dietary Supplements on Female Infertility in Terms of Endometrial Thickness, Pregnancy, Live Birth and Miscarriage: A Systematic Review and Meta-Analysis

**DOI:** 10.3390/nu18121942

**Published:** 2026-06-16

**Authors:** Mette Peters Michaelsen, Michelle Poulsen, Maria Borgstrøm, Helena Birk Wisby, Anne Ahrendt Bjerregaard, Ulrik Schiøler Kesmodel

**Affiliations:** 1 Department of Obstetrics and Gynecology, Aalborg University Hospital, 9000 Aalborg, Denmark; michelle.poulsen@rn.dk (M.P.); u.kesmodel@rn.dk (U.S.K.); 2 Department of Clinical Medicine, Aalborg University, 9000 Aalborg, Denmark; 3 Department of Obstetrics and Gynecology, Herlev and Gentofte University Hospital, 2730 Herlev, Denmark; maria.borgstroem@regionh.dk; 4 Department of Health Science and Technology, Aalborg University, 9220 Aalborg, Denmark; h.wisby@rn.dk; 5 Centre for Clinical Research and Prevention, Copenhagen University Hospital-Bispebjerg and Frederiksberg Hospital, 2000 Frederiksberg, Denmark; anne.ahrendt.bjerregaard@regionh.dk; 6 Department of Congenital Disorders, Statens Serum Institut, 2300 Copenhagen, Denmark

**Keywords:** female infertility, dietary supplements, endometrium, pregnancy rate, live birth, miscarriage

## Abstract

**Background/Objectives**: Although current research suggests beneficial effects of dietary supplements on female infertility, existing evidence is often inconsistent and of limited certainty. This systematic review and meta-analysis aimed to investigate the effect of dietary supplements on female infertility in terms of endometrial thickness, pregnancy, live birth, and miscarriage compared to placebo (primary objective) and compared to placebo or a no-treatment comparator (secondary objective). **Methods**: PubMed, Embase, and CENTRAL were searched up to March 2025. Randomized controlled trials assessing the effect of dietary supplements compared to placebo or a no-treatment comparator among infertile women were included. Screening, data extraction, risk of bias, and certainty of evidence assessments were conducted by two independent reviewers. Data was synthesized quantitatively using random effects restricted maximum likelihood models. **Results**: Twenty placebo-controlled and 20 no-treatment comparator studies were included. Most studies had some concerns in risk of bias. Primary analyses showed an improvement in endometrial thickness following N-acetyl-cysteine supplementation compared to placebo, while no effect was found for supplements on pregnancy-related outcomes. Certainty of evidence of primary analyses was low. Secondary analyses indicated positive differences in endometrial thickness and pregnancy-related outcomes following supplementation with different supplements compared to placebo and no treatment. **Conclusions**: This review found no high-certainty evidence that dietary supplements improve female infertility outcomes when compared with placebo. Secondary analyses combining placebo and no-treatment comparator studies generated hypotheses for myo-inositol, N-acetyl-cysteine, vitamin D, vitamin E, and ≥3 substance dietary supplements, but these are at higher risk of bias and require confirmation in adequately powered placebo-controlled trials with live birth as the primary outcome.

## 1. Introduction

Infertility is estimated to affect 17.5% of people at some point during their lives [1], and dietary supplements are commonly used by infertile women seeking to improve their reproductive outcomes [2,3,4]. Female factors contribute to infertility in 40–50% of couples [5]. In addition to causes like anovulation, tubal factors, and uterine conditions [6], 30% of infertile couples are diagnosed with idiopathic infertility [7]. While age, body mass index, and smoking have well-known documented impacts on female fertility [8], there is less certainty about the influence of nutrition. Dietary supplements are readily available and affordable [9], and are therefore a simple and quick way to consume various micronutrients instead of larger dietary changes.

The growing interest in dietary supplements in relation to female fertility is supported by the lower levels of micronutrients observed in infertile women, including vitamin B12, vitamin B6, vitamin D, selenium and zinc [10,11,12]. However, these observations are not consistently reflected in studies investigating the effect of taking these micronutrients as dietary supplements. For example, while multiple systematic reviews and meta-analyses on observational studies have shown that lower vitamin D levels can reduce the likelihood of biochemical and clinical pregnancy as well as live birth [13,14,15,16], there is uncertainty as to whether vitamin D supplementation can improve female fertility. One systematic review and meta-analysis, that included five randomized controlled trials (RCTs) studying vitamin D supplementation, showed improved chances of biochemical pregnancy, but no improvements for clinical pregnancy, ongoing pregnancy, or miscarriage in patients undergoing in vitro fertilization (IVF) [17]. Another systematic review and meta-analysis that included nine RCTs and three cohort studies found an improved clinical pregnancy rate following vitamin D supplementation, but not an improved biochemical pregnancy rate [18]. Several other dietary supplements have also been investigated in relation to their potential impact on female fertility. Vitamin E has been shown to improve endometrial thickness (EMT) in women with idiopathic infertility who are undergoing controlled ovarian stimulation and intrauterine insemination (IUI) [19], and coenzyme Q10 has been associated with an increased clinical pregnancy rate in women undergoing assisted reproductive technology (ART) treatment [20]. Additionally, increased clinical pregnancy rates and live birth rates have been reported in women taking vitamin B complex compared to those taking only folic acid supplements prior to ART treatment [21]. However, the current literature still lacks a complete understanding of the effect of dietary supplements on female infertility.

Existing systematic reviews and meta-analyses on this topic have important limitations, including mixing observational and experimental study designs in their syntheses [18,22] and including heterogeneous study populations with both women with or without fertility impairment or with male-factor infertility involvement [22,23]. Additionally, the small number of RCTs investigating the same dietary supplements has been highlighted [2]. Although placebo and blinding should be of lower concern for objective outcomes, participants’ awareness of the treatment allocation could potentially influence their behavior. Therefore, despite the availability of current research, conclusions regarding the effects of dietary supplements on female infertility are hampered by limitations in the existing evidence.

Therefore, the primary aim was to investigate the effect of dietary supplements on female infertility in terms of EMT and rates of pregnancy, live birth, and miscarriage compared to placebo through a systematic review and meta-analysis. As a secondary aim, it was assessed whether inclusion of studies using either placebo or no-treatment comparator (NTC) had an impact on the same outcomes.

## 2. Materials and Methods

This study was reported according to the Preferred Reporting Items for Systematic Reviews and Meta-Analyses (PRISMA) 2020 statement [24]. A completed PRISMA checklist is provided in Table S1. The study has been prospectively registered in PROSPERO (CRD420251000655), with the full protocol uploaded to the registry.

### 2.1. Eligibility Criteria

RCTs fulfilling the inclusion criteria were included (Table 1). Medications commonly used in medically assisted reproduction (MAR), including Follicle Stimulating Hormone, human Menopausal Gonadotropins, Gonadotropin-Releasing Hormone agonists and antagonists, human Chorionic Gonadotropin (hCG), estradiol, clomiphene citrate (CC), letrozole, progesterone for luteal phase support, and contraceptives to induce bleeding were acceptable as co-treatment if administered to all relevant study arms. Further, metformin administered to women with Polycystic Ovary Syndrome (PCOS) and folic acid were acceptable as co-treatment if used in all relevant study arms. The outcomes live birth and clinical pregnancy were chosen as they represent the most clinically and critically relevant outcomes regarding reproductive success. Biochemical and undefined pregnancy were included to evaluate early reproductive outcomes and allow the inclusion of all relevant pregnancy data, even when the determination method was not specified, respectively. Miscarriage was selected to assess potential adverse effects, and EMT was chosen as this measure is important for blastocyst implantation and is exclusively related to the female. Placebo was defined as an inert comparator treatment or a dietary supplement other than the intervention if it was administered to all relevant study arms under blinded conditions. Animal and in vitro studies, duplicates, unavailable full texts, abstract-only papers, retracted articles, articles with an expression of concern, and articles not written in the Latin alphabet were excluded.

### 2.2. Information Sources and Selection Process

With support from a librarian, PubMed, Embase, and the Cochrane Central Register of Controlled Trials (CENTRAL) were searched on 7 March 2025 with no restrictions on geographical origin or publication year. Complete search strategies are provided in Table S2A–D [26]. All records, including trial registrations and abstracts, were evaluated. Further, references of included articles were hand-searched. After duplicate removal in EndNote^TM^ v.20.1, Clarivate, screening was conducted in Rayyan©, 2025, Rayyan Systems, Inc. (Cambridge, MA, USA) by two independent reviewers under blinded conditions [27].

### 2.3. Data Collection

Data from eligible RCTs were extracted by two independent reviewers using a standardized extraction form: author, year, country, study design, blinding, infertility diagnosis, age of participants, sample size, intervention, comparator, fertility treatment and medications received, duration of treatment, outcome measures, effect estimates, and conclusion. In cross-over studies, only data from the first treatment phase was extracted. When outcomes were reported from multiple cycles, EMT estimates were extracted from the first cycle to maximize sample size, while pregnancy-related effect estimates were extracted from the last cycle to allow maximum time to experience the outcome. EMT effect estimates aggregated from multiple cycles were not extracted. Subgroup effect estimates were not extracted. Treatment duration was extracted from supplement initiation until time of confirmed biochemical pregnancy to standardize durations across studies as some continued supplementation beyond pregnancy confirmation. Further, in cases where multiple treatment cycles were possible, the minimum treatment duration was extracted to ensure that it reflected an exposure time applicable to the entire study population. For studies not reporting the exact treatment duration, this was estimated based on the average regular menstrual cycle length and standard timeline for MAR cycles [28,29]. Corresponding authors were contacted if information was missing.

### 2.4. Study Risk of Bias Assessment

Risk of bias was assessed using the revised Cochrane Risk of Bias tool for randomized trials (RoB 2) [30]. Assessments were grouped into (1) EMT and (2) pregnancy-related outcomes, i.e., biochemical pregnancy, clinical pregnancy, undefined pregnancy, live birth, and miscarriage. Initial reviewer calibration ensured consistency in the assessment method. Two independent reviewers performed the assessments, and disagreements were resolved through discussion or a third party. The robvis© 2025 tool was used to visualize the assessments [31].

### 2.5. Synthesis Methods

Studies were grouped according to the administered dietary supplements. Supplements containing ≥3 substances were grouped as multiple substance dietary supplements.

Quantitative synthesis was performed when ≥2 studies were available. Studies with ≥2 relevant study arms were pooled to minimize unit of analysis issues. Meta-analyses were conducted using random effects restricted maximum likelihood models. Random effects models were chosen to account for differences between the included studies, such as geographical setting and infertility diagnosis. EMT was analyzed using mean differences with 95% confidence intervals (95% CIs). Dichotomous outcomes (i.e., pregnancy-related outcomes) were analyzed using risk ratios with 95% CIs. Primary analyses were conducted on placebo-controlled studies. In the secondary analyses, studies using either placebo or NTC were both included. While placebo-controlled and NTC studies systematically differ, particularly due to differences in blinding and potential differences in participant behavior in NTC studies, their inclusion in the secondary analyses was considered relevant to explore the consistency of results across study designs and provide a broader overview of the available evidence. Subgroup analyses using placebo-controlled studies included analyses divided into (1) whether the supplement contained one or two substances, (2) MAR treatment received grouped as IVF/intracytoplasmic sperm injection (ICSI), IUI/ovulation induction, and no treatment, and (3) duration of treatment. Further, exploratory subgroup analyses based on infertility diagnoses independent of the exposure were performed. Planned sensitivity analyses excluding high risk of bias studies could not be conducted as all studies included in the primary analyses had some concerns or low risk of bias. Further, as some studies reported single or double-arm zero events, post hoc sensitivity analyses, excluding zero-event studies, were conducted where possible to assess their influence on the results.

To evaluate heterogeneity, *I*^2^ estimates were interpreted using the Cochrane threshold recommendations [32], and Chi^2^ with a chosen significance level of 0.05 were used: *I*^2^ = 0–40% was considered low heterogeneity, *I*^2^ = 30–60% moderate heterogeneity, *I*^2^ = 50–90% substantial heterogeneity, and *I*^2^ = 75–100% considerable heterogeneity. For the overlapping thresholds, the *p*-value of the Chi^2^ *p*-value was used to determine the level of heterogeneity; for example, if an *I*^2^ = 35% and *p* < 0.05 heterogeneity was considered to be moderate. Publication bias assessments using funnel plots were not conducted as ≤ 10 studies were available in all analyses [33]. Analyses were conducted using the Stata 18 software©, StataCorp LLC (College Station, TX, USA) [34].

### 2.6. Certainty Assessment

Certainty of evidence was evaluated for primary analyses based on the Grading of Recommendations Assessment, Development, and Evaluation (GRADE) approach using the GradePRO GDT, 2023, McMaster University and Evidence Prime software [35,36].

## 3. Results

### 3.1. Study Selection

**Figure 1:**
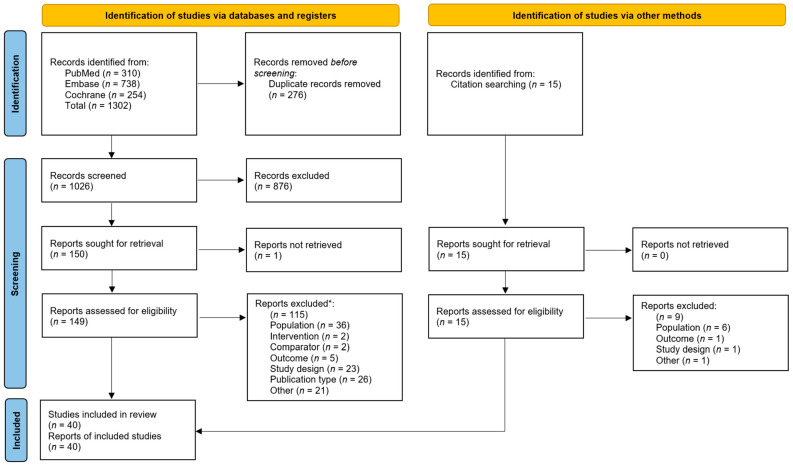
PRISMA flow diagram. * Some papers might have more than one reason for exclusion; however, the main reason was used for categorization.

Of the 1302 records identified, 40 studies met the inclusion criteria, of which 20 were placebo-controlled and 20 used NTC (Figure 1). Seventeen placebo-controlled and 17 NTC studies were eligible for quantitative synthesis. Exclusion reasons based on full text screening and corresponding author contacts can be seen in Tables S3 and S4.

### 3.2. Study Characteristics

Participants (*n* = 3589) were 15–44 years, with the most common condition of infertility being PCOS. Seventeen studies were conducted in Iran, four in Egypt, four in Italy, four in Turkey, and two in India. Further, the United Kingdom, Spain, Poland, Japan, Iraq, Israel, China, Nigeria, and Mexico were each represented by one study. The dietary supplements assessed were L-arginine [37,38], melatonin [39,40,41,42], Myo-inositol [43,44,45,46,47,48,49], N-acetyl-cysteine [50,51,52,53,54,55], vitamin C [56,57], vitamin D [49,58,59,60,61,62], vitamin E [19,56,58,63], multiple substance dietary supplements [64,65,66,67,68], and other dietary supplements [52,69,70,71,72,73,74,75]. Of the 20 placebo-controlled studies, five used folic acid as placebo, and no studies administered additional folic acid to all study arms. Of the 20 NTC studies, five administered folic acid to all study arms. All placebo-controlled studies had a parallel design, with two being single-blinded, 12 being double-blinded, five being triple-blinded, and one having unclear blinding. Among the NTC studies, 19 had a parallel design and one was factorial (Table S5).

### 3.3. Risk of Bias Evaluations

**Figure 2:**
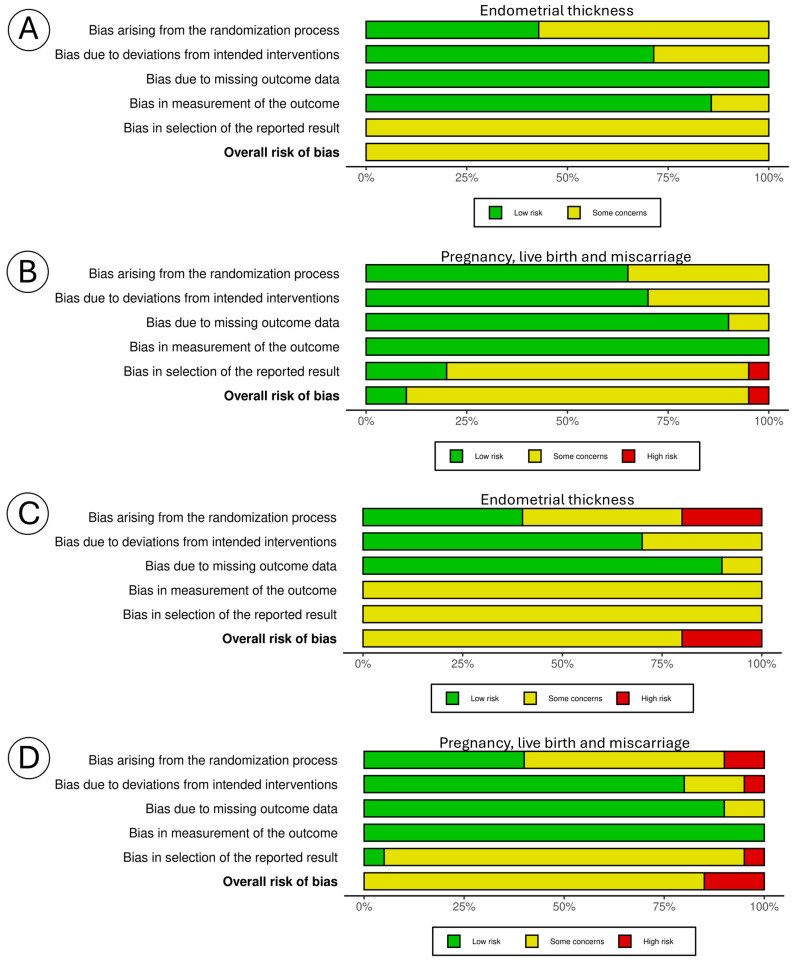
Summary plots of risk of bias assessments on (**A**) endometrial thickness in placebo-controlled studies; (**B**) pregnancy, live birth, and miscarriage outcomes in placebo-controlled studies; (**C**) endometrial thickness in studies using no treatment as the comparator; and (**D**) pregnancy, live birth, and miscarriage outcomes in studies using no treatment as the comparator. The plots were created using the robvis© 2025 tool.

Most placebo-controlled studies had some concerns in overall risk of bias, primarily caused by bias in selection of the reported results due to lack of a publicly available protocol, retrospective registration, or not pre-specifying their outcomes (Figure 2, Figures S1 and S2). In NTC studies, overall risk of bias was of some concern or high. Reasons for downgrading often included bias in selection of the reported results and bias related to the randomization process. Further, bias in the measurement of the outcome was often a reason for downgrading for EMT, as the outcome assessor’s knowledge of the treatment received could potentially have influenced their assessments (Figure 2, Figures S3 and S4).

### 3.4. Qualitative Synthesis

#### 3.4.1. L-arginine

One placebo-controlled study evaluating the efficacy of 16 g/day L-arginine administered for 8 weeks found a difference in clinical pregnancy rate favoring placebo and no difference in EMT [37]. Similarly, one NTC study found no difference in EMT using the same dosage and treatment duration. Additionally, three clinical pregnancies occurred in the intervention arm, but all resulted in miscarriage [38].

#### 3.4.2. Melatonin

One placebo-controlled study evaluating the efficacy of 3 mg/day melatonin [39] for 8 weeks found no difference in pregnancy-related outcomes [39]. Similarly, three NTC studies administering 3–6 mg/day melatonin for 6–8 weeks found no difference in either EMT [40] or pregnancy-related outcomes following supplementation [40,41,42].

#### 3.4.3. Myo-Inositol

In three placebo-controlled studies evaluating the efficacy of 4 g/day myo-inositol [43,44,45] for 2–3 months, most found no difference in pregnancy-related outcomes [43,44]. In contrast, of the four NTC studies evaluating the efficacy of 2–4 g/day myo-inositol administered alone [46,48], with 200 µg folic acid [47], or 1000 IU/day vitamin D [49] for 4–16 weeks, three studies reported improvements in pregnancy-related outcomes [46,48,49].

#### 3.4.4. N-acetyl-cysteine

In two placebo-controlled studies evaluating the efficacy of 1.2 g/day N-acetyl-cysteine administered for 5 days, from the third to the seventh cycle day [50,51], EMT improved in one study [50] and undefined pregnancy improved in both [50,51]. Four NTC studies evaluated 1.2–1.8 g/day N-acetyl-cysteine administered for 5 days–4 weeks [52,53,54,55]. All studies assessed EMT and only one found improvements [52], one reported improvements in pregnancy rate [55], and no difference was seen in miscarriage rate [54,55].

#### 3.4.5. Vitamin C

One placebo-controlled study evaluating the efficacy of 343 mg/day vitamin C with 84 mg/day vitamin E for 6 months found no difference in undefined pregnancy rates [56]. In contrast, one NTC study found an improvement in biochemical pregnancy rate after 750 mg/day vitamin C supplementation for 4 weeks [57].

#### 3.4.6. Vitamin D

In two placebo-controlled studies evaluating the efficacy of 50,000 IU/week vitamin D administered alone for 6 weeks [59] or 50,000 IU/biweekly vitamin D with 400 mg/day vitamin E for 8 weeks [58], improvements were observed in all pregnancy-related outcomes [58,59] while no difference was seen in EMT [58]. Similarly, of four NTC studies administering 50,000 IU/week vitamin D alone [60,62], 400 IU/day vitamin D with 1000 mg/day calcium [61], or 1000 IU/day vitamin D with 2 g/day myo-inositol [49] for 1–3 months, three studies reported improvements in pregnancy-related outcomes [49,60,62], and one reported no difference in EMT or miscarriage rate [49].

#### 3.4.7. Vitamin E

Two placebo-controlled studies evaluated the efficacy of 84 mg/day vitamin E with 343 mg/day vitamin C for 6 months [56] or 400 mg/day vitamin E with 50,000 IU/biweekly vitamin D for 8 weeks [58]. While supplementation with vitamin E and vitamin C did not result in a difference in pregnancy rate [56], vitamin E and vitamin D supplementation was found to improve all measured pregnancy-related outcomes but EMT [58]. In two NTC studies, 400–1500 IU/day vitamin E administered for 2–8 weeks improved EMT, and no differences were found in pregnancy rates [19,63].

#### 3.4.8. Multiple Substance Dietary Supplements

In three placebo-controlled studies evaluating the efficacy of different multiple substance dietary supplements [64,65,66] for 4–15 weeks, improvements were seen in EMT [65], clinical pregnancy [64,65,66], and live birth rates [66] following supplementation. No difference was seen in miscarriage rate [64,66]. Two NTC studies administering multiple substance dietary supplements for 10 weeks found no difference in any of the outcome measures [67,68].

#### 3.4.9. Other Dietary Supplements

The efficacy of other dietary supplements was evaluated across seven placebo-controlled studies. Overall, 300 mg/day Vitamin B1 for 4 weeks [70], LactoFem^®^ for 6 months [73], EQOQ^®^ for 17 weeks [74], and 3 g/day L-carnitine for 4 weeks [75] improved all reported pregnancy-related outcomes [70,73,74,75] and EMT [75], while 1800 mg/day omega-3 fatty acids for 4 weeks [69], 200 µ/day selenium for 8 weeks [71], and 6 mg/day astaxanthin for 12 weeks [72] did not improve either [69,71,72]. One NTC study reported improvements in EMT and clinical pregnancy rate following 400 µg/day chromium picolinate supplementation for 4 weeks [52].

### 3.5. Quantitative Synthesis

#### 3.5.1. L-arginine

Secondary analyses showed no difference in EMT and clinical pregnancy rate following L-arginine supplementation. Heterogeneity was considerable and substantial, respectively (Table 2, Figure S5).

#### 3.5.2. Melatonin

Secondary analyses showed no difference in biochemical pregnancy, clinical pregnancy, and miscarriage rates with low heterogeneity (Table 2, Figure S6).

#### 3.5.3. Myo-Inositol

Primary analyses showed no difference in clinical pregnancy and miscarriage rates following myo-inositol supplementation with substantial and low heterogeneity, respectively (Table 2, Figure S7). Excluding zero-event studies from analysis on clinical pregnancy did not alter the direction of the results (Figure S8). Secondary analyses suggested a positive difference in biochemical pregnancy rate with low heterogeneity, and no difference in clinical pregnancy, live birth, and miscarriage rates with low heterogeneity (Table 2, Figure S9). Subgroup analyses showed no difference in clinical pregnancy or miscarriage rates when myo-inositol was administered alone, with substantial and low heterogeneity, respectively (Figure S10). Subgroup analyses showed no difference in clinical pregnancy rate following >10 weeks of treatment with low heterogeneity (Figure S11). Subgroup analyses suggested a positive difference in clinical pregnancy rate and no difference in miscarriage rates in IVF/ICSI cycles with low heterogeneity (Figure S12).

#### 3.5.4. N-acetyl-cysteine

Primary analyses showed an improvement in EMT with low heterogeneity and no difference in undefined pregnancy rate following N-acetyl-cysteine supplementation with substantial heterogeneity (Table 2, Figure S13). Secondary analyses suggested a positive difference in clinical pregnancy rate with low heterogeneity and no difference in EMT, undefined pregnancy, or miscarriage rates with considerable, substantial, and low heterogeneity, respectively (Table 2, Figure S14).

#### 3.5.5. Vitamin D

Secondary analyses suggested a positive difference in biochemical pregnancy and live birth rates with low heterogeneity and no difference in undefined pregnancy rate following vitamin D supplementation with substantial heterogeneity (Table 2, Figure S15).

#### 3.5.6. Vitamin E

Secondary analyses suggested a positive difference in EMT following vitamin E supplementation with substantial heterogeneity (Table 2, Figure S16).

#### 3.5.7. Multiple Substance Dietary Supplements

Primary analyses showed no difference in clinical pregnancy or miscarriage rates following supplementation with multiple substance dietary supplements with substantial heterogeneity (Table 2, Figure S17). Secondary analyses suggested a positive difference in clinical pregnancy rate with substantial heterogeneity and no difference in miscarriage rate with moderate heterogeneity (Table 2, Figure S18). Subgroup analyses showed no difference in clinical pregnancy or miscarriage rates in ovulation induction cycles with considerable and substantial heterogeneity, respectively (Figure S19).

#### 3.5.8. Exploratory Subgroup Analysis

Among the placebo-controlled studies, only those involving individuals with PCOS were suitable for explorative subgroup analyses. Additionally, PCOS was further defined by whether it was complicated by CC resistance. Analyses including infertile women with PCOS suggested a positive difference in EMT (substantial heterogeneity), biochemical pregnancy (moderate heterogeneity), clinical pregnancy (considerable heterogeneity), undefined pregnancy (low heterogeneity), and live birth rates (low heterogeneity), while no difference was seen in miscarriage rate (substantial heterogeneity) (Figure S20). Analyses including infertile women with PCOS and CC resistance suggested a positive difference in EMT with considerable heterogeneity (Figure S21).

### 3.6. Certainty of Evidence

All primary analyses were evaluated as having low certainty of evidence. Reasons for downgrading were generally due to risk of bias in the included studies and imprecision attributable to small sample sizes (Tables S6–S8).

## 4. Discussion

In this systematic review and meta-analysis, N-acetyl-cysteine supplementation had a positive effect on EMT, while no effect was seen of dietary supplements on pregnancy-related outcomes compared to placebo. However, the low certainty of evidence in all primary analyses, the small number of available studies within each analysis, and the presence of risk of bias in almost all included studies underline that no solid conclusions can be drawn. In the secondary analyses considering both placebo-controlled and NTC studies, indications of positive effects were seen for several dietary supplements on EMT and pregnancy-related outcomes. However, these findings should be interpreted cautiously, as the analyses were based on studies with higher risk of bias and an overall lower certainty.

Many previous systematic reviews and meta-analyses on the effect of dietary supplements on female infertility did not exclusively include placebo-controlled studies in their quantitative syntheses. Similar to the current results, two systematic reviews and meta-analyses assessing the effect of melatonin compared to placebo, NTC, or another adjuvant during ART found no effect on biochemical pregnancy, clinical pregnancy, or miscarriage rate [76,77]. One systematic review and meta-analysis found no effect on clinical pregnancy following myo-inositol supplementation compared to placebo or standard care during ART [78] in accordance with our findings of the secondary analysis, while another showed an improvement in clinical pregnancy rate following myo-inositol and folic acid supplementation compared to folic acid administered alone [23]. This latter systematic review and meta-analysis included both RCTs and a cohort study in their analysis, and some of the included studies were excluded in the current systematic review and meta-analysis due to the presence of male-factor infertility. Additionally, they found a decrease in miscarriage rates; however, their analyses were based on both miscarriage per woman and miscarriage per pregnancy estimates [23], whereas the current systematic review and meta-analysis solely extracted miscarriages per woman. Two systematic reviews and meta-analyses investigating the effect of vitamin D compared to placebo, no treatment, and no additional treatment other than the same co-treatment as the intervention group on women with PCOS [79], or compared to placebo, no treatment, and other dietary supplements on infertile women undergoing ART [18] detected an improvement in clinical pregnancy rate in alignment with the secondary analyses of the current study. Thus, most similarities with other meta-analyses were found when comparing with the secondary analyses. In the majority of the secondary analyses, the direction of the effect was consistent with primary analyses, and multiple analyses suggested an improving effect of different dietary supplements on EMT and pregnancy-related outcomes. However, caution is warranted when interpreting findings from the secondary analyses as these should be seen as hypothesis-generating due to their higher risk of bias and limited certainty. In NTC studies, potential effects may be affected because women in the control group may acquire the dietary supplements themselves or change behavior. While only a few of the NTC studies considered this by excluding participants using dietary supplements prior to study initiation, none assessed whether participants in the control groups changed their behavior during the study period. Additionally, the outcome EMT may be vulnerable to intra- and interobserver variation in measurement as well as detection bias, which is supported by the risk of bias assessments, where all NTC studies evaluating EMT had issues related to the measurement of the outcome. Thus, including NTC studies may have increased statistical power due to the increased number of studies, but concurrently reduced the methodological quality, as these studies generally had higher risk of bias. While the findings and methodological approaches vary, there is consensus across most systematic reviews that the current evidence is limited, of low quality, and does not allow a definitive answer as to whether dietary supplements have an impact on female infertility [2,76,77,80].

Among the studies included in this systematic review and meta-analysis, a relatively large proportion involved infertile women diagnosed with PCOS. PCOS is a common reproductive disorder involving anovulation in up to 75% of cases [81], whereas ovulation disorders only account for approximately 25% of infertility cases overall [82]. This may affect the generalizability of findings, as it is possible that the dietary supplements may have different effects in women with other causes of female infertility. In the exploratory subgroup analyses conducted to investigate the effect on different infertility diagnoses irrespective of the dietary supplements used, results suggested that infertile women with PCOS may benefit from supplementation. However, it should be considered that these analyses pooled different interventions. Therefore, no specific dietary supplements can be singled out to have an effect. Although the analyses were exploratory, they highlight the need for further research focusing on specific dietary supplements for women with PCOS.

In this current systematic review and meta-analysis, two methodological considerations regarding the co-administration of dietary supplements other than the intervention were applied: folic acid was accepted as a co-treatment when administered to all relevant study arms, and dietary supplements other than the intervention were accepted as placebo if administered under blinded conditions to all study arms. These approaches were chosen to reflect how dietary supplements are used and recommended in reproductive medicine. Folic acid is not pharmacologically inert and may therefore influence reproductive outcomes. However, as this was only accepted as a co-treatment or placebo if administered to all study arms, it represents an equal baseline exposure rather than an active comparator. Consequently, the interpretation of these studies relates to the additional effect of the intervention on top of folic acid, which in many cases reflects clinical practice. The use of dietary supplements is common among women attempting to conceive and infertile women [83,84,85]. Further, folic acid is recommended by the World Health Organization (WHO) for women attempting to conceive to prevent neural tube defects [86]. Therefore, the chosen methodological approach is not expected to affect the generalizability of the findings considerably, although the potential attenuation of effect estimates should be considered when interpreting the results. Notably, only a limited number of the included studies reported administration of folic acid to all study arms despite the international recommendations from the WHO. Future RCTs should consider administering folic acid to all participants regardless of study design rather than possibly relying on participants to take folic acid themselves.

Several micronutrients have been suggested to support female fertility through common mechanisms, including reduction in oxidative stress through antioxidative properties, hormonal and metabolic regulation, as well as supporting processes related to oocyte maturation, quality, and implantation [87]. Despite suggested beneficial properties and the biological role of the assessed substances, no effect was found for most clinical outcomes. Antioxidant and insulin-sensitizing properties of N-acetyl-cysteine have been suggested to have a positive impact on PCOS, a condition associated with oxidative stress and insulin resistance [88,89]. The primary meta-analysis revealed an improvement in EMT and included studies with infertile women with PCOS using similar dosages and treatment durations of N-acetyl-cysteine. However, as the analysis was based on only two small studies, the findings require confirmation from larger trials. The insulin-sensitizing properties of myo-inositol have likewise been proposed for PCOS. Further, myo-inositol is involved in many cellular processes, and its presence in follicular fluid has been suggested to improve oocyte and embryo quality [90]. However, the primary meta-analyses showed no improvement in pregnancy-related outcomes. While dosages and treatment durations were similar, variations in infertility diagnoses may have influenced the results. Melatonin and vitamin E have been hypothesized to reduce oxidative stress and thereby improve reproductive outcomes in infertile women [91,92]. Secondary meta-analyses showed no effect of melatonin but were limited by including few studies with different infertility diagnoses. Secondary analysis suggested a positive difference of vitamin E on EMT, but this result was also limited by study heterogeneity and a higher risk of bias. Although L-arginine is involved in the production of nitric oxide and thereby may improve uterine perfusion [87], secondary analyses suggested no difference in either EMT or pregnancy-related outcomes. Vitamin D affects sex hormone steroidogenesis and has therefore been suggested to improve reproductive outcomes [93]. While secondary analyses suggested positive differences in pregnancy-related outcomes, the analyses were limited due to pooling of different study designs, insufficient consideration of baseline vitamin D status, and differences related to the intervention. Overall, the current study was unable to confirm positive effects of dietary supplements on EMT and pregnancy-related outcomes. Due to limitations in the available literature, which are reflected in the meta-analyses, it cannot be ruled out that the observed null findings are due to methodological limitations rather than a true lack of effect. Nevertheless, the potential impact of dietary supplements on female infertility remains unclear. There is a need for more methodologically robust and adequately powered RCTs. Further, while observational studies are at a higher risk of bias, they may still contribute to hypotheses and provide valuable insights to inform future research. Beyond the scope of this systematic review and meta-analysis, other factors may influence the potential effects of dietary supplements on female fertility. These include nutrient status due to dietary patterns [94,95] and the bidirectional interaction between gut microbiome and dietary supplement absorption [96].

### Strengths and Limitations

This study was based on a number of methodological considerations which strengthen the review. The literature search included multiple sources to enhance the identification of relevant studies. The dual review method was applied to all elements of the process to minimize errors and bias. The population was confined to women with confirmed infertility with no involvement of male-factor infertility to ensure a targeted evaluation. Further, by conducting different types of analyses, including pre-defined primary, secondary, and subgroup analyses, different perspectives of the aim were assessed. Specifically, the involvement of both placebo-controlled and NTC studies contributes to a more comprehensive overview of the available evidence, while still acknowledging the limitations associated with this approach.

Limitations of this study should also be considered. Most of the included studies had some concerns regarding risk of bias, and certainty of evidence was low. Due to the limited data available from placebo-controlled trials, primary analyses could not be conducted for many of the dietary supplements. Further, meta-analyses were based on a low number of studies with small sample sizes, which likely reduced statistical power and precision, as well as reduced the generalizability of the results. The random effects model was chosen a priori to account for heterogeneity between studies. The low number of studies within each dietary supplement group reduced precision in the estimation of the between-study variance, thereby resulting in wider 95% CIs. Although a fixed effects model would have resulted in narrower 95% CIs, it would ignore the heterogeneity which was observed in many of the analyses. When heterogeneity is present, random effects models assign relatively more weight to smaller studies. However, this is unlikely to have had substantial impact on the results, as most included studies were small. Additionally, while extracting information on treatment duration until confirmed pregnancy was chosen to homogenize durations across studies, continued supplement use after confirmed pregnancy may influence some pregnancy-related outcomes. However, this may have limited impact on the results of the current study, as subgroup analyses on treatment duration could only be conducted for myo-inositol supplementation. Finally, the literature search was conducted on 7 March 2025, and studies published after this date are therefore not captured.

## 5. Conclusions

Based on primary analyses, the current study found no evidence of an effect of dietary supplements on female infertility. However, secondary analyses generated hypotheses regarding possible effects of vitamin E supplementation on EMT and of myo-inositol, N-acetyl-cysteine, vitamin D, and multiple substance dietary supplements on pregnancy-related outcomes. As the available studies have methodological limitations which introduce risk of bias and decrease overall certainty of evidence, future studies should aim for larger sample sizes and more methodological, rigorous approaches such as conducting blinded placebo-controlled studies and assessing clinically meaningful outcomes. Based on these results, specific advice on the use of dietary supplements in relation to female infertility cannot be provided.

## Figures and Tables

**Table 1:** Inclusion and exclusion criteria based on the PICO framework.

	Inclusion Criteria	Exclusion Criteria
Population	Women with confirmed infertility or female partners in couples with idiopathic infertility attempting to conceive spontaneously or through medically assisted reproduction *	Women with no diagnosed fertility issues, transgender women, infertile women with known uterine pathologies that have not been treated surgically (e.g., Asherman syndrome, intrauterine adhesions, septate uterus), women who had undergone transcervical resection of the endometrium and septum resection, and male infertility
Intervention	Dietary supplements	Diet, foods, medications
Comparison	Placebo or no treatment	-
Outcome	Endometrial thickness (bilayer measurement) Biochemical pregnancy (confirmed by serum β-hCG levels or urinary tests) Clinical pregnancy (confirmed by ultrasound) Undefined pregnancy (determination method not specified) Live birth (birth of a live child) Miscarriage (fetal death before 22 completed weeks of gestation)	-

* Infertility was defined as the inability to conceive after ≥1 year of regular unprotected intercourse [25].

**Table 2:** Results from primary and secondary analyses.

	Primary Analyses			Secondary Analyses		
	Studies	Effect Measure (95% CI) *	Heterogeneity Test	Studies	Effect Measure (95% CI) *	Heterogeneity Test
L-arginine						
Endometrial thickness	1	-	-	2	−1.05 (−2.81, 0.71)	*I*^2^ = 78.84% Chi^2^ *p* = 0.03
Biochemical pregnancy	0	-	-	0	-	-
Clinical pregnancy	1	-	-	2	1.36 (0.12, 15.64)	*I*^2^ = 61.59% Chi^2^ *p* = 0.11
Undefined pregnancy	0	-	-	0	-	-
Live birth	0	-	-	0	-	-
Miscarriage	0	-	-	1	-	-
Melatonin						
Endometrial thickness	0	-	-	1	-	-
Biochemical pregnancy	1	-	-	2	1.53 (0.79, 2.99)	*I*^2^ = 0.00 Chi^2^ *p* = 0.78
Clinical pregnancy	1	-	-	3	1.57 (0.83, 2.98)	I^2^ = 0.00% Chi^2^ *p* = 0.97
Undefined pregnancy	0	-	-	0	-	-
Live birth	0	-	-	0	-	-
Miscarriage	1	-	-	2	1.37 (0.31, 6.01)	*I*^2^ = 00.00% Chi^2^ *p* = 0.64
Myo-inositol						
Endometrial thickness	0	-	-	1	-	-
Biochemical pregnancy	1	-	-	2	1.90 (1.06, 3.41)	*I*^2^ = 0.00% Chi^2^ *p* = 0.93
Clinical pregnancy	3	1.58 (0.63, 3.95)	*I*^2^ = 62.89% Chi^2^ *p* = 0.07	6	1.55 (1.00, 2.40)	*I*^2^ = 31.77% Chi^2^ *p* = 0.26
Undefined pregnancy	0	-	-	1	-	-
Live birth	1	-	-	2	2.16 (0.87, 5.35)	*I*^2^ = 0.00% Chi^2^ *p* = 0.61
Miscarriage	2	1.66 (0.64, 4.30)	*I*^2^ = 00.00% Chi^2^ *p* = 0.68	4	1.34 (0.60, 2.98)	*I*^2^ = 00.00% Chi^2^ *p* = 0.78
N-acetyl-cysteine						
Endometrial thickness	2	1.09 (0.75, 1.43)	*I*^2^ = 0.00% Chi^2^ *p* = 0.56	4	0.60 (−0.01, 1.21)	*I*^2^ = 80.38% Chi^2^ *p* = 0.00
Biochemical pregnancy	0	-	-	1	-	-
Clinical pregnancy	0	-	-	2	1.36 (1.14, 1.63)	*I*^2^ = 0.00% Chi^2^ *p* = 0.49
Undefined pregnancy	2	6.03 (0.46, 78.35)	*I*^2^ = 70.05% Chi^2^ *p* = 0.07	2	6.03 (0.46, 78.35)	*I*^2^ = 70.05% Chi^2^ *p* = 0.07
Live birth	1	-	-	1	-	-
Miscarriage	1	--	--	3	1.94 (0.68, 5.56)	*I*^2^ = 0.00 Chi^2^ *p* = 0.76
Vitamin D						
Endometrial thickness	1	-	-	1	-	-
Biochemical pregnancy	1	-	-	3	2.38 (1.26, 4.50)	*I*^2^ = 22.03% Chi^2^ *p* = 0.39
Clinical pregnancy	1	-	-	1	-	-
Undefined pregnancy	0	-	-	2	1.83 (0.84, 3.98)	*I*^2^ = 62.11% Chi^2^ *p* = 0.10
Live birth	1	-	-	2	2.78 (1.61, 4.80)	*I*^2^ = 0.00% Chi^2^ *p* = 0.79
Miscarriage	0	-	-	0	-	-
Vitamin E						
Endometrial thickness	1	-	-	3	0.99 (0.13, 1.84)	*I*^2^ = 73.52% Chi^2^ *p* = 0.02
Biochemical pregnancy	0	-	-	1	-	-
Clinical pregnancy	0	-	-	1	-	-
Undefined pregnancy	1	-	-	1	-	-
Live birth	1	-	-	1	-	-
Miscarriage	0	-	-	0	-	-
Multiple substance dietary supplements						
Endometrial thickness	1	-	-	1	-	-
Biochemical pregnancy	0	-	-	1	-	-
Clinical pregnancy	3	2.63 (1.00, 6.92)	*I*^2^ = 80.49% Chi^2^ *p* = 0.05	5	1.70 (1.16, 2.50)	*I*^2^ = 51.25% Chi^2^ *p* = 0.03
Undefined pregnancy	0	-	-	0	-	-
Live birth	1	-	-	1	-	-
Miscarriage	2	1.26 (0.04, 44.70)	*I*^2^ = 74.64% Chi^2^ *p* = 0.05	3	1.26 (0.22, 7.29)	*I*^2^ = 53.45% Chi^2^ *p* = 0.12

* Endometrial thickness is reported as mean difference, while remaining outcomes are reported as risk ratios.

## Data Availability

No new data were created or analyzed in this study. Data sharing is not applicable to this article.

## Supplementary Materials

The following supporting information can be downloaded at: https://www.mdpi.com/article/10.3390/nu18121942/s1, Figure S1: Traffic-light plot of risk of bias assessments on the outcome endometrial thickness in placebo-controlled studies; Figure S2: Traffic-light plot of risk of bias assessments on pregnancy, live birth and miscarriage outcomes in placebo-controlled studies; Figure S3: Traffic-light plot of risk of bias assessments on the outcome endometrial thickness in studies using no comparator treatment; Figure S4: Traffic-light plot of risk of bias assessments on pregnancy, live birth and miscarriage outcomes in studies using no comparator treatment; Figures S5–S21: Forest plots; Table S1: PRISMA 2020 checklist [24]; Table S2A: Results from the systematic searches; Table S2B: Search strategy in PubMed [26]; Table S2C: Search strategy in Embase; Table S2D: Search strategy in the Cochrane Central Register of Controlled Trials; Table S3: Reasons for exclusion of identified articles based on full text screening; Table S4: Author contacts, responses and decisions regarding inclusion of studies and data extraction; Table S5: Characteristics of included studies; Table S6: Certainty of evidence assessment of myo-inositol compared to placebo for female infertility; Table S7: Certainty of evidence assessment of N-acetyl-cysteine compared to placebo for female infertility; Table S8. Certainty of evidence assessment of multiple substance dietary supplements compared to placebo for female infertility.

## Abbreviations

The following abbreviations are used in this manuscript:

ART	Assisted Reproductive Technology
CC	Clomiphene Citrate
EMT	Endometrial Thickness
GRADE	Grading of Recommendations Assessment, Development, and Evaluation
hCG	Human Chorionic Gonadotropin
ICSI	Intracytoplasmic Sperm Injection
IUI	Intrauterine Insemination
IVF	In Vitro Fertilization
MAR	Medically Assisted Reproduction
NTC	No-Treatment Comparator
PCOS	Polycystic Ovary Syndrome
PRISMA	Preferred Reporting Items for Systematic Reviews and Meta-Analyses
RCT	Randomized Controlled Trial
RoB2	Revised Cochrane Risk of Bias Tool for Randomized Trials
95% CI	95% Confidence Interval
