# Supplementary material for: The Effect of Dietary Supplements on Female Infertility in Terms of Endometrial Thickness, Pregnancy, Live Birth and Miscarriage: A Systematic Review and Meta-Analysis
Source: Nutrients. 2026 Jun 16;18(12):1942. doi: 10.3390/nu18121942 (PMC13306041; doi:10.3390/nu18121942)
Supplement: Supplementary file 1 [file nutrients-18-01942-s001.zip › nutrients-4338495-supplementary.pdf]

Supplementary tables and figures

The effect of dietary supplements on female infertility in terms of endometrial thickness, pregnancy, live birth and miscarriage: a systematic review and meta-analysis

Table of Contents

Table S1. PRISMA 2020 checklist. 2

Table S2A. Results from the systematic searches 6

Table S2B. Search strategy in PubMed 6

Table S2C. Search strategy in Embase 7

Table S2D. Search strategy in the Cochrane Central Register of Controlled Trials 8

Table S3. Reasons for exclusion of identified articles based on full text screening. 10

Table S4. Author contacts, responses and decisions regarding inclusion of studies and data extraction. 26

Table S5. Characteristics of included studies 28

Table S6. Certainty of evidence assessment of myo-inositol compared to placebo for female infertility. 40

Table S7. Certainty of evidence assessment of N-acetyl-cysteine compared to placebo for female infertility. 40

Table S8. Certainty of evidence assessment of multiple substance dietary supplements compared to placebo for female infertility. 40

Figure S1. 41

Figure S2. 42

Figure S3. 43

Figure S4. 44

Figure S5. 45

Figure S6. 46

Figure S7. 47

Figure S8. 47

Figure S9. 48

Figure S10. 49

Figure S11. 49

Figure S12. 50

Figure S13. 51

Figure S14. 52

Figure S15. 53

Figure S16. 53

Figure S17. 54

Figure S18. 55

Figure S19. 56

Figure S20. 57

Figure S21. 57

Table S1. PRISMA 2020 checklist.

Section and Topic	Item #	Checklist item	Location where item is reported
**TITLE**			
Title	1	Identify the report as a systematic review.	Page 1, lines 2-4
**ABSTRACT**			
Abstract	2	See the PRISMA 2020 for Abstracts checklist.	Page 1, lines 18-41
**INTRODUCTION**			
Rationale	3	Describe the rationale for the review in the context of existing knowledge.	Page 2, lines 45-87
Objectives	4	Provide an explicit statement of the objective(s) or question(s) the review addresses.	Page 2, lines 88-92
**METHODS**			
Eligibility criteria	5	Specify the inclusion and exclusion criteria for the review and how studies were grouped for the syntheses.	Page 3, lines 99-117 and Table 1
Information sources	6	Specify all databases, registers, websites, organisations, reference lists and other sources searched or consulted to identify studies. Specify the date when each source was last searched or consulted.	Page 4, lines 122-128
Search strategy	7	Present the full search strategies for all databases, registers and websites, including any filters and limits used.	Supplementary Tables S2A-D
Selection process	8	Specify the methods used to decide whether a study met the inclusion criteria of the review, including how many reviewers screened each record and each report retrieved, whether they worked independently, and if applicable, details of automation tools used in the process.	Page 4, lines 126-128
Data collection process	9	Specify the methods used to collect data from reports, including how many reviewers collected data from each report, whether they worked independently, any processes for obtaining or confirming data from study investigators, and if applicable, details of automation tools used in the process.	Page 4, line 130-131
Data items	10a	List and define all outcomes for which data were sought. Specify whether all results that were compatible with each outcome domain in each study were sought (e.g. for all measures, time points, analyses), and if not, the methods used to decide which results to collect.	Page 3, lines 106-112
	10b	List and define all other variables for which data were sought (e.g. participant and intervention characteristics, funding sources). Describe any assumptions made about any missing or unclear information.	Page 4, lines 131-134
Study risk of bias assessment	11	Specify the methods used to assess risk of bias in the included studies, including details of the tool(s) used, how many reviewers assessed each study and whether they worked independently, and if applicable, details of automation tools used in the process.	Page 4, lines 148-154

Section and Topic	Item #	Checklist item	Location where item is reported
Effect measures	12	Specify for each outcome the effect measure(s) (e.g. risk ratio, mean difference) used in the synthesis or presentation of results.	Page 4, lines 162-164
Synthesis methods	13a	Describe the processes used to decide which studies were eligible for each synthesis (e.g. tabulating the study intervention characteristics and comparing against the planned groups for each synthesis (item #5)).	Page 4, line 158
	13b	Describe any methods required to prepare the data for presentation or synthesis, such as handling of missing summary statistics, or data conversions.	Page 4, lines 145-146 and lines 158-159
	13c	Describe any methods used to tabulate or visually display results of individual studies and syntheses.	Page 4, lines 153-154, page 5 line 187 and line 190-191
	13d	Describe any methods used to synthesize results and provide a rationale for the choice(s). If meta-analysis was performed, describe the model(s), method(s) to identify the presence and extent of statistical heterogeneity, and software package(s) used.	Page 4, lines 160-162 and page 5 lines 180-187
	13e	Describe any methods used to explore possible causes of heterogeneity among study results (e.g. subgroup analysis, meta-regression).	Page 5, lines 170-175
	13f	Describe any sensitivity analyses conducted to assess robustness of the synthesized results.	Page 5, lines 175-177
Reporting bias assessment	14	Describe any methods used to assess risk of bias due to missing results in a synthesis (arising from reporting biases).	Page 5, lines 185-187
Certainty assessment	15	Describe any methods used to assess certainty (or confidence) in the body of evidence for an outcome.	Page 5, lines 189-191
**RESULTS**			
Study selection	16a	Describe the results of the search and selection process, from the number of records identified in the search to the number of studies included in the review, ideally using a flow diagram.	Page 5, lines 194-197 and Figure 1
	16b	Cite studies that might appear to meet the inclusion criteria, but which were excluded, and explain why they were excluded.	Table S3
Study characteristics	17	Cite each included study and present its characteristics.	Page 6, lines 202-214
Risk of bias in studies	18	Present assessments of risk of bias for each included study.	Page 6, lines 216-223, Figure 2 and Figures S1-S4
Results of	19	For all outcomes, present, for each study: (a) summary statistics for each group (where appropriate) and (b) an effect	Table S5

Section and Topic	Item #	Checklist item	Location where item is reported
individual studies		estimate and its precision (e.g. confidence/credible interval), ideally using structured tables or plots.	
Results of syntheses	20a	For each synthesis, briefly summarise the characteristics and risk of bias among contributing studies.	Figure 2 and Figures S1-S4
	20b	Present results of all statistical syntheses conducted. If meta-analysis was done, present for each the summary estimate and its precision (e.g. confidence/credible interval) and measures of statistical heterogeneity. If comparing groups, describe the direction of the effect.	Table 2, Figures S5-S21
	20c	Present results of all investigations of possible causes of heterogeneity among study results.	Figures S8, S10, S11, S12, S19
	20d	Present results of all sensitivity analyses conducted to assess the robustness of the synthesized results.	NA (explanation page 5, lines 175-177)
Reporting biases	21	Present assessments of risk of bias due to missing results (arising from reporting biases) for each synthesis assessed.	NA – funnel plot not made due to too few studies (see page 5, lines 185-187)
Certainty of evidence	22	Present assessments of certainty (or confidence) in the body of evidence for each outcome assessed.	Page 12, lines 351-553 and Tables S6-S8
**DISCUSSION**			
Discussion	23a	Provide a general interpretation of the results in the context of other evidence.	Pages 12-13, lines 365-404
	23b	Discuss any limitations of the evidence included in the review.	Pages 14-15, lines 488-493
	23c	Discuss any limitations of the review processes used.	Page 15, lines 493-506
	23d	Discuss implications of the results for practice, policy, and future research.	Page 14, lines 470-473
**OTHER INFORMATION**			
Registration and protocol	24a	Provide registration information for the review, including register name and registration number, or state that the review was not registered.	Page 3, lines 96-97
	24b	Indicate where the review protocol can be accessed, or state that a protocol was not prepared.	Page 3, lines 96-97

Section and Topic	Item #	Checklist item	Location where item is reported
	24c	Describe and explain any amendments to information provided at registration or in the protocol.	NA
Support	25	Describe sources of financial or non-financial support for the review, and the role of the funders or sponsors in the review.	Page 16, lines 543-544
Competing interests	26	Declare any competing interests of review authors.	Page 15, lines 539-540
Availability of data, code and other materials	27	Report which of the following are publicly available and where they can be found: template data collection forms; data extracted from included studies; data used for all analyses; analytic code; any other materials used in the review.	Page 15, lines 541-542

From: Page MJ, McKenzie JE, Bossuyt PM, Boutron I, Hoffmann TC, Mulrow CD, et al. The PRISMA 2020 statement: an updated guideline for reporting systematic reviews. BMJ 2021;372:n71. doi: 10.1136/bmj.n71.

Table S2A. Results from the systematic searches

Database	*Platform*	*Results*	*Date*
PubMed	PubMed.gov	310	March 7th 2025
Embase	Embase.com	738	March 7th 2025
Cochrane	Wiley	254	March 7th 2025
All<br>÷ duplicates with EndNote		1302<br>1026	

Table S2B. Search strategy in PubMed

Search	*Query*	*Results*
#8	Search: #6 NOT #7 Sort by: Publication Date	310
#7	Search: ("Animals"[Mesh]) NOT "Humans"[Mesh] Sort by: Publication Date	5,313,993
#6	Search: #4 AND #5 Sort by: Publication Date	383
#5	Search: "Randomized Controlled Trial"[pt] OR "Controlled Clinical Trial"[pt] OR "Pragmatic Clinical Trial"[pt] OR "Equivalence Trial"[pt] OR "Clinical Trial, Phase III"[pt] OR "Randomized Controlled Trials as Topic"[mh] OR "Controlled Clinical Trials as Topic"[mh] OR "Random Allocation"[mh] OR "Double-Blind Method"[mh] OR "Single-Blind Method"[mh] OR Placebos[Mesh:NoExp] OR "Control Groups"[mh] OR (random*[tiab] OR sham[tiab] OR placebo*[tiab]) OR ((singl*[tiab] OR doubl*[tiab]) AND (blind*[tiab] OR dumm*[tiab] OR mask*[tiab])) OR ((tripl*[tiab] OR trebl*[tiab]) AND (blind*[tiab] OR dumm*[tiab] OR mask*[tiab])) OR (control*[tiab] AND (study[tiab] OR studies[tiab] OR trial*[tiab] OR group*[tiab])) OR (Nonrandom*[tiab] OR "non random*"[tiab] OR "non-random*"[tiab] OR "quasi-random*"[tiab] OR quasirandom*[tiab]) OR allocated[tiab] OR (("open label"[tiab] OR "open-label"[tiab]) AND (study[tiab] OR studies[tiab] OR trial*[tiab])) OR ((equivalence[tiab] OR superiority[tiab] OR "non-inferiority"[tiab] OR noninferiority[tiab]) AND (study[tiab] OR studies[tiab] OR trial*[tiab])) OR ("pragmatic study"[tiab] OR "pragmatic studies"[tiab]) OR ((pragmatic[tiab] OR practical[tiab]) AND trial*[tiab]) OR ((quasiexperimental[tiab] OR "quasi-experimental"[tiab]) AND (study[tiab] OR studies[tiab] OR trial*[tiab])) OR (phase[ti] AND (III[ti] OR 3[ti]) AND (study[ti] OR studies[ti] OR trial*[ti])) OR (phase[ot] AND (III[ot] OR 3[ot]) AND (study[ot] OR studies[ot] OR trial*[ot])) Sort by: Publication Date	4,786,047
#4	Search: #1 AND #2 AND #3 Sort by: Publication Date	1,155
#3	Search: ((("Pregnancy Rate"[Mesh]) OR ("Pregnancy"[Mesh])) OR ("Endometrium"[Mesh])) OR (Pregnan*[Title/Abstract] OR Live birth*[Title/Abstract] OR abortion*[Title/Abstract] OR Miscarriage*[Title/Abstract] OR Endometr* thickness*[Title/Abstract] OR Endometr* receptivit*[Title/Abstract]) Sort by: Publication Date	1,259,975
#2	Search: ("Infertility, Female"[Mesh]) OR (Female infertilit*[Title/Abstract] OR Female fertilit*[Title/Abstract] OR Female reproducti*[Title/Abstract] OR Female subfertili*[Title/Abstract]) Sort by: Publication Date	50,589
#1	Search: (("Dietary Supplements"[Mesh]) OR (dietary suppl*[Title/Abstract])) OR (food supplement*[Title/Abstract])) OR (nutraceutic*[Title/Abstract])) OR (nutriceutic*[Title/Abstract])) OR (neutraceutic*[Title/Abstract])) OR (herbal supplement*[Title/Abstract])) OR (nutritional supplement*[Title/Abstract])) OR (("Micronutrients" [Pharmacological Action]) OR "Micronutrients"[Mesh])) OR (micronutrien*[Title/Abstract] OR trace element*[Title/Abstract] OR vitamin*[Title/Abstract])) OR (("Antioxidants" [Pharmacological Action]) OR "Antioxidants"[Mesh])) OR (antioxidant*[Title/Abstract]))	3,660,576

	OR (anti-oxidant*[Title/Abstract])) OR (probiotic*[Title/Abstract])) OR ("Vitamins" [Pharmacological Action])) OR ("Vitamin A"[Mesh])) OR ("Ascorbic Acid"[Mesh])) OR ("Vitamin D"[Mesh])) OR ("Vitamin E"[Mesh])) OR ("Vitamin K"[Mesh])) OR ("Thiamine"[Mesh])) OR ("Riboflavin"[Mesh])) OR ("Niacinamide"[Mesh])) OR ("Pantothenic Acid"[Mesh])) OR ("Vitamin B 6"[Mesh])) OR ("Biotin"[Mesh])) OR ("Folic Acid"[Mesh])) OR ("Vitamin B 12"[Mesh])) OR ("Sodium"[Mesh])) OR ("Potassium"[Mesh] OR "Potassium, Dietary"[Mesh])) OR ("Calcium"[Mesh])) OR ("Magnesium"[Mesh])) OR ("Phosphorus"[Mesh])) OR ("Chlorides"[Mesh])) OR ("Copper"[Mesh])) OR ("Iron, Dietary"[Mesh])) OR ("Chromium"[Mesh])) OR ("Molybdenum"[Mesh])) OR ("Zinc"[Mesh])) OR ("Manganese"[Mesh])) OR ("Selenium"[Mesh])) OR (ascorbic acid*[Title/Abstract] OR thiamin*[Title/Abstract] OR riboflavin*[Title/Abstract] OR niacin*[Title/Abstract] OR pantothenic*[Title/Abstract] OR biotin*[Title/Abstract] OR folic acid*[Title/Abstract] OR folate*[Title/Abstract] OR cobalamin*[Title/Abstract] OR sodium*[Title/Abstract] OR potassium*[Title/Abstract] OR calcium*[Title/Abstract] OR magnesi*[Title/Abstract] OR phosphorus*[Title/Abstract] OR chloride*[Title/Abstract] OR copper*[Title/Abstract] OR iron[Title/Abstract] OR iodine*[Title/Abstract] OR chromium*[Title/Abstract] OR molybdenum*[Title/Abstract] OR zinc*[Title/Abstract] OR manganese*[Title/Abstract] OR selenium*[Title/Abstract])) OR ("Fish Oils"[Mesh])) OR (fish oil*[Title/Abstract] OR omega 3[Title/Abstract] OR docosahexaenoic*[Title/Abstract] OR eicosapentaenoic*[Title/Abstract])) OR ("Carnitine"[Mesh])) OR (carnitine*[Title/Abstract])) OR (acetylcarnitine*[Title/Abstract])) OR ("Acetylcysteine"[Mesh])) OR (acetylcysteine*[Title/Abstract] OR acetyl-L-cysteine*[Title/Abstract])) OR ("Ubiquinone"[Mesh])) OR (ubiquinone[Title/Abstract] OR q10[Title/Abstract] OR coQ 10[Title/Abstract])) OR ((((("Glutathione"[Mesh]) OR "Arginine"[Mesh]) OR "Lycopene"[Mesh]) OR "beta Carotene"[Mesh]) OR "Choline"[Mesh])) OR (glutathione*[Title/Abstract] OR arginine*[Title/Abstract] OR lycopene*[Title/Abstract] OR beta carotene*[Title/Abstract] OR betacarotene*[Title/Abstract] OR choline*[Title/Abstract])) OR (("Sodium Chloride, Dietary"[Mesh] OR "Phosphorus, Dietary"[Mesh] OR "Sodium, Dietary"[Mesh] OR "Calcium, Dietary"[Mesh])) Sort by: Publication Date	

Search filter used: RCT / CCT - PubMed. In: Canada's Drug Agency Search Filters Database. Ottawa: Canada's Drug Agency; 2025: https://searchfilters.cda-amc.ca/link/108. Accessed 2025-03-07.

Table S2C. Search strategy in Embase

No.	***Query***	***Results***
#7	#6 NOT ('animal'/exp NOT 'human'/exp)	738
#6	#4 AND #5	945
#5	'controlled clinical trial'/exp OR 'randomized controlled trial'/exp OR (((random* OR controlled* OR crossover OR 'cross over' OR blind* OR mask*) NEAR/3 (trial* OR study OR studies OR analy*)):ti,ab,de) OR rct:ti,ab,de OR (((single OR double OR triple) NEAR/2 (blind* OR mask*)):ti,ab,de) OR placebo:ti,ab,de	11922112
#4	#1 AND #2 AND #3	2216
#3	'pregnancy rate'/de OR 'pregnancy'/exp OR 'pregnancy outcome'/de OR 'live birth'/de OR 'endometrial thickness'/de OR 'endometrial thickening'/de OR 'abortion'/exp OR 'endometrial receptivity'/de OR pregnan*:ti,ab,kw OR 'live birth*':ti,ab,kw OR abortion*:ti,ab,kw OR miscarriage*:ti,ab,kw OR 'endometrial thickness*':ti,ab,kw OR 'endometrial receptivit*':ti,ab,kw	1334340
#2	'dietary supplement'/exp OR 'diet supplementation'/exp OR (((diet* OR food OR herbal OR nutrition*) NEAR/1 suppl*):ti,ab,kw) OR neutraceutic*:ti,ab,kw OR nutraceutic*:ti,ab,kw OR nutriceutic*:ti,ab,kw OR 'nutraceutical'/exp OR 'trace element'/exp OR micronutrient*:ti,ab,kw OR 'trace element*':ti,ab,kw OR	4317123

	vitamin*:ti,ab,kw OR 'antioxidant'/exp OR 'vitamin'/exp OR antioxidant*:ti,ab,kw OR 'anti-oxidant*':ti,ab,kw OR probiotic*:ti,ab,kw OR 'probiotic agent'/exp OR 'sodium'/exp OR 'potassium'/exp OR 'potassium intake'/exp OR 'chloride'/exp OR 'mineral intake'/exp OR 'chromium'/exp OR 'molybdenum'/exp OR 'zinc'/exp OR 'manganese'/exp OR 'selenium'/exp OR 'ascorbic acid*':ti,ab,kw OR thiamin*:ti,ab,kw OR riboflavin*:ti,ab,kw OR niacin*:ti,ab,kw OR pantothenic*:ti,ab,kw OR biotin*:ti,ab,kw OR 'folic acid*':ti,ab,kw OR folate*:ti,ab,kw OR cobalamin*:ti,ab,kw OR sodium*:ti,ab,kw OR potassium*:ti,ab,kw OR calcium*:ti,ab,kw OR magnesi*:ti,ab,kw OR phosphorus*:ti,ab,kw OR chloride*:ti,ab,kw OR copper*:ti,ab,kw OR iron:ti,ab,kw OR iodine*:ti,ab,kw OR chromium*:ti,ab,kw OR molybdenum*:ti,ab,kw OR zinc*:ti,ab,kw OR manganese*:ti,ab,kw OR selenium*:ti,ab,kw OR 'fish oil'/exp OR 'fish oil*':ti,ab,kw OR 'omega 3':ti,ab,kw OR docosahexaenoic*:ti,ab,kw OR eicosapentaenoic*:ti,ab,kw OR 'carnitine'/exp OR 'acetylcysteine'/exp OR 'ubiquinone'/exp OR 'glutathione'/exp OR 'arginine'/exp OR 'choline'/exp OR carnitine*:ti,ab,kw OR acetylcarnitine*:ti,ab,kw OR acetylcysteine*:ti,ab,kw OR 'acetyl-l-cysteine*':ti,ab,kw OR ubiquinone:ti,ab,kw OR q10:ti,ab,kw OR 'coq 10':ti,ab,kw OR glutathione*:ti,ab,kw OR arginine*:ti,ab,kw OR lycopene*:ti,ab,kw OR 'beta carotene*':ti,ab,kw OR betacarotene*:ti,ab,kw OR choline*:ti,ab,kw OR 'sodium intake'/de OR 'phosphate intake'/de OR 'salt intake'/de OR 'calcium intake'/de	
#1	'female infertility'/exp OR ((female NEAR/1 (infertilit* OR fertilit* OR reproducti* OR subfertilit*)):ti,ab,kw)	83140

Table S2D. Search strategy in the Cochrane Central Register of Controlled Trials

ID	***Search***	***Hits***
#1	MeSH descriptor: [Infertility, Female] explode all trees	1994
#2	(female NEAR/1 (infertilit* OR fertilit* OR reproduct* OR subfertili*)):ti,ab,kw	4840
#3	#1 OR #2	4840
#4	MeSH descriptor: [Dietary Supplements] explode all trees	19399
#5	((diet* OR food OR herbal OR nutrition*) NEAR/1 suppl*):ti,ab,kw	30538
#6	(neutraceutic* OR nutraceutic* OR nutriceutic*):ti,ab,kw	1073
#7	MeSH descriptor: [Micronutrients] explode all trees	7075
#8	(micronutrien* OR (trace NEXT element*) OR vitamin*):ti,ab,kw	41903
#9	MeSH descriptor: [Antioxidants] explode all trees	6418
#10	(antioxidant* OR (anti NEXT oxidant*) OR probiotic*):ti,ab,kw	28027
#11	MeSH descriptor: [Vitamins] explode all trees	5680
#12	MeSH descriptor: [Carotenoids] explode all trees	4603
#13	MeSH descriptor: [Vitamin B 12] explode all trees	1159
#14	MeSH descriptor: [Vitamin B 6] explode all trees	862
#15	MeSH descriptor: [Ascorbic Acid] explode all trees	2786
#16	MeSH descriptor: [Vitamin D] explode all trees	7592
#17	MeSH descriptor: [Vitamin E] explode all trees	3061
#18	MeSH descriptor: [Vitamin K] explode all trees	854
#19	MeSH descriptor: [Thiamine] explode all trees	390
#20	MeSH descriptor: [Riboflavin] explode all trees	560
#21	MeSH descriptor: [Niacinamide] explode all trees	1625
#22	MeSH descriptor: [Pantothenic Acid] explode all trees	123
#23	MeSH descriptor: [Biotin] explode all trees	69
#24	MeSH descriptor: [Folic Acid] explode all trees	4685
#25	MeSH descriptor: [Sodium] explode all trees	2742
#26	MeSH descriptor: [Potassium] explode all trees	2673
#27	MeSH descriptor: [Calcium] explode all trees	4700

#28	MeSH descriptor: [Magnesium] explode all trees	1520
#29	MeSH descriptor: [Phosphorus] explode all trees	948
#30	MeSH descriptor: [Chlorides] explode all trees	3488
#31	MeSH descriptor: [Copper] explode all trees	623
#32	MeSH descriptor: [Iron, Dietary] explode all trees	463
#33	MeSH descriptor: [Chromium] explode all trees	434
#34	MeSH descriptor: [Molybdenum] explode all trees	36
#35	MeSH descriptor: [Zinc] explode all trees	2040
#36	MeSH descriptor: [Manganese] explode all trees	98
#37	MeSH descriptor: [Selenium] explode all trees	956
#38	((ascorbic NEXT acid*) OR thiamin* OR riboflavin* OR niacin* OR pantothenic* OR biotin* OR (folic NEXT acid*) OR folate* OR cobalamin* OR sodium* OR potassium* OR calcium* OR magnesi* OR phosphorus* OR chloride* OR copper* OR iron OR iodine* OR chromium* OR molybdenum* OR zinc* OR manganese* OR selenium*):ti,ab,kw	135097
#39	MeSH descriptor: [Fish Oils] explode all trees	4614
#40	((fish NEXT oil*) OR "omega 3" OR docosahexaenoic* OR eicosapentaenoic*):ti,ab,kw	10675
#41	MeSH descriptor: [Carnitine] explode all trees	809
#42	MeSH descriptor: [Acetylcysteine] explode all trees	1446
#43	MeSH descriptor: [Ubiquinone] explode all trees	713
#44	MeSH descriptor: [Glutathione] explode all trees	853
#45	MeSH descriptor: [Arginine] explode all trees	1831
#46	MeSH descriptor: [Choline] explode all trees	1742
#47	(carnitine* OR acetylcarnitine* OR acetylcysteine* OR "acetyl-L-cysteine" OR ubiquinone OR q10 OR "coQ 10" OR glutathione* OR arginine* OR lycopene* OR (beta NEXT carotene*) OR betacarotene* OR choline*):ti,ab,kw	24589
#48	MeSH descriptor: [Sodium, Dietary] explode all trees	1010
#49	MeSH descriptor: [Phosphorus, Dietary] explode all trees	101
#50	MeSH descriptor: [Calcium, Dietary] explode all trees	1719
#51	{OR #4-#50}	222931
#52	MeSH descriptor: [Pregnancy] explode all trees	33253
#53	MeSH descriptor: [Pregnancy Rate] explode all trees	2370
#54	MeSH descriptor: [Endometrium] explode all trees	1340
#55	(pregnan* OR (live NEXT birth*) OR abortion* OR miscarriage* OR (endometrial NEAR/1 (thickness* OR receptivit*))):ti,ab,kw	96599
#56	{OR #52- #55}	273578
#57	#3 AND #51 AND #56	266
#58	MeSH descriptor: [Animals] explode all trees	887757
#59	MeSH descriptor: [Humans] explode all trees	884049
#60	#58 NOT #59	3708
#61	#57 NOT #60 in Trials	254

Table S3. Reasons for exclusion of identified articles based on full text screening.

Authors	***Title***	***Year***	***Population***	***Intervention***	***Comparator***	***Outcome***	***Study design***	***Publication type***	***Other***	***Specified reason for exclusion***
Aflatoonian, A., Arabjahvani, F., Eftekhar, M., Sayadi, M.	Effect of vitamin D insufficiency treatment on fertility outcomes in frozen-thawed embryo transfer cycles: A randomized clinical trial	2014	X							Includes male factor and combined male and female factor infertility.
Ainehchi, N., Khaki, A., Farshbaf-Khalili, A., Hammadeh, M., Ouladsahebmadarek, E.	The effectiveness of herbal mixture supplements with and without clomiphene citrate in comparison to clomiphene citrate on serum antioxidants and glycemic biomarkers in women with polycystic ovary syndrome willing to be pregnant: A randomized clinical trial	2019	X							Includes male factor infertility.
Ajam, K. A., Farzadi, L., Nouri, M., Sadagheani, M. M.	The effect of nitric oxide with minimal stimulation on patients with polycystic ovarian syndrome	2014		X						Nitric oxide is administered vaginally.
Aksoy, A. N., Kadanali, S.	Evaluating the effects of vitamin E addition to clomiphene citrate on endometrial receptivity: A prospective controlled study	2010					X			Not an RCT study.
Ala, S., Payvandi, S., Barzin, M., Tavajoh, M., Samaei, H.	Efficacy of Omega-3, -6, and -9 Fatty Acids, Alone or in Combination With Low Dose Aspirin, in Improvement of Uterine Blood Flow in Women With History of Recurrent Miscarriage: A Prospective, Randomized, Clinical Trial	2022			X	X				Comparator group receives aspirin; Not relevant outcomes.
Al-Alousi, T. A., Al-Allak, M. M. A., Aziz, A. A., Al Ghazali, B. S.	The effect of omega-3 on the number of retrieved ova, fertilization rate, and embryo grading in subfertile women undergoing intracytoplasmic sperm injection	2018	X							Includes male factor infertility.
Alhussien, Z. A., Mossa, H. A. L., Abood, M. S.	EFFECT OF L CARNITINE ON PREGNANCY OUTCOMES IN WOMEN WITH POLYCYSTIC OVARIAN SYNDROME	2022					X			Not an RCT study

	UNDERGONE INTRACYTOPLASMIC SPERM INJECTION									
Alquzwini, T. S.	The use of a combination of choline and inositol in the treatment of infertility in women with the polycystic ovarian syndrome	2020			X		X			Not an RCT study; no comparator group.
Aqrawi, J. G., Al-Qadhi, H. I., Al-Asadi, F. A.	Study of N-Acetyl Cysteine Plus Metformin Versus Metformin Alone in Treatment of Iraqi Women with Polycystic Ovarian Syndrome	2022	X							Not all included participants are infertile; includes women who were virgins.
Asadi, M., Matin, N., Frootan, M., Mohamadpour, J., Qorbani, M., Tanha, F. D.	Vitamin D improves endometrial thickness in PCOS women who need intrauterine insemination: a randomized double-blind placebo-controlled trial	2014		X						Vitamin D is administered as a gluteal injection.
Ashoush S, Abou-Gamrah A, Bayoumy H, Othman N	Chromium picolinate reduces insulin resistance in polycystic ovary syndrome: Randomized controlled trial	2016	X							Identified from hand-search of included studies. The study includes participants with PCOS, however it does not state that these should be infertile. In results, it is stated that infertility was not present in all participants and the outcome of interest (pregnancy) is therefore only reported on a subpopulation.
Attallah, D., El-Nashar, I. H., Mahmoud, R., Shaaban, O. M., Salman, S. A.	N-acytelcysteinepriortointrauterineinsemination in couples with isolated athenozospermia: a randomized controlled tria	2013	X					X		Is an abstract. Male partners are the participants that receives the intervention.
Badawy, A., Baker El Nashar, A., El Totongy, M.	Clomiphene citrate plus N-acetyl cysteine versus clomiphene citrate for augmenting ovulation in the management of unexplained infertility: a randomized double-blind controlled trial	2006							X	The publication has been retracted by the journal.
Badawy, A., State, O., Abdelgawad, S.	N-Acetyl cysteine and clomiphene citrate for induction of ovulation in polycystic ovary syndrome: a cross-over trial	2007					X			Not an RCT study.
Bezerra Espinola, M. S., Bilotta, G., Aragona, C.	Positive effect of a new supplementation of vitamin D(3) with myo-inositol, folic acid and melatonin on IVF outcomes: a prospective randomized and controlled pilot study	2021	X							Includes male factor and combined male and female factor infertility.

Bhatti, Z. I., Choudhery, K. A., Yasin, I., Assan, M. H., Bashir, F.	Role of Vitamin D supplements in improving fertility among sub fertile couples	2019	X							Includes male factor and combined male and female factor infertility.
Chernukha, G. E., Tabeeva, G. I., Udovichenko, M. A.	Unused opportunities for correction of endocrine and metabolic disorders in polycystic ovary syndrome	2019							X	Article not written in the Latin alphabet.
Chi, Ctr Ipr. (https://www.cochranelibrary.com/central/doi/10.1002/central/CN-01830000/full)	The improvement of endometrial receptivity in PCOS patients by Vitamin D3: a prospective randomized controlled clinical trial	2015						X		Trial registration. No article has been identified.
Ciotta L, Stracquadanio M, Pagano I, Carbonaro A, Palumbo M, Gulino F	Effects of myo-inositol supplementation on oocyte's quality in PCOS patients: a double blind trial	2011	X							Identified from hand-search of included studies. "The ICSI or IVF procedures were recommended after evaluation of the sperm semen of the male partner" - male factor infertility is included.
Colazingari S, Treglia M, Najjar R, Bevilacqua A.	The combined therapy myo-inositol plus D-chiro-inositol, rather than D-chiro-inositol, is able to improve IVF outcomes: results from a randomized controlled trial.	2013	X							Identified from hand-search of included studies. Participants were recommended ICSI based on partner's sperm sample; includes male factor infertility.
Comhaire, F. H., Ecleer, W. D.	Treatment modalities of the infertile couple: Number needed to treat	2013							X	Numbers needed to treat are calculated based on aggregated data from multiple studies.
Comhaire, F., Decleer, W.	Comparing the effectiveness of infertility treatments by numbers needed to treat (NNT)	2012							X	Numbers needed to treat are calculated based on aggregated data from multiple studies.
Conforti, A., Iorio, G. G., Di Girolamo, R., Rovetto, M. Y., Picarelli, S., Cariati, F., Gentile, R., D'Amato, A., Gliozheni, O., Fioretti, B., Alviggi, C.	The impact of resveratrol on the outcome of the in vitro fertilization: an exploratory randomized placebo-controlled trial	2024	X							Includes male factor infertility
Crha, I., Hrubá, D., Ventruba, P., Fiala, J., Totusek, J., Visnová, H.	Ascorbic acid and infertility treatment	2003					X			Not an RCT study.
Ctri. (https://www.cochranelibrary.com/central/doi/10.1002/central/CN-02498278/full)	Myoinostol,Vitamin D3 and clomiphene citrate for Ovulation induction in infertile women with Polycystic Ovarian syndrome- A Randomized control trial	2022						X		Trial registration. No article has been identified.
Dastorani, M., Aghadavod, E., Mirhosseini, N., Foroozanfard,	The effects of vitamin D supplementation on metabolic profiles and gene expression of	2018				X				Not relevant outcomes.

F., Zadeh Modarres, S., Amiri Siavashani, M., Asemi, Z.	insulin and lipid metabolism in infertile polycystic ovary syndrome candidates for in vitro fertilization									
Doryanizadeh, L., Morshed-Behbahani, B., Parsanezhad, M. E., Dabbaghmanesh, M. H., Jokar, A.	Calcitriol Effect on Outcomes of in Vitro Fertilization in Infertile Women with Vitamin D Deficiency: A Double-Blind Randomized Clinical Trial	2021	X							Includes male factor and combined male and female factor infertility.
Drug and Therapeutics Bulletin	Antioxidant supplements do not improve conception rates	2013					X			Summary of information; not an RCT study.
Du, Y., Zhao, Y., Ma, Y., Bai, H., Li, X.	Clinical observation on treatment of 2,062 cases of immune infertility with integration of traditional Chinese medicine and western medicine	2005			X		X			Not an RCT; no comparator group.
El Refaeey, A., Selem, A., Badawy, A.	Combined coenzyme Q10 and clomiphene citrate for ovulation induction in clomiphene-citrate-resistant polycystic ovary syndrome	2014							X	The publication has been retracted by the journal.
El Sharkwy I, Sharaf El-Din M.	l-Carnitine plus metformin in clomiphene-resistant obese PCOS women, reproductive and metabolic effects: a randomized clinical trial.	2019							X	The publication has been retracted by the journal.
EL-Gharib, Mohamed & Osman, Aya	N-Acetyl Cysteine, Chromium Picolinate: Adjuvant to Clomiphene Therapy of PCOS	2014					X			Identified from hand-search of included studies. Is not an RCT study.
El-Khayat, W., Elsadek, M.	Calcium infusion for the prevention of ovarian hyperstimulation syndrome: a double-blind randomized controlled trial	2015	X	X						Calcium is given as an infusion; includes male factor infertility.
Fereidouni, F., Kashani, L., Amidi, F., Khodarahmian, M., Zhaeentan, S., ardehjani, N. A., Rastegar, T.	Astaxanthin treatment decreases pro-inflammatory cytokines and improves reproductive outcomes in patients with polycystic ovary syndrome undergoing assisted reproductive technology: A randomized clinical trial	2024	X							The articles only exclude women with partners having severe male factor infertility; includes male factor infertility.
Fernando, S., Osianlis, T., Vollenhoven, B., Wallace, E., Rombauts, L.	A pilot double-blind randomised placebo-controlled dose-response trial assessing the effects of melatonin on infertility treatment (MIART): study protocol	2014	X							Study protocol. The article "Melatonin in Assisted Reproductive Technology: A Pilot Double-Blind Randomized Placebo-Controlled Clinical Trial" by Fernando et al. (2018) has been identified. Excluded as the study includes male factor infertility.

Fusi, F. M., Ferrario, M., Bosisio, C., Arnoldi, M., Zanga, L.	DHEA supplementation positively affects spontaneous pregnancies in women with diminished ovarian function	2013					X			Observational cohort study.
Gerli, S., Della Morte, C., Ceccobelli, M., Mariani, M., Favilli, A., Leonardi, L., Lanti, A., Iannitti, R. G., Fioretti, B.	Biological and clinical effects of a resveratrol-based multivitamin supplement on intracytoplasmic sperm injection cycles: a single-center, randomized controlled trial	2022	X							The study only excludes couples with severe male factor infertility.
Gharaei R, Alyasin A, Mahdavinezhad F, Samadian E, Ashrafnezhad Z, Amidi F	Randomized controlled trial of astaxanthin impacts on antioxidant status and assisted reproductive technology outcomes in women with polycystic ovarian syndrome.	2022	X							Identified from hand-search of included studies. States in methods that the couples did not have a background of severe male factor infertility; allows inclusion of male factor infertility.
Gopinath, M., Khadijah, I. S., Ruhaima, R., Nuguelis, R., Mukhri, H.	The impact of oral multinutrient supplementation on in vitro fertilisation or intracytoplasmic sperm injection outcomes: A prospective controlled study	2024	X							Includes male factor infertility
Hrubá, D., Crha, I., Fiala, J., Totuśek, J., Brázdová, Z., Ventruba, P.	Ascorbic acid in the treatment of infertility (less known importance of vitamin C)	2003					X			Not an RCT study.
Hu, K. L., Gan, K., Wang, R., Li, W., Wu, Q., Zheng, B., Zou, L., Zhang, S., Liu, Y., Wu, Y., Chen, R., Cao, W., Yang, S., Liu, F. T., Tian, L., Zeng, H., Xu, H., Qiu, S., Yang, L., Chen, X., Pan, X., Wu, X., Mol, B. W., Li, R., Zhang, D.	Vitamin D supplementation prior to in vitro fertilisation in women with polycystic ovary syndrome: a protocol of a multicentre randomised, double-blind, placebo-controlled clinical trial	2020						X		Study protocol. No article has been identified.
Irct201009131760N.	Assessment the effect of Ca-Vitamin D and metformin on PCOS	2011						X		Trial registration. No article has been identified.
Irct201207156420N.	Treatment of vitamin D deficiency in assisted reproductive cycles	2013	X							Trial registration. The article "Effect of vitamin D insufficiency treatment on fertility outcomes in frozen-thawed embryo transfer cycles: A randomized clinical trial" by Aflatoonian et al. (2014) has been identified from this trial registration. Excluded as the study includes male factor and combined male and female factor infertility.

Irct20130115012146N	The effect of adding L-Arginine in infertile patients with refractory thin endometrium	2024	X							Trial registration. The article "Impact of L-arginine Supplementation on Endometrial Thickness in Infertile Patients with Refractory Thin Endometrium: A Randomized Controlled Trial" by Shokri et al. (2025) has been identified. Excluded as it includes male factor infertility.
Irct20130603013566N.	Comparing the results of ovulation stimulationDouble or dual stimulation in poor ovarian responders	2023						X		Trial registration. No article has been identified.
Irct201306115942N.	Effects of antioxidative treatments on pregnancy results	2013							X	Trial registration. The article "Effects of antioxidative treatments on sperm DNA fragmentation and pregnancy results in IUI" by Sadeghpour et al. (2015) has been identified from this trial registration. Excluded as the article is not written in the Latin alphabet.
Irct2014050617593N.	N-acetylcysteine in treatment of infertile women with polycystic ovarian syndrome	2015							X	Trial registration. The article "Effect of N-acetylcysteine with letrozole to induction of ovulation in infertile women with polcystic ovarian syndrom" by Ghale TD et al. (2016) has been identified. Article not written in the Latin alphabet.
Irct201405286541N.	Effect of vitamin E in out come of IVF	2014						X		Trial registration. No article has been identified.
Irct20150831023831N.	Effect of N-acetyl cysteine on expression of Oxidation-Reduction genes during implantation window in women with Recurrent Implantation Failure	2018						X		Trial registration. No article has been identified.
Irct2015102724746N.	Q10 co-enzyme effect on fetus implantation	2015	X							The article "Q10 Co-enzyme Effect on Fetus Implantation in ART Cycles" by Ghasemzadeh et al. (2020) has been identified from this trial registration. Excluded as the study includes male factor infertility.
Irct20151123025202N.	In Vitro Fertilization	2020							X	The article “The Boosting Effects of Melatonin on the IVF of Women with PCOS” by Pilehvari S, et al. (2023) has

										been identified from this trial registration. Unclear whether both treatment arms receive folic acid. Corresponding author has been contacted, no response.
Irct20181225042109N.	The effect of Myo-inositol on pregnancy success	2019							X	The article "Myo-inositol effect on pregnancy outcomes in infertile women undergoing in vitro fertilization/intracytoplasmic sperm injection: A double-blind RCT" by Seyedoshohadaei et al. (2021) has been identified from this trial registration. The article is already included in the search.
Irct20210820052235N.	The effect of vitamin D on the outcome of IVF therapy in women with poor response to IVF treatment	2022						X		Trial registration. No article has been identified.
Irct20211204053267N.	The effect of administering L-Carnitine to Clomiphene citrate stimulated cycles on conception rate and ovulation in infertile women with polycystic ovary syndrome	2021						X		Trial registration. No article has been identified.
Irct20220317054318N.	The effect of oral L-arginine consumption on uterine artery resistance in women with recurrent implant failure	2022						X		Trial registration. No article has been identified.
Irct20230123057195N	The effect of Lactofem on the success of the IUI cycle	2024						X		Trial registration. No article has been identified.
Irct20240119060732N	The effect of Myoinositol on the outcomes of assisted reproductive treatments in patients with polycystic ovary syndrome	2024						X		Trial registration. No article has been identified.
Irct20240415061493N	Evaluation of melatonin supplementation on infertile couples	2024						X		Trial registration. No article has been identified.
Islamova, H. O.	Pregravid preparation of women with polycystic ovary syndrome: Research of the effectiveness and safety of the Myofolik complex drug	2020							X	Article not written in the Latin alphabet.
Ismail, A. M., Hamed, A. H., Saso, S., Thabet, H. H.	Adding L-carnitine to clomiphene resistant PCOS women improves the quality of ovulation and the pregnancy rate. A randomized clinical trial	2014							X	The publication has been retracted by the journal.
Isrctn. (https://www.cochranelibrary.co	A randomised controlled trial to compare conception rates for preconceptional folic acid	2007						X		Trial registration. No article has been identified.

m/central/doi/10.1002/central/CN-01829565/full)	400 mg daily versus Pregnacare Plus in assisted conception									
Jamal, H., Waheed, K., Mazhar, R., Sarwar, M. Z.	Comparative Study Of Combined Co-Enzyme Q10 And Clomiphene Citrate Vs Clomiphene Citrate Alone For Ovulation Induction In Patients With Polycystic Ovarian Syndrome	2023	X			X				The participants are not described as being infertile; not relevant outcomes.
Jirge, P. R., Chougule, S. M., Gavali, V. G., Bhomkar, D. A.	Impact of dehydroepiandrosterone on clinical outcome in poor responders: A pilot study in women undergoing in vitro fertilization, using bologna criteria	2014					X			Prospective case-control study, not an RCT.
Khmil, M., Khmil Doswald, A., Halnykina, S., Khmil, S.	Hormonal profile in women with infertility against the background of polycystic ovary syndrome depending on the type of pre-pregnancy pre-treatment	2022	X							Includes male factor infertility.
Khodarahmian, M., Amidi, F., Moini, A., Kashani, L., Salahi, E., Danaii-Mehrabad, S., Nashtaei, M. S., Mojtahedi, M. F., Esfandyari, S., Sobhani, A.	A randomized exploratory trial to assess the effects of resveratrol on VEGF and TNF-α 2 expression in endometriosis women	2021				X				Not relevant outcomes.
Kitaya, K., Ishikawa, T.	Genital tract dysbiosis in infertile women with a history of repeated implantation failure and pilot study for reproductive outcomes following oral enteric coating lactoferrin supplementation	2022					X			Prospective cohort study, not an RCT.
Ko, J. K. Y., Yung, S. S. F., Lai, S. F., Wan, R. S. F., Wong, C. K. Y., Wong, K., Cheung, C. L., Ng, E. H. Y., Raymond, H. W. Li	Effect of vitamin D in addition to letrozole on the ovulation rate of women with polycystic ovary syndrome: protocol of a multicentre randomised double-blind controlled trial	2024						X		Abstract. No article has been identified.
Konje, J. C.	CPD questions for volume 24 issue 4	2022							X	Not a study.
Kuroda, K., Horikawa, T., Gekka, Y., Moriyama, A., Nakao, K., Juen, H., Takamizawa, S., Ojiro, Y., Nakagawa, K., Sugiyama, R.	Effects of Periconceptional Multivitamin Supplementation on Folate and Homocysteine Levels Depending on Genetic Variants of Methyltetrahydrofolate Reductase in Infertile Japanese Women	2021	X				X			Not an RCT study; Includes male factor infertility.
Kuru Pekcan, M., Tokmak, A., Özaksit, G.	Effect of short term supplementation of coenzyme q10 on cumulative pregnancy rates in young infertilewomen with polycystic ovarian syndrome	2019					X			Non-randomized study.

Lesoine B, Regidor PA.	Prospective Randomized Study on the Influence of Myoinositol in PCOS Women Undergoing IVF in the Improvement of Oocyte Quality, Fertilization Rate, and Embryo Quality	2016				X				Identified from hand-search of included studies. Not relevant outcomes.
Li, C. J., Lin, L. T., Tsui, K. H.	Dehydroepiandrosterone shifts energy metabolism to increase mitochondrial biogenesis in female fertility with advancing age	2021					X			Not an RCT study.
Lian, F., Zhao, B., Lu, X. M.	[Effect of er'zhi tiangui granule on metabonomics and level of Ca2+ in follicle fluid in patients after in vitro fertilization and embryo transfer]	2010							X	Article not written in the Latin alphabet.
Lin, P. H., Su, W. P., Li, C. J., Lin, L. T., Sheu, J. J. C., Wen, Z. H., Cheng, J. T., Tsui, K. H.	Investigating the Role of Ferroptosis-Related Genes in Ovarian Aging and the Potential for Nutritional Intervention	2023				X				Not relevant outcomes.
Lisi F, Carfagna P, Oliva MM, Rago R, Lisi R, Poverini R, Manna C, Vaquero E, Caserta D, Raparelli V, Marci R, Moscarini M.	Pretreatment with myo-inositol in non polycystic ovary syndrome patients undergoing multiple follicular stimulation for IVF: a pilot study	2012	X							Identified from hand-search of included studies. The participants were recommended ICSI or IVF based on their partner's semen; includes male factor infertility.
Lisi, F., Carfagna, P., Oliva, M. M., Rago, R., Lisi, R., Poverini, R., Manna, C., Vaquero, E., Caserta, D., Raparelli, V., et al.	Pretreatment with myo-inositol in non polycystic ovary syndrome patients undergoing multiple follicular stimulation for IVF: a pilot study	2012	X							Patients were recommended either IVF or ICSI based on partner's semen sample; includes male factor infertility.
Ma, Y. C., Hao, G. M., Zhao, Z. M., Cui, N., Fan, Y. L., Zhang, S. C., Chen, J. W., Cao, Y. C., Guan, F. L., Geng, J. R., Gao, B. L., Du, H. L.	Effects of Bushen-Tiaojing-Fang on the pregnancy outcomes of infertile patients with repeated controlled ovarian stimulation	2021	X							Includes male factor and combined male and female factor infertility.
Ma, Y., Song, J., Cao, X., Sun, Z.	Mechanism of Guilu Erxian ointment based on targeted metabolomics in intervening in vitro fertilization and embryo transfer outcome in older patients with poor ovarian response of kidney-qi deficiency type	2023	X							Includes male factor and combined male and female factor infertility.
Maged AM, Elsawah H, Abdelhafez A, Bakry A, Mostafa WA.	The adjuvant effect of metformin and N-acetylcysteine to clomiphene citrate in induction of ovulation in patients with Polycystic Ovary Syndrome	2015							X	The publication has been retracted by the journal.

Mendoza, N., Diaz-Ropero, M. P., Aragon, M., Maldonado, V., Llaneza, P., Lorente, J., Mendoza-Tesarik, R., Maldonado-Lobon, J., Olivares, M., Fonolla, J.	Comparison of the effect of two combinations of myo-inositol and D-chiro-inositol in women with polycystic ovary syndrome undergoing ICSI: a randomized controlled trial	2019					X			The aim of the study is to evaluate the effect of two different formulations; both groups receive the supplement.
Mohammadi, S., Eini, F., Bazarganipour, F., Taghavi, S. A., Kutenaee, M. A.	The Effect of Myo-inositol on Fertility Rates in Poor Ovarian Responder in Women Undergoing Assisted Reproductive Technique: a Randomized Clinical Trial	2023							X	Abstract based on the article "The effect of Myo-inositol on fertility rates in poor ovarian responder in women undergoing assisted reproductive technique: a randomized clinical trial" by Mohammadi et al. (2021) which is already included in the search.
Naram, S., Mahajan, D., Parekh, H.	STREE SANJIVANI TABLET: An effective polyherbal combination to treat female gynaecological disorders	2022								Report not retrieved.
Nasr A.	Effect of N-acetyl-cysteine after ovarian drilling in clomiphene citrate-resistant PCOS women: a pilot study	2010							X	The publication has been retracted by the journal.
Nazari, L., Salehpour, S., Hosseini, S., Saharkhiz, N., Azizi, E., Hashemi, T., Ghodssi-Ghassemabadi, R.	Effect of myo-inositol supplementation on ICSI outcomes among poor ovarian responder patients: A randomized controlled trial	2020	X							The article only excludes women with partners having severe oligoasthenozoospermia or azoospermia; includes male factor infertility.
Nct. (https://www.cochranelibrary.com/central/doi/10.1002/central/CN-01515433/full)	DHEA Supplementation for Low Ovarian Response IVF Patients	2007						X		Trial registration. No article has been identified.
Nct. (https://www.cochranelibrary.com/central/doi/10.1002/central/CN-01562732/full)	Can Antioxidants Affect Pregnancy Rate in Patients With Expected Low Number of Egg Retrieval in IVF Cycles?	2017						X		Trial registration. No article has been identified.
Nct. (https://www.cochranelibrary.com/central/doi/10.1002/central/CN-01564747/full)	Micronutrient Supplementation in PCO-syndrome	2017	X			X				The article "The impact of a standardized micronutrient supplementation on PCOS-typical parameters: a randomized controlled trial" by Hager et al. (2019) has been identified from this trial registration. Excluded as the study population is not

										explicitly defined as being infertile; not relevant outcomes.
Nct. (https://www.cochranelibrary.com/central/doi/10.1002/central/CN-01661408/full)	Spontaneous Reproductive Outcomes After Oral Inositol Supplementation in Infertile Polycystic Ovarian Syndrome Women	2018						X		Trial registration. No article has been identified.
Nct. (https://www.cochranelibrary.com/central/doi/10.1002/central/CN-01951409/full)	Myo-inositol and Vitamin D3 During IVF	2019	X							Trial registration. The article "Positive effect of a new supplementation of vitamin D3 with myo-inositol, folic acid and melatonin on IVF outcomes: a prospective randomized controlled pilot study" by Espinola et al. (2021) has been identified. Includes male factor and combined male and female factor infertility.
Nct. (https://www.cochranelibrary.com/central/doi/10.1002/central/CN-01953100/full)	Vitamin D and Polycystic Ovarian Syndrome (PCOS)	2019						X		Trial registration. No article has been identified.
Nct. (https://www.cochranelibrary.com/central/doi/10.1002/central/CN-01968637/full)	Vitamin D and Pregnancy Outcome in PCOS Patients	2019						X		Trial registration. A published protocol of the study is also included in the search: "Vitamin D supplementation prior to in vitro fertilization in women with polycystic ovary syndrome: a protocol of a multicenter randomized, double-blind, placebo-controlled clinical trial" by Hu et al. (2020). No article has been identified.
Nct. (https://www.cochranelibrary.com/central/doi/10.1002/central/CN-01983287/full)	Vitamin D for Polycystic Ovary Syndrome Clomiphene Resistant Women	2019						X		Trial registration. No article has been identified.
Nct. (https://www.cochranelibrary.com/central/doi/10.1002/central/CN-02134451/full)	Influence of Probiotics on the Vaginal Microbiota	2020	X			X				Trial registration. The article "Effect of probiotics on vaginal Ureaplasma parvum in women suffering from unexplained infertility" by Schenk et al. (2021) has been identified. Includes male factor infertility; not relevant outcomes.

Nct. (https://www.cochranelibrary.com/central/doi/10.1002/central/CN-02341523/full)	Vitamin D Supplementation and Improvement of PCOS Therapy and IVF Outcomes in Infertile Saudi Women	2021						X		Trial registration. No article has been identified.
Nct. (https://www.cochranelibrary.com/central/doi/10.1002/central/CN-02420480/full)	NAC Effect on Infertile Women With Endometrioma	2022						X		Trial registration. No article has been identified.
Nct. (https://www.cochranelibrary.com/central/doi/10.1002/central/CN-02535820/full)	The Effects of Acetyl L--Carnitine and Myo/Chiro-Inositol on Improving Ovulation, Pregnancy Rate, Ovarian Function and Perceived Stress Response in Patients With PCOS	2023			X					Trial registration. The article "Comparative efficacy of combined myo-inotisol and D-chiro inositol versus metformin across PCOS Phenotypes: enhancing ovarian function, ovulation, and stress response in a prospective clinical trial" by Gul et al. (2025) has been identified. The comparator group receives metformin.
Ogawa, S., Ota, K., Takahashi, T., Yoshida, H.	Impact of Homocysteine as a Preconceptional Screening Factor for In Vitro Fertilization and Prevention of Miscarriage with Folic Acid Supplementation following Frozen-Thawed Embryo Transfer: A Hospital-Based Retrospective Cohort Study	2023	X							Includes male factor infertility.
Pacchiarotti A, Carlomagno G, Antonini G, Pacchiarotti A.	Effect of myo-inositol and melatonin versus myo-inositol, in a randomized controlled trial, for improving in vitro fertilization of patients with polycystic ovarian syndrome	2016							X	The publication has been retracted by the journal.
Pactr. (https://www.cochranelibrary.com/central/doi/10.1002/central/CN-01950352/full)	Could L-carnitine help poor responders to ovarian stimulation?	2019						X		Trial registration. No article has been identified.
Paffoni, A., Somigliana, E., Sarais, V., Ferrari, S., Reschini, M., Makieva, S., Papaleo, E., Viganò, P.	Effect of vitamin D supplementation on assisted reproduction technology (ART) outcomes and underlying biological mechanisms: protocol of a randomized clinical controlled trial. The "supplementation of vitamin D and reproductive outcome" (SUNDRO) study	2019	X							Protocol. The article "Single oral dose of vitamin D3 supplementation prior to in vitro fertilization and embryo transfer in normal weight women: the SUNDRO randomized controlled trial" by Somigliana et al. (2021) has been

										identified. Includes male factor and mixed male and female factor infertility.
Papaleo, E., Unfer, V., Baillargeon, J. P., De Santis, L., Fusi, F., Brigante, C., Marelli, G., Cino, I., Redaelli, A., Ferrari, A.	Myo-inositol in patients with polycystic ovary syndrome: A novel method for ovulation induction	2007					X			Not an RCT study.
Papaleo, E., Unfer, V., Baillargeon, J. P., Fusi, F., Occhi, F., De Santis, L.	Myo-inositol may improve oocyte quality in intracytoplasmic sperm injection cycles. A prospective, controlled, randomized trial	2009	X							The ICSI procedure was used based on male partner's semen sample; includes male factor infertility.
Pazhohan, A., Danaei-Mehrabad, S., Mohamad-Rezaeii, Z., Amidi, F., Khodarahmian, M., Shabani Nashtaei, M., Sobhani, A., Farajzadeh, M. A.	The modulating effects of vitamin D on the activity of β-catenin in the endometrium of women with endometriosis: a randomized exploratory trial	2021				X				Not relevant outcomes.
Prabhakar, P., Mahey, R., Gupta, M., Khadgawat, R., Kachhawa, G., Sharma, J. B., Vanamail, P., Kumari, R., Bhatla, N.	Impact of myoinositol with metformin and myoinositol alone in infertile PCOS women undergoing ovulation induction cycles - randomized controlled trial	2021	X							Includes mild male factor infertility.
Pritchard, N., Healey, M., Sorby, K., Sivapalan, S., Osianlis, T., Jatkar, S., Robinson, K., Sultana, F., Burmeister, L., Kaul, V., Vollenhoven, B.	A case control study of melatonin with or without coenzyme Q10 in improving oocyte quality and outcomes in in vitro fertilization	2015					X			Retrospective cohort study, not an RCT.
Qi, S., Liang, Q., Yang, L., Zhou, X., Chen, K., Wen, J.	Effect of Coenzyme Q10 and transcutaneous electrical acupoint stimulation in assisted reproductive technology: a retrospective controlled study	2022	X				X			Retrospective study; includes male factor infertility.
Rasheedy, R., Sammour, H., Elkholy, A., Salim, Y.	The efficacy of vitamin D combined with clomiphene citrate in ovulation induction in overweight women with polycystic ovary syndrome: a double blind, randomized clinical trial	2020							X	The journal has issued an expression of concern.
Regidor, P. A., Schindler, A. E., Lesoine, B., Druckman, R.	Management of women with PCOS using myo-inositol and folic acid. New clinical data and review of the literature	2018				X	X			Not relevant outcomes; not an RCT study

Reschini, M., Sarais, V., Ferrari, S., Cermisoni, G. C., Paffoni, A., Signorelli, S., Papaleo, E., Somigliana, E., Vigano, P.	Vitamin D supplementation prior to initiate IVF: a randomized controlled study	2020	X							Is an abstract. The article "Single oral dose of vitamin D3 supplementation prior to in vitro fertilization and embryo transfer in normal weight women: the SUNDRO randomized controlled trial" by Somigliana et al. (2021) has been identified. Includes male factor and mixed male and female factor infertility.
Ruder, E. H., Hartman, T. J., Reindollar, R. H., Goldman, M. B.	Female dietary antioxidant intake and time to pregnancy among couples treated for unexplained infertility	2014					X		X	Secondary analyses of an RCT study where two different ART treatment paradigms were compared. Information on dietary supplement use was obtained from a food frequency questionnaire.
Sen Sharma, D.	Co-enzyme Q10-A mitochondrial antioxidant -a new hope for success in infertility in clomiphene-citrate-resistant polycystic ovary syndrome	2017						X		Abstract. No article has been identified.
Sheida, A., Davar, R., Tabibnejad, N., & Eftekhar, M.	The effect of adding L-Carnitine to the GnRH-antagonist protocol on assisted reproductive technology outcome in women with polycystic ovarian syndrome: a randomized clinical trial	2023	X							Identified from hand-search of included studies. The article only excludes women with male partners with azoospermia; includes male factor infertility.
So, S., Yamaguchi, W., Murabayashi, N., Miyano, N., Tawara, F., Kanayama, N.	Beneficial effect of l-arginine in women using assisted reproductive technologies: a small-scale randomized controlled trial	2020	X							Includes male factor infertility.
Tamura, H., Takasaki, A., Miwa, I., Taniguchi, K., Maekawa, R., Asada, H., Taketani, T., Matsuoka, A., Yamagata, Y., Shimamura, K., Morioka, H., Ishikawa, H., Reiter, R. J., Sugino, N.	Oxidative stress impairs oocyte quality and melatonin protects oocytes from free radical damage and improves fertilization rate	2008					X			Not an RCT study.
Teimouri, B., Mollashahi, S., Paracheh, M., Farzaneh, F.	Comparison of the effect of letrozole alone with letrozole plus n-acetylcysteine on pregnancy rate in patients with polycystic ovarian syndrome: a randomized clinical trial	2021	X							Is an abstract. The article "Comparison of the Effect of Letrozole Alone With Letrozole Plus N-Acetylcysteine on Pregnancy Rate in Patients with Polycystic Ovarian Syndrome: A Randomized Clinical Trial" By Teimouri et al. (2021) has been identified. The article only

										excludes moderate and severe oligozoospermia, asthenozoospermia and teratozoospermia, however not mild cases of male factor infertility.
Torshin, I. Yu, Gromov, A. N., Gromova, O. A.	CHEMOMICROBIOMIC ANALYSIS OF MYOINOSITOL, D-CHIROINOSITOL, FOLIC ACID AND PYROGLUTAMATE ANION IN THE NUTRITIONAL SUPPORT OF FEMALE REPRODUCTIVE SYSTEM	2023							X	Article not written in the Latin alphabet.
Urman, B., Oktem, O.	Food and drug supplements to improve fertility outcomes	2014					X			Review article, not an RCT.
Vakhlova, O. S., Oboskalova, T. A., Kvashnina, E. V., Mukhlynina, E. A.	CLINICAL EFFICACY OF IN VITRO FERTILIZATION IN PATIENTS TREATED WITH MELATONIN DURING PRECONCEPTION CARE	2022							X	Article not written in the Latin alphabet.
Vartanyan, E. V., Tsaturova, K. A., Devyatova, E. A., Mikhaylyukova, A. S., Levin, V. A., Petuhova, N. L., Markin, A. V., Steptsova, E. M.	Improvement in quality of oocytes in polycystic ovarian syndrome in programs of in vitro fertilization	2017			X		X			Observational study design, not an RCT; no comparator group received no treatment/placebo.
Westphal, L. M., Polan, M. L., Trant, A. S.	Double-blind, placebo-controlled study of Fertilityblend: a nutritional supplement for improving fertility in women	2006	X							The article includes women who had tried to conceive for 6-36 months and thereby does not adhere to the definition of infertility.
Westphal, L. M., Polan, M. L., Trant, A. S., Mooney, S. B.	A nutritional supplement for improving fertility in women: a pilot study	2004	X							The article includes women who had tried to conceive for 6-36 months; does not adhere to the definition of infertility.
Wu, Y., Xue, H., Lu, Y.	Effects of vitamin D supplementation combined with metformin on pregnancy outcome, insulin resistance and serum oxidative stress indicators in obese polycystic ovary syndrome infertility	2024							X	Article not written in the Latin alphabet.
Xu, Y., Nisenblat, V., Lu, C., Li, R., Qiao, J., Zhen, X., Wang, S.	Pretreatment with coenzyme Q10 improves ovarian response and embryo quality in low-prognosis young women with decreased ovarian reserve: A randomized controlled trial	2018	X							Includes male factor infertility.
Zhao, J., Shan, L. I. U., Wang, Y., Wang, P., Danni, Q. U.,	Vitamin D improves in-vitro fertilization outcomes in infertile women with polycystic ovary syndrome and insulin resistance	2019					X			Not an RCT study.

Minghui, L. I. U., Wei, M. A., Li, Y.										
Zhao, W., Liu, K., Zhang, Y., Sun, P., Zeringue, E., Meng, L., Ma, H.	The efficacy of orally administered L-carnitine in alleviating ovarian dysfunctions has laid the foundation for targeted in vivo use: a study employing self-control and propensity score matching	2024	X					X		The study only excludes women with partners having azoospermia; includes male factor infertility. Propensity score matching was performed post-randomization.

Table S4. Author contacts, responses and decisions regarding inclusion of studies and data extraction.

**Article**	**Author contact**	**Response/decision**
Agrawal (2012)	Author was contacted on 9 April 2025 and on 29 April 2025 (rinaagrawal@aol.com) to request information on whether the study was indeed blinded, as it is mentioned in the results section that one woman discontinued the study because she wished to receive the intervention instead of the comparator treatment.	Author replied on 30 April 2025:<br><br>*"It was a blinded study. When patient was informed that they may get placebo vs micronutrient, they opted out of study and said they wanted to have micronutrient only."*
Alalfy (2022)	Author was contacted on 9 April 2025 and on 29 April 2025 (mahmoudalalfy@ymail.com) to request clinical pregnancy estimates as a total percentage of all clinical pregnancies in each group as it is stated that the results are reported as per treatment cycle. Further, endometrial thickness estimates were requested based on the first treatment cycle instead of an average from all three treatment cycles.	No response. Information on clinical pregnancy and endometrial thickness could not be used for quantitative synthesis.
Artini (2013)	Author was contacted on 24 April 2025 and on 14 May 2025 (paolo.artini@med.unipi.it) to request information on the definition of the reported delivery rate in terms of whether it included only live births or both live births and stillbirths.	No response. Information on delivery rate was not used.
Battaglia (2002)	Author was contacted on 9 April 2025 (battagli@med.unibo.it). Email could not be delivered to the email address and a new email was sent on April 14 2025 and on 5 May 2025 (cesare.battaglia@unibo.it). Author was contacted to request the number of live births in each treatment arm.	No response. Information on live births could not be used for quantitative synthesis.
Eryilmaz (2011)	Author was contacted on 9 April 2025 (drozlemgun@yahoo.com). Email could not be delivered to the email address and a new email was sent on April 9 2025 and on 29 April 2025 (sudesarikaya@hotmail.com). Author was contacted to request the number of clinical pregnancies in each treatment arm.	No response. Information on clinical pregnancy could not be used.
Espino (2019)	Author was contacted on 9 April 2025 and on 29 April 2025 (jespino@unex.es) to request the number of live births in each treatment arm.	No response. Information on live birth could not be used.
Fang (2024)	Author was contacted on 9 April 2025 and on 29 April 2025 (liyanhui251@163.com) to request estimates on endometrial thickness only based on the first treatment cycle for all treatment arms. Further, the cumulative biochemical pregnancy rate per patient was requested.	No response. Information on endometrial thickness and biochemical pregnancy could not be used for qualitative synthesis.
Fatemi (2017)	Author was contacted on 9 April 2025 (l.giahi@avicenna.ac.ir). Email could not be delivered to the email address and a new email was sent on April 9 2025 and on 29 April 2025 (dr.afsanehmohammadzadeh@yahoo.com). Author was contacted to request the number of biochemical and clinical pregnancies in each treatment arm.	No response. Information on biochemical and clinical pregnancies could not be used for quantitative synthesis.
Maged (2015)	Author was contacted on 9 April 2025 and on 29 April 2025 (prof.ahmedmaged@gmail.com). Author was	No response. Information on endometrial thickness and clinical pregnancy could not

	contacted to obtain information on whether participants were randomized prior to each treatment cycle and if so, to request estimates on clinical pregnancy and endometrial thickness only based on the first treatment cycle for all treatment arms.	be used for quantitative synthesis. Study was later retracted by the journal and therefore not included in the study.
Nasr (2010)	Author was contacted on 9 April 2025 (a_nasr02@lycos.com). Email could not be delivered to the email address and a new email was sent on 9 April 2025 and on 29 April 2025 (anasr@aun.edu.eg) to request estimates on endometrial thickness only based on the first treatment cycle for all treatment arms.	No response. Information on endometrial thickness could not be used for quantitative synthesis. Study was later retracted by the journal and therefore not included in the study.
Pilehvari (2023)	Author was contacted on 1 April 2025 and on 29 April 2025 (rozhinaghorbani@yahoo.com) to request information on whether both treatment arms received folic acid.	No response. The study was excluded as information needed to determine eligibility could not be obtained.
Rajan (2024)	Author was contacted on 9 April 2025 and on 29 April 2025 (sujindra@rediffmail.com) to request endometrial thickness as continuous estimates instead of dichotomous estimates. Further, the number of miscarriages in each treatment arm was requested.	No response. Information on endometrial thickness and miscarriage could not be used for quantitative synthesis.
Trop-Steinberg (2023)	Author was contacted on 9 April 2025 and on 29 April 2025 (galm@szmc.org.il) to request estimates on endometrial thickness only based on the first treatment cycle for all treatment arms.	No response. Information on endometrial thickness could not be used for quantitative synthesis.

Table S5. Characteristics of included studies

Author (year) Country	Study design and blinding	Infertility diagnosis and participant ages	Duration of treatment	Intervention (n, allocated)	Comparator (n, allocated)	Fertility medications and treatment received	Outcome measures	Conclusion
Abedi (2019)<br><br>Iran	Parallel<br><br>Double-blinded	Infertile women with vitamin D insufficiency (<30ng/mL).<br><br>18-38 years	Intervention or placebo was administered weekly from the second day of the last cycle prior to the ICSI cycle and until hCG administration. Study duration was one cycle.<br><br>Treatment duration was 6 weeks.	50,000 IU vitamin D once weekly (n=54)	Placebo once weekly (n=54)	*Medications*<br>- FSH<br>- hMG<br>- GnRH antagonist<br>- hCG<br><br>*Fertility treatment*<br>- ICSI	- Biochemical pregnancy<br>- Clinical pregnancy	Vitamin D improved biochemical pregnancy rate and clinical pregnancy rate compared to placebo.
Agrawal (2012)<br><br>United Kingdom	Parallel<br><br>Triple-blinded	Women with anovulatory infertility or at least 12 months of unexplained infertility.<br><br>19-40 years	Intervention or placebo was administered from 4 weeks prior to CC or HMG administration. Study duration was up to 3 cycles.<br><br>Minimum treatment duration was approximately 8 weeks.	Multiple micronutrient supplementation containing 8 mg thiamine, 5 mg riboflavin, 20 mg niacin, 10 mg vitamin B6, 20 µg vitamin B12, 400 µg folate, 90 mg vitamin C, 500 µg vitamin A, 15 µg vitamin D, 60 mg magnesium, 14 mg iron, 15 mg zinc, 1 mg copper, 50 µg selenium, 140 µg iodine, and 30 mg vitamin E, once daily (n=30)	Placebo administered as 400 µg folic acid pr day (n=28)	*Medications*<br>- CC or HMG<br><br>*Fertility treatment*<br>- Ovulation induction	- Clinical pregnancy<br>- Miscarriage	Micronutrient supplementation improved clinical pregnancy rate compared to placebo. No difference was seen in miscarriage rate.
Alalfy (2022)<br><br>Egypt	Parallel<br><br>No blinding	Infertile women with PCOS defined by the Rotterdam criteria<br><br>20-35 years	Intervention was administered throughout the cycle. Study duration was up to 3 cycles.<br><br>Minimum treatment duration was approximately 4 weeks.	Intervention arm 1: 1200 mg N-acetyl-cysteine per day (n=36)<br><br>Intervention arm 2: 400 µg chromium picolinate per day (n=36)	No treatment (n=36)	*Medications*<br>- CC<br>- Metformin<br><br>*Fertility treatment*<br>- Ovulation induction	- Endometrial thickness<br>- Clinical pregnancy	A difference in endometrial thickness was observed between the three groups: N-acetyl-cysteine had the highest mean endometrial thickness followed by chromium picolinate and no intervention. Clinical pregnancy rate was higher

								after chromium picolinate, while no difference was seen when comparing N-acetyl-cysteine to no intervention.
Artini (2013)<br><br>Italy	Parallel<br><br>No blinding	Women with PCOS undergoing IVF.<br><br>Intervention group mean (SD): 34.9±2.1<br>Control group mean (SD): 36.2±2.3	Treatment duration was 12 weeks. Study duration was 1 cycle.	2 g myo-inositol per day (n=25)	No treatment (n=25)	*Medications*<br>- GnRH agonist<br>- FSH<br>- hCG<br>- Progesterone<br><br>*Fertility treatment*<br>- IVF/ICSI<br><br>*Other*<br>- Folic acid, 400 µg pr day	- Biochemical pregnancy<br>- Clinical pregnancy	Myo-inositol improved biochemical pregnancy rate and clinical pregnancy rate compared to no treatment.
Badihi (2025)<br><br>Iran	Factorial<br><br>No blinding	Infertile women with recurrent implantation failure<br><br>18-40 years	Vitamin D intervention was administered for 10 weeks.	Group 1: Vaginal probiotics capsules, two per day (n=28)<br><br>Group 2: 50,000 IU vitamin D per week (n=28)<br><br>Group 3: Two vaginal probiotics capsules per day and 50,000 IU vitamin D per week (n=28)	No treatment (n=28)	*Medications*<br>- None mentioned<br><br>*Fertility treatment*<br>- IVF	- Pregnancy (not defined)<br>- Live birth	Vitamin D supplementation with or without vaginal probiotics improved undefined pregnancy rate and live birth rate compared to no treatment and vaginal probiotics only.
Battaglia (1999)<br><br>Italy	Parallel<br><br>No blinding	Women with tubal infertility who had previously undergone a failed IVF attempt.<br><br>37-44 years	Intervention or placebo was administered until at least 1 follicle was >17 mm in diameter. Study duration was 1 cycle.<br><br>Treatment duration was approximately 8 weeks[1]	16 g L-arginine per day (n=17)	No treatment (n=17)	*Medications*<br>- Monophasic oral contraception<br>- GnRH agonist<br>- FSH<br>- hCG<br><br>*Fertility treatment*<br>- IVF	- Endometrial thickness<br>- Clinical pregnancy<br>- Miscarriage	No difference was seen in endometrial thickness or pregnancy outcomes following L-arginine supplementation compared to no treatment.
Battaglia (2002)<br><br>Italy	Parallel<br><br>Double-blinded	Infertile women suffering from tubal infertility.<br><br>28-37 years	Intervention or placebo was administered until at least 2 follicles were >17 mm in diameter. Study duration was 1 cycle.	16 g L-arginine per day (n=18)	Placebo (n=19)	*Medications*<br>- GnRH agonist<br>- FSH<br>- hCG<br>- Progesterone	- Endometrial thickness<br>- Clinical pregnancy<br>- Live birth	No difference was observed in terms of endometrial thickness. Clinical pregnancy rate was higher in the placebo-group. Most pregnancies

			Treatment duration was approximately 8 weeks[1]			*Fertility treatment*<br>- IVF		resulted in live birth during the study period.
Behrouzi Lak (2017)<br><br>Iran	Parallel<br><br>No blinding	Women with PCOS defined by the Rotterdam criteria who were candidates for IUI<br><br>18-38 years	Intervention or placebo was administered from the 3rd to the 7th cycle day. Study duration was 1 cycle.<br><br>Treatment duration was 5 days.	1.2 g N-acetyl-cysteine per day (n=52)	No treatment (n=54)	*Medications*<br>- CC<br>- Letrozole<br>- FSH<br>- hCG<br><br>*Fertility treatment*<br>- IUI	- Endometrial thickness<br>- Clinical pregnancy<br>- Miscarriage	No difference was seen in endometrial thickness, clinical pregnancy rate or miscarriage rate following N-acetyl-cysteine supplementation compared to no treatment.
Cicek (2012)<br><br>Turkey	Parallel<br><br>No blinding	Women with unexplained infertility.<br><br>18-38 years	Intervention was administered from day 3-5 and until hCG injection. Study duration was 1 cycle.<br><br>Treatment duration was approximately 2 weeks.	400 IU vitamin E per day (n=53)	No treatment (n=50)	*Medications*<br>- CC<br>- hCG<br>- Progesterone<br><br>*Fertility treatment*<br>- IUI	- Endometrial thickness<br>- Clinical pregnancy	Vitamin E improved endometrial thickness compared to no treatment. No difference was observed in clinical pregnancy rate.
Emekçi Özay (2017)<br><br>Turkey	Parallel<br><br>No blinding	Anovulatory women with PCOS defined by the Rotterdam criteria who failed to conceive for > 12 months.<br><br>18-35 years	Intervention was administered from 12 weeks prior to initiation of treatment and continued until pregnancy testing. Study duration was 1 cycle.<br><br>Treatment duration was approximately 16 weeks.	4 g myo-inositol (n=98)	No treatment (n=98)	*Medications*<br>- Oral progestin (prior to ovulation induction in oligo/amenorrhoeic patients)<br>- FSH<br>- hCG<br><br>*Fertility treatment*<br>- IUI<br><br>*Other*<br>- 400 µg folic acid pr day	- Endometrial thickness<br>- Clinical pregnancy<br>- Miscarriage	Myoinositol improved clinical pregnancy rate compared to no treatment. No differences were observed in terms of endometrial thickness or miscarriage rate.
Eryilmaz (2011)<br><br>Turkey	Parallel<br><br>No blinding	Women with unexplained infertility and sleep disturbances.<br><br>24-38 years	Intervention was administered from cycle day 3-5 and until hCG injection using a long stimulation protocol. The study duration was 1 cycle.<br><br>Treatment duration was approximately 8 weeks.	3 mg melatonin per day (n=30)	No treatment (n=30)	*Medications*<br>- GnRH agonist<br>- FSH<br>- hCG<br>- Progesterone<br><br>*Fertility treatment*<br>- IVF	- Endometrial thickness<br>- Clinical pregnancy	No difference was seen in endometrial thickness or clinical pregnancy rate following melatonin supplementation compared to no treatment.

Espino (2019)<br><br>Spain	Parallel<br><br>No blinding	Infertile women suffering from unexplained infertility<br><br>Intervention group 3 mean (SD): 34.73±3.03<br>Intervention group 4 mean (SD): 36.22±2.71<br>Control group (group 2) mean (SD): 35.93±3.20	Intervention was administered from the first stimulation appointment and until the day of follicular puncture. Study duration was one cycle.<br><br>Treatment duration was 40 days.	Group 3: 3 mg melatonin per day (n=10)<br><br>Group 4: 6 mg melatonin per day (n=10)	Group 2: No treatment (n=10)<br><br>Group 1 not extracted as this group included fertile women.	*Medications*<br>- GnRH antagonist<br>- FSH<br>- hMG<br>- hCG<br><br>*Fertility treatment*<br>- IVF	- Clinical pregnancy	Three of 10 women in each intervention group with melatonin experienced clinical pregnancy, while two of 10 in the control group experienced clinical pregnancy.
Fahad (2020)<br><br>Iraq	Parallel<br><br>No blinding	Infertile women with vitamin D insufficiency (<30ng/mL).<br><br>Intervention group mean (SD): 28.88 (7.59)<br>Comparator group mean (SD): 29.27 (7.49)	Intervention was administered for 6-12 weeks. Study duration was one cycle.<br><br>Minimum treatment duration was 6 weeks[2]	50,000 IU vitamin D3 oral solution once weekly (n=43)	No treatment (n=43)	*Medications*<br>- FSH<br>- CC<br>- Letrozole<br>- hCG<br>- Dydrogesterone<br><br>*Other*<br>- Folic acid<br><br>*Fertility treatment*<br>- Ovulation induction	- Biochemical pregnancy	Vitamin D improved biochemical pregnancy rate compared to no treatment.
Fang (2024)<br><br>China	Parallel<br><br>No blinding	Infertile women with PCOS according to the Rotterdam criteria suffering from anovulation or oligo-ovulation.<br><br>21-38 years	Intervention or placebo was administered from the 3rd to the 7th cycle day. Study duration was up to 3 cycles.<br><br>Minimum treatment duration was 5 days.	1.8 g N-acetyl-cysteine per day (n=115)	No treatment (n=115)	*Medications*<br>- Letrozole<br>- FSH<br>- hCG<br>- Progesterone<br><br>*Fertility treatment*<br>- Ovulation induction	- Endometrial thickness<br>- Biochemical pregnancy<br>- Clinical pregnancy<br>- Miscarriage	N-acetyl-cysteine improved biochemical pregnancy rate and clinical pregnancy rate compared to no treatment. No differences were seen in terms of endometrial thickness or miscarriage rate.

Fatemi (2017)<br><br>Iran	Parallel<br><br>Double-blinded	Infertile women with PCOS defined by the Rotterdam criteria who were candidates for ICSI.<br><br>18-38 years	Intervention or placebo was administered from 2 weeks prior to contraceptive pill intake and until hCG administration using a long stimulation protocol. Study duration was one cycle.<br><br>Treatment duration was approximately 8 weeks.	400 mg vitamin E per day and 50,000 IU vitamin D3 every second week (n=52)	Placebo (n=53)	*Medications*<br>- LD tablets<br>- GnRH agonist<br>- FSH<br>- hCG<br>- Progesterone<br><br>*Fertility treatment*<br>- ICSI	- Endometrial thickness<br>- Biochemical pregnancy<br>- Clinical pregnancy<br>- Live birth	No difference was observed in terms of endometrial thickness. Biochemical pregnancy rate and clinical pregnancy rate improved following vitamin E and vitamin D supplementation compared to placebo. More live births were observed in the intervention group compared to the placebo group.
Ghomian (2019)<br><br>Iran	Parallel<br><br>No blinding	Infertile women with PCOS defined by the Rotterdam criteria.<br><br>28.6±6.5 years	Intervention was administered from the 3rd to the 7th day of a cycle. Study duration was one cycle.<br><br>Treatment duration was 5 days.	1200 mg N-acetyl-cysteine per day (n=33)	No treatment (n=33)	*Medications*<br>- *Spontaneous* or progesterone-induced menstruation<br>- CC<br>- hCG<br><br>*Fertility treatment*<br>- Ovulation induction	- Endometrial thickness<br>- Biochemical pregnancy	No difference was seen in endometrial thickness or biochemical pregnancy rate following N-acetyl-cysteine supplementation compared to no treatment.
Gohadkar (2024)<br><br>India	Parallel<br><br>Double-blinded	Infertile women with diminished ovarian reserve, PCOS with no symptoms or medication for the last six months and one of the following conditions: type 2 diabetes, thyroid disorder or hypertension.<br><br>30-41 years	Intervention or placebo was administered from 3 months prior to the IVF protocol and until detection of a foetal heart rate on ultrasound. Study duration was one cycle.<br><br>Treatment duration was approximately 17 weeks.[3]	EQOQ® containing 250 mg Nicotinamide Mononucleotide and 0.5 mg L-Ergothioneine per day (n=54)	Placebo (n=56)	*Medications*<br>- None mentioned<br><br>*Fertility treatment*<br>- IVF	- Clinical pregnancy<br>- Live birth	EQOQ® improved clinical pregnancy rate and live birth rate compared to placebo.

Hashemzadeh Chaleshtori (2022)<br><br>Iran	Parallel<br><br>Triple-blinded	Infertile women with PCOS defined by the Rotterdam criteria and CC resistance.<br><br>20-40 years	Intervention or placebo was administered from cycle day 3 and until pregnancy testing. Study duration was one cycle.<br><br>Treatment duration was approximately 4 weeks.	3 g L-carnitine per day (n=74)	Placebo (n=74)	*Medications*<br>- CC<br>- hCG<br><br>*Fertility treatment*<br>- Ovulation induction	- Endometrial thickness<br>- Biochemical pregnancy<br>- Clinical pregnancy	L-carnitine improved endometrial thickness, biochemical pregnancy rate and clinical pregnancy rate compared to placebo.
Henmi (2003)<br><br>Japan	Parallel<br><br>No blinding	Infertile women with luteal phase defects.<br><br>Intervention group mean (SE): 35.4 (0.4)<br>Comparator group mean (SE): 34.1 (0.6)	Intervention was administered from the first day of the cycle and until a urinary pregnancy test was positive. The study duration was up to 6 cycles.<br><br>Minimum treatment duration was approximately 4 weeks.	750 mg vitamin C per day (n=76)	No treatment (n=46)	No fertility treatment received.	- Biochemical pregnancy<br>- Miscarriage	Vitamin C supplementation improved biochemical pregnancy rate compared to no treatment. No difference was seen in terms of miscarriage rate.
Jahromi (2017)<br><br>Iran	Parallel<br><br>Double-blinded	Infertile women with diminished ovarian reserve.<br><br>22-42 years	Intervention or placebo was administered from the 5th day in a cycle and until ovum pickup in the subsequent cycle using a long stimulation protocol. Study duration was one cycle.<br><br>Treatment duration was approximately 8 weeks.	3mg melatonin per day (n=40)	Placebo (n=40)	*Medications*<br>- GnRH agonist<br>- FSH<br>- hCG<br>- Progesterone<br><br>*Fertility treatment*<br>- IVF	- Biochemical pregnancy<br>- Clinical pregnancy<br>- Miscarriage	No difference was seen in biochemical pregnancy rate, clinical pregnancy rate or miscarriage rate after melatonin supplementation compared to placebo.
Kamrani (2025)<br><br>Iran	Parallel<br><br>Double-blinded	Infertile women with recurrent implantation failure<br><br>22-43 years	Treatment duration was 6 months.	LactoFem®, two tablets per day (n=25)	Placebo (n=25)	No fertility treatment received.	- Clinical pregnancy	LactoFem® improved clinical pregnancy rate compared to placebo.

Kucuk (2023)<br><br>Turkey	Parallel<br><br>No blinding	Infertile women with PCOS defined by the Rotterdam criteria.<br><br>Intervention group median (IQR): 36 (2.5)<br>Comparator group median (IQR): 37.5 (7.75)	Intervention was administered from 2 months before ovarian stimulation using a short stimulation protocol and until oocyte pick-up. Study duration was one cycle.<br><br>Treatment duration was approximately 10 weeks.	Multiple micronutrient supplementation containing 200 mg betaine, 200 mg l-cystine, 10 mg chelated zinc, 16 mg niacin, 1.4 mg pyridoxine, 1.4 mg riboflavin, 400 µg 5-methyl-tetrahydrofolate and 2.5 µg methylcobalamin (n=24)	No treatment (n=24)	*Medications*<br>- FSH<br>- GnRH antagonist<br>- hCG<br>- Progesterone<br><br>*Fertility treatment*<br>- ICSI	- Biochemical pregnancy<br>- Clinical pregnancy	No difference was seen in biochemical pregnancy rate or clinical pregnancy rate after multiple micronutrient supplementation compared to no treatment.
Mier-Cabrera (2008)<br><br>Mexico	Parallel<br><br>Double-blinded	Infertile women with stage I or II endometriosis according to the revised criteria of the American Society for Reproductive Medicine.<br><br>25-35 years	Treatment duration was 6 months.	343 mg vitamin C and 84 mg vitamin E administered in a tasting bar, one per day (n=18)	Placebo, administered as a tasting bar without vitamins, one per day (n=18)	No fertility treatment received.	- Pregnancy (not defined)	No difference was seen in undefined pregnancy rate following Vitamin E or vitamin C supplementation compared to placebo.
Mohammadi (2021)<br><br>Iran	Parallel<br><br>Triple-blinded	Infertile women with poor ovarian response<br><br>20-43 years	Intervention or placebo was administered for 12 weeks prior to ICSI. Study duration was one cycle.<br><br>Treatment duration was 12 weeks.	4 g myo-inositol and 400 µg folic acid per day (n=38)	Placebo administered as 400 µg folic acid per day (n=38)	*Medications*<br>- FSH<br>- LH<br>- GnRH antagonist<br>- hCG<br><br>*Fertility treatment*<br>- ICSI	- Biochemical pregnancy<br>- Clinical pregnancy<br>- Miscarriage	No difference was seen in biochemical pregnancy rate, clinical pregnancy rate or miscarriage rate following myo-inositol supplementation compared to placebo.
Morsy (2020)<br><br>Egypt	Parallel<br><br>No blinding	Infertile women with PCOS defined by the Rotterdam criteria and clomiphene resistance.<br><br>Intervention group mean (SD): 24.9±3.78<br>Comparator	Intervention was administered from four weeks prior to CC administration and onwards. Study duration was up to 3 cycles.<br><br>Minimum treatment duration was approximately 8 weeks.	1500 IU vitamin E per day (n=30)	No treatment (n=30)	*Medications*<br>- Metformin<br>- Spontaneous or progesterone-induced menstruation<br>- CC<br>- hCG<br><br>*Fertility treatment*	- Endometrial thickness<br>- Biochemical pregnancy	Vitamin E improved endometrial thickness compared to no treatment. No difference was seen in terms of biochemical pregnancy rate.

		group mean (SD): 24±3.60				- Ovulation induction		
Moti (2024)<br><br>Iran	Parallel<br><br>Triple-blinded	Infertile women with PCOS defined by the Rotterdam criteria.<br><br>18-40 years	Intervention or placebo was administered for 4 weeks prior to oocyte transfer. Study duration was one cycle.<br><br>Treatment duration was 4 weeks.	300 mg vitamin B1 per day (n=35)	Placebo (n=35)	*Medications*<br>- None mentioned<br><br>*Fertility treatment*<br>- IVF	- Biochemical pregnancy	Vitamin B1 supplementation improved biochemical pregnancy rate compared to placebo.
Panti Abubakar (2018)<br><br>Nigeria	Parallel<br><br>Single-blinded	Infertile women with PCOS and anovulation.<br><br>Intervention group mean (SEM): 28.18 ±0.82<br>Placebo group mean (SEM): 28.12±0.81	Intervention or placebo was administered for up to 6 months. Study duration was up to 6 cycles.<br><br>Minimum treatment duration was approximately 4 weeks.	Vitacap® containing 5000 IU vitamin A, 5mg vitamin B1, 2 mg vitamin B6, 5 mg vitamin B12, 75 mg vitamin C, 400 IU vitamin D3, 15 mg vitamin E, 45 mg nicotinamide, 1000 µg folic acid, 50 mg ferrous fumarate, 70 mg dibasic calcium phosphate, 0.1 mg copper sulphate, 0.01 mg manganese sulphate, 50 mg zinc sulphate, 0.025 mg potassium iodide, 0.5 mg magnesium oxide (n=100)	Placebo administered as 100 mg ferrous fumarate per day (n=100)	*Medications*<br>- CC<br><br>*Fertility treatment*<br>- Ovulation induction	- Clinical pregnancy<br>- Live birth<br>- Miscarriage	The multiple micronutrient supplement improved clinical pregnancy rate and live birth rate compared to placebo. No difference was seen in terms of miscarriage rate.
Pourghasem (2019)<br><br>Iran	Parallel<br><br>Single-blinded	Infertile women with PCOS defined by the Rotterdam criteria with	Intervention or placebo was administered for 3 months. Study duration was one cycle.	Group 3: 4000 mg myo-inositol and 400 µg folic acid per day (n=62)	Placebo administered as 400 µg folic acid per day (n=62)	*Medications*<br>- Letrozole<br>- hCG<br><br>*Fertility treatment*	- Clinical pregnancy	No difference was seen in clinical pregnancy rate following myo-inositol supplementation compared to placebo.

		letrozole resistance.<br><br>15-38 years	Treatment duration was 3 months.	Group 2 received metformin and folic acid and was therefore not included in data extraction.		- Ovulation induction		
Rajan (2024)<br><br>India	Parallel<br><br>No blinding	Infertile women with PCOS according to the Rotterdam criteria<br><br>20-35 years	Intervention was administered throughout the cycle. Study duration was up to 3 cycles.<br><br>Minimum treatment duration was approximately 4 weeks.	2 g myo-inositol and 1000 IU vitamin D3 per day (n=47)	No treatment (n=47)	*Medications*<br>- hCG<br><br>*Fertility treatment*<br>- Ovulation induction	- Endometrial thickness<br>- Pregnancy (not defined)<br>- Miscarriage	Undefined pregnancy rate improved after myo-inositol and vitamin D supplementation compared to no treatment. No difference was seen in terms of endometrial thickness or miscarriage rate.
Rashidi (2009)<br><br>Iran	Parallel<br><br>No blinding	Infertile women with PCOS defined by the Rotterdam criteria.<br><br>20-40 years	Intervention was administered from cycle day 1 and continued for 3 months.<br><br>Treatment duration was 3 months.	Group 2: 1,000 mg calcium and 400 IU vitamin D per day (n=20)<br><br>Group 1 received calcium and vitamin D without metformin and was therefore not included in data extraction.	No treatment (n=20)	*Medications*<br>- Metformin<br><br>*Fertility treatment*<br>- No fertility treatment received.	- Biochemical pregnancy	No difference was seen in biochemical pregnancy rate following vitamin D or calcium supplementation compared to no treatment.
Rizk (2005)<br><br>Egypt	Parallel<br><br>Double-blinded	Infertile women with PCOS and CC-resistance<br><br>18-39 years	Intervention or placebo was administered from the 3rd to the 7th cycle day. Study duration was 1 cycle.<br><br>Treatment duration was 5 days.	1.2 g N-acetyl-cysteine per day (n=75)	Placebo (n=75)	*Medications*<br>- Spontaneous or progesterone-induced menstruation<br>- CC<br>- hCG<br><br>*Fertility treatment*<br>- Ovulation induction	- Endometrial thickness<br>- Pregnancy (not defined)<br>- Miscarriage	N-acetyl-cysteine improved undefined pregnancy rate compared to placebo. No difference was observed in terms of endometrial thickness or miscarriage.
Rizzo (2010)<br><br>Italy	Parallel<br><br>No blinding	Infertile women with low oocyte quality detected in the previous IVF cycles.<br><br>35-42 years	Intervention was administered from the day of GnRH administration using a long stimulation protocol. Study duration was 1 cycle.	6 mg melatonin per day (n=32)	no treatment (n=33)	*Medications*<br>- GnRH agonist<br>- FSH<br>- hCG<br>- Progesterone<br><br>*Fertility treatment*<br>- IVF	- Biochemical pregnancy<br>- Clinical pregnancy<br>- Miscarriage	No difference was seen in biochemical pregnancy rate, clinical pregnancy rate or miscarriage rate following melatonin in combination with myo-inositol and folic acid supplementation

			Treatment duration was approximately 7 weeks.			*Other*<br>- 4 g myo-inositol and 400 µg folic acid pr day		compared to myo-inositol and folic acid given alone.
Rostami (2023)<br><br>Iran	Parallel<br><br>Triple-blinded	Infertile women with endometriosis stage III or IV.<br><br>20-40 years	Intervention or placebo was administered for 12 weeks from day 1 of two cycles before ovarian stimulation and until oocyte pickup. Study duration was one cycle.<br><br>Treatment duration was 12 weeks.	6 mg astaxanthin per day (n=28)	Placebo (n=29)	*Medications*<br>- FSH<br>- GnRH antagonist<br>- hCG<br><br>*Fertility treatment*<br>- ICSI<br><br>*Other*<br>- Participants were instructed to take prenatal folic acid (800 µg per day)	- Biochemical pregnancy<br>- Clinical pregnancy	No difference was seen in biochemical pregnancy rate or clinical pregnancy rate following Astaxanthin supplementation compared to placebo.
Salehpour (2012)<br><br>Iran	Parallel<br><br>Double-blinded	Infertile women with PCOS defined by the Rotterdam criteria.<br><br>20-35 years	Intervention or placebo was administered from the 3rd to the 7th cycle day. Study duration was 1 cycle.<br><br>Treatment duration was 5 days.	1200 mg N-acetyl-cysteine per day (n=90)	Placebo (n=90)	*Medications*<br>- Spontaneous or progesterone-induced menstruation<br>- CC<br>- hCG<br><br>*Fertility treatment*<br>- Ovulation induction	- Endometrial thickness<br>- Pregnancy (not defined)	N-acetyl-cysteine improved endometrial thickness and undefined pregnancy rate compared to placebo.
Seyedoshohadaei (2022)<br><br>Iran	Parallel<br><br>Double-blinded	Infertile women.<br><br>20-40 years	Intervention or placebo was administered for 2 months from the 3rd day of the cycle and until the end of the 2nd month. Study duration was one cycle.<br><br>Treatment duration was 2 months.	4000 mg myo-inositol and 400 µg folic acid per day (n=36)	Placebo administered as 400 µg folic acid per day (n=34)	*Medications*<br>- Low dose oral contraceptive pills<br>- GnRH agonist<br>- FSH<br>- hCG<br>- Progesterone<br><br>*Fertility treatment*<br>- IVF/ICSI	- Clinical pregnancy<br>- Live birth<br>- Miscarriage	Myo-inositol improved clinical pregnancy rate and live birth rate compared to placebo. No difference was observed in terms of miscarriage rate.
Soufizadeh (2021)<br><br>Iran	Parallel<br><br>No blinding	Infertile women with PCOS.<br><br>20-40 years	Intervention was administered for 3 months. Study duration was op to 3 cycles.<br><br>Minimum treatment	2000 mg myo-inositol and 200 µg folic acid per day (n=35)	No treatment (n=35)	*Medications*<br>- CC<br><br>*Fertility treatment*<br>- Ovulation induction	- Clinical pregnancy<br>- Live birth<br>- Miscarriage	No difference was seen in clinical pregnancy rate, live birth rate or miscarriage rate following myo-inositol and folic acid supplementation compared to no treatment.

			duration was approximately 4 weeks.					
Trop-Steinberg (2023)<br><br>Israel	Parallel<br><br>Double-blinded	Infertile women with chronic oligo/anovulation due to PCOS.<br><br>25-38 years	Intervention or placebo was administered from the first day of the cycle and until pregnancy or study completion. Study duration was up to 2 cycles.<br><br>Minimum treatment duration was approximately 4 weeks.	1800 mg omega 3 per day containing 1080 mg EPA and 720 mg DHA (n=17)	Placebo (n=17)	*Medications*<br>- CC<br><br>*Fertility treatment*<br>- Ovulation induction	- Endometrial thickness<br>- Clinical pregnancy	No difference was seen in endometrial thickness or clinical pregnancy rate following omega-3 fatty acids supplementation compared to placebo.
Wdowiak and Filip (2020)<br><br>Poland	Parallel<br><br>Unclear blinding	Infertile women with a history of multiple follicle growth after monofollicular stimulation, issues with follicle rupture, or three unsuccessful inseminations.<br><br>20-35 years	Intervention or placebo was administered 3 months before and 3 months after oocyte pickup. Study duration was one cycle.<br><br>Treatment duration was approximately 15 weeks.	2,6 g myo-inositol, 50 mg alpha-Lactalbumin, 1 mg melatonin and 400 µg folic acid per day before oocyte pickup and 2,6 g myo-inositol, 50 mg alpha-Lact-albumin, 1 mg melatonin, 2000 IU vitamin D3 and 400 µg folic acid per day after ICSI (n=50)	Placebo administered as 400 µg folic acid per day (n=50)	*Medications*<br>- GnRH agonist<br>- FSH<br>- hCG<br><br>*Fertility treatment*<br>- ICSI	- Endometrial thickness<br>- Clinical pregnancy	Myo-inositol, vitamin D3 and melatonin improved endometrial thickness and clinical pregnancy rate compared to placebo.
Youssef (2015)<br><br>Egypt	Parallel<br><br>No blinding	Infertile women with unexplained infertility.<br><br>Intervention group mean (SD): 30.9±5.7<br>Control group mean (SD): 30.6±5.4	Intervention was administered from the beginning of the study using a long stimulation protocol. Study duration was one cycle.<br><br>Treatment duration was approximately 10 weeks. [4]	Antioxidant supplement containing 3000 IU vitamin A, 15 IU vitamin E, 90 mg vitamin C, 11 mg zinc, 45 µg molybdenum, 55 µg selenium, 10 µg biotin and 100 mg mixed bioflavonoid (n=112)	No treatment (n=106)	*Medications*<br>- GnRH agonist<br>- FSH<br>- hCG<br>- Progesterone<br><br>*Fertility treatment*<br>- IVF/ICSI<br><br>*Other*<br>- Folic acid, 2.5 mg pr day	- Clinical pregnancy<br>- Miscarriage	No difference was seen in clinical pregnancy rate or miscarriage rate following antioxidant supplementation compared to no treatment.
Zadeh Modarres (2022)<br><br>Iran	Parallel<br><br>Double-blinded	Infertile women with PCOS defined by the Rotterdam criteria.	Intervention or placebo was administered for 8 weeks. Study duration was one cycle.	200 µg selenium per day (n=20)	Placebo (n=20)	*Medications*<br>- None mentioned<br><br>*Fertility treatment*<br>- IVF	- Pregnancy (not defined)	No difference was seen in undefined pregnancy rate following selenium supplementation compared to placebo.

		18-40 years	Treatment duration was 8 weeks.					

[1]It was assumed that the intervention was administered from cycle day 1 using a long stimulation protocol. [2]The article reports different treatment durations in the abstract and the main text. Therefore, the shortest duration of treatment has been extracted. [3]As the stimulation protocol has not been described, it was assumed to be a short stimulation protocol. [4]The exact start of intervention administration has not been defined. Therefore, it has been extracted as administration from cycle day 1. CC: clomiphene citrate, DHA: docosahexaenoic acid, EPA: eicosapentaenoic acid, FSH: follicle-stimulating hormone, GnRH: gonadotropin-releasing hormone, hCG: human chorionic gonadotropin, HMG: human menopausal gonadotropin, ICSI: intracytoplasmic sperm injection, IUI: intrauterine insemination, IVF: in vitro fertilization, LH: luteinizing hormone, PCOS: polycystic ovary syndrome.

Table S6. Certainty of evidence assessment of myo-inositol compared to placebo for female infertility.

Outcome	Participants (studies)	Risk of Bias	Inconsistency	Indirectness	Imprecision	Publication bias	Overall certainty of evidence
Clinical pregnancy	220 (3 RCTs)	serious[a]	not serious	not serious	serious[b]	none	⊕⊕◯◯ Low[a,b]
Miscarriage	60 (2 RCTs)	serious[a]	not serious	not serious	serious[b]	none	⊕⊕◯◯ Low[a,b]

a. Downgraded as one or more studies had some concerns in bias arising from the randomization process as no information on allocation sequence concealment was available and some concerns in selection of the reported results as the trial registrations were registered retrospectively. b. Downgraded due to small sample sizes and a low number of total events.

Table S7. Certainty of evidence assessment of N-acetyl-cysteine compared to placebo for female infertility.

Outcome	Participants (studies)	Risk of Bias	Inconsistency	Indirectness	Imprecision	Publication bias	Overall certainty of evidence
Endometrial thickness	317 (2 RCTs)	serious[a]	not serious	not serious	serious[b]	none	⊕⊕◯◯ Low[a,b]
Undefined pregnancy	317 (2 RCTs)	serious[a]	not serious[c]	not serious	serious[d]	none	⊕⊕◯◯ Low[a,c,d]

a. Downgraded as one study had some concerns in bias arising from the randomization process as no information on allocation sequence concealment was available and some concerns in bias due to deviations from the intended intervention as intention-to-treat analysis was not used. Further, all studies had some concerns in selection of the reported results, as no protocol was available. b. Downgraded due to small sample sizes. c. Statistical heterogeneity was substantial (I^2=70%, p=0.07). However, not downgraded as the effect estimated pointed in the same direction and some of the observed heterogeneity could be explained by one study in which there were zero events in the placebo group. d. Imprecision was downgraded due to small sample sizes and a low number of total events.

Table S8. Certainty of evidence assessment of multiple substance dietary supplements compared to placebo for female infertility.

Outcome	Participants (studies)	Risk of Bias	Inconsistency	Indirectness	Imprecision	Publication bias	Overall certainty of evidence
Clinical pregnancy	358 (3 RCTs)	serious[a]	not serious[b]	not serious	serious[c]	none	⊕⊕◯◯ Low[a,b,c]
Miscarriage	258 (2 RCTs)	serious[d]	not serious[e]	not serious	serious[c]	none	⊕⊕◯◯ Low[c,d,e]

a. Risk of bias was downgraded as one study had some concerns in bias arising from the randomization process as no information on allocation sequence concealment was available. Further, all studies had some concerns in selection of the reported results as no protocol was available. b. Heterogeneity was high (I^2=80%, p=0.05), however, inconsistency was not downgraded as effect estimates had the same direction and some heterogeneity could be explained by one study with very wide confidence intervals which likely contributed to the heterogeneity. c. Imprecision was downgraded due to small sample sizes and a low number of total events. d. Risk of bias was downgraded due to both studies having some concerns in selection of the reported results as no protocols were available. e. The direction of the effect varies across studies and heterogeneity is substantial (I^2=75%, p=0.05), however inconsistency was not downgraded as confidence intervals overlap and some of the observed heterogeneity could be explained by one study in which there were zero events in the placebo group.

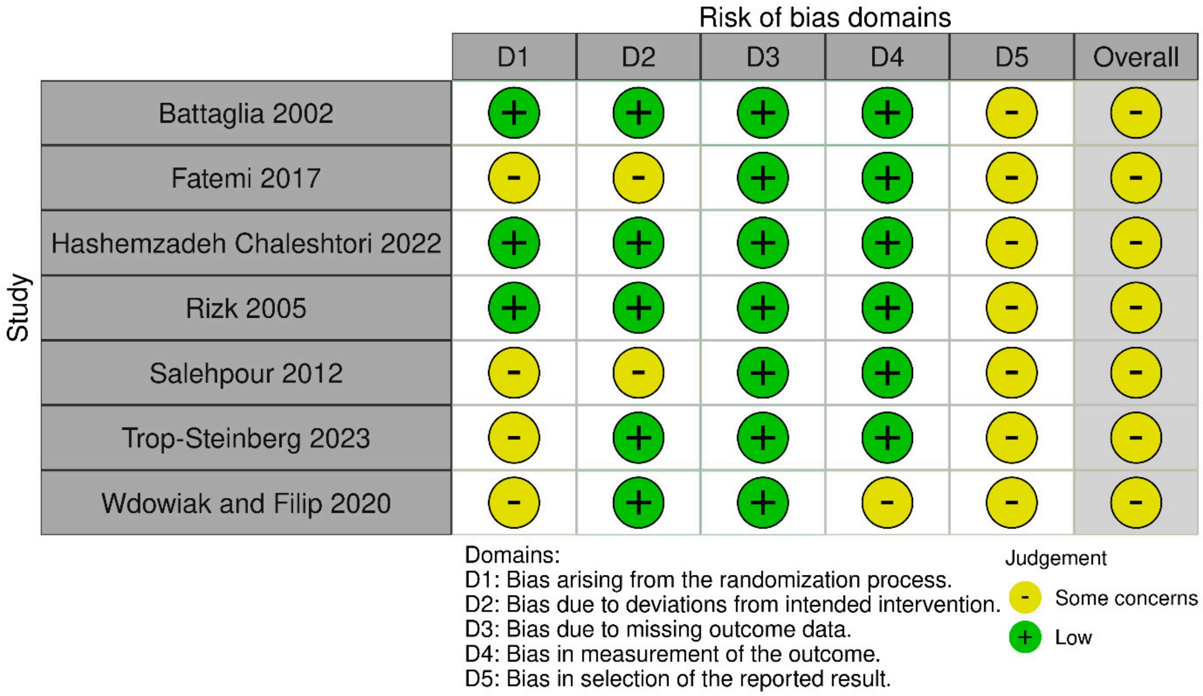

Figure S1. Traffic-light plot of risk of bias assessments on the outcome endometrial thickness in placebo-controlled studies. The plot was created using the robvis© 2025 tool.

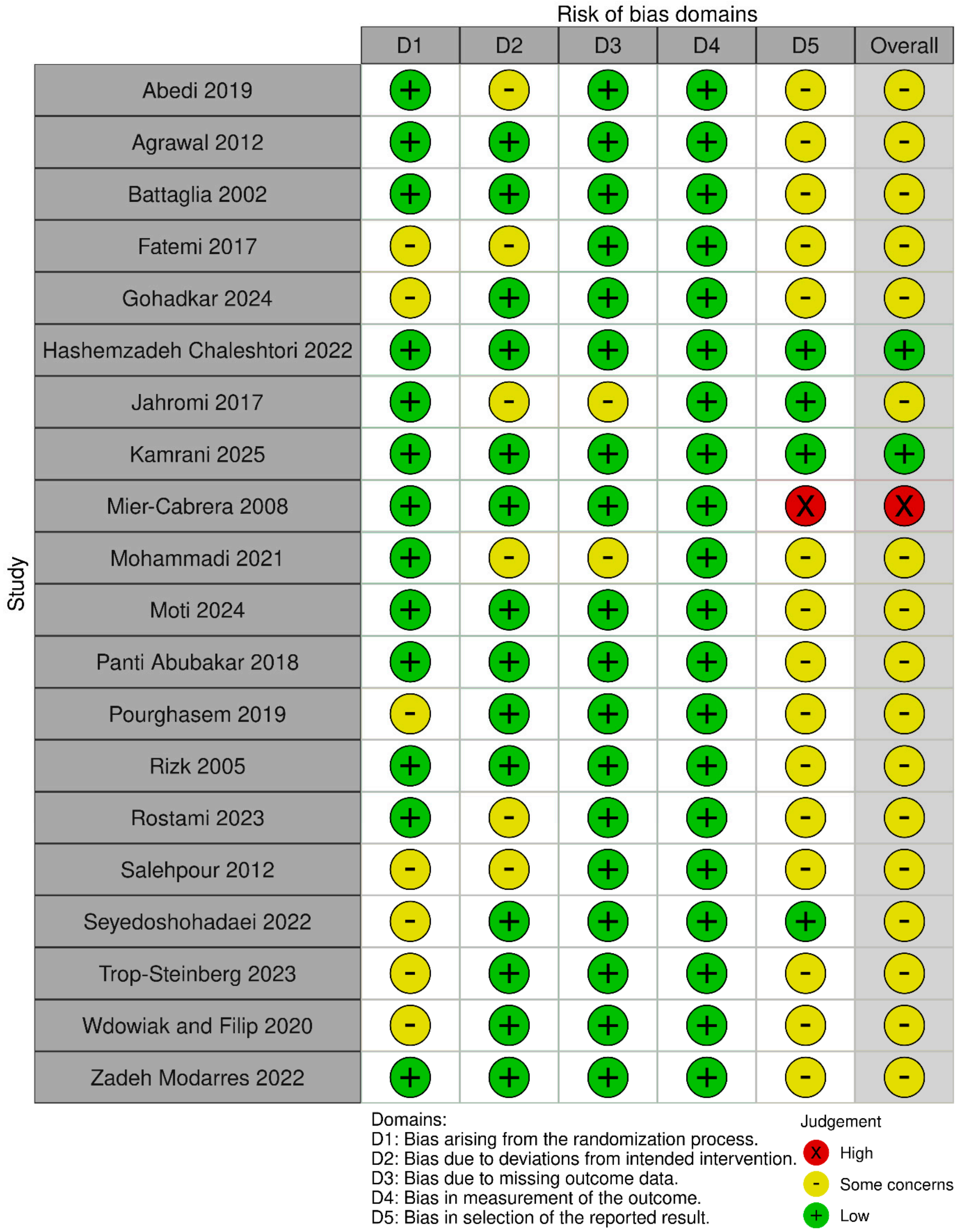

Figure S2. Traffic-light plot of risk of bias assessments on pregnancy, live birth and miscarriage outcomes in in placebo-controlled studies. The plot was created using the robvis© 2025 tool.

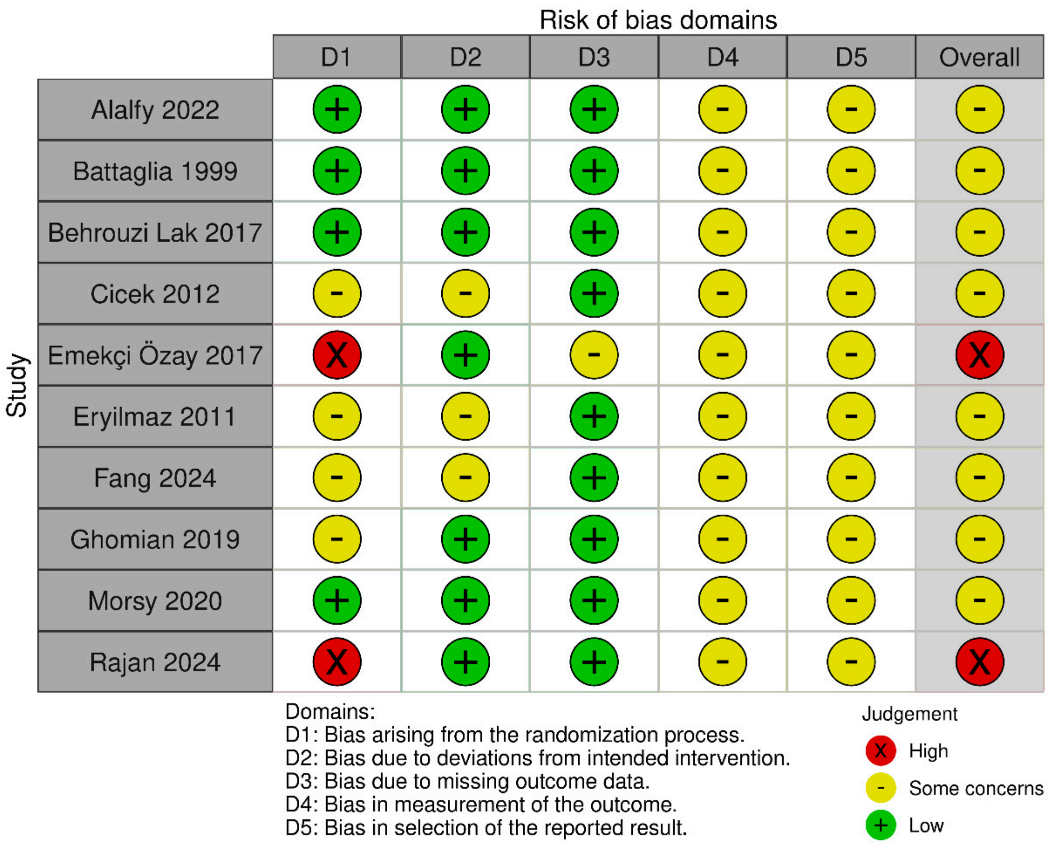

Figure S3. Traffic-light plot of risk of bias assessments on the outcome endometrial thickness in studies using no comparator treatment. The plot was created using the robvis© 2025 tool.

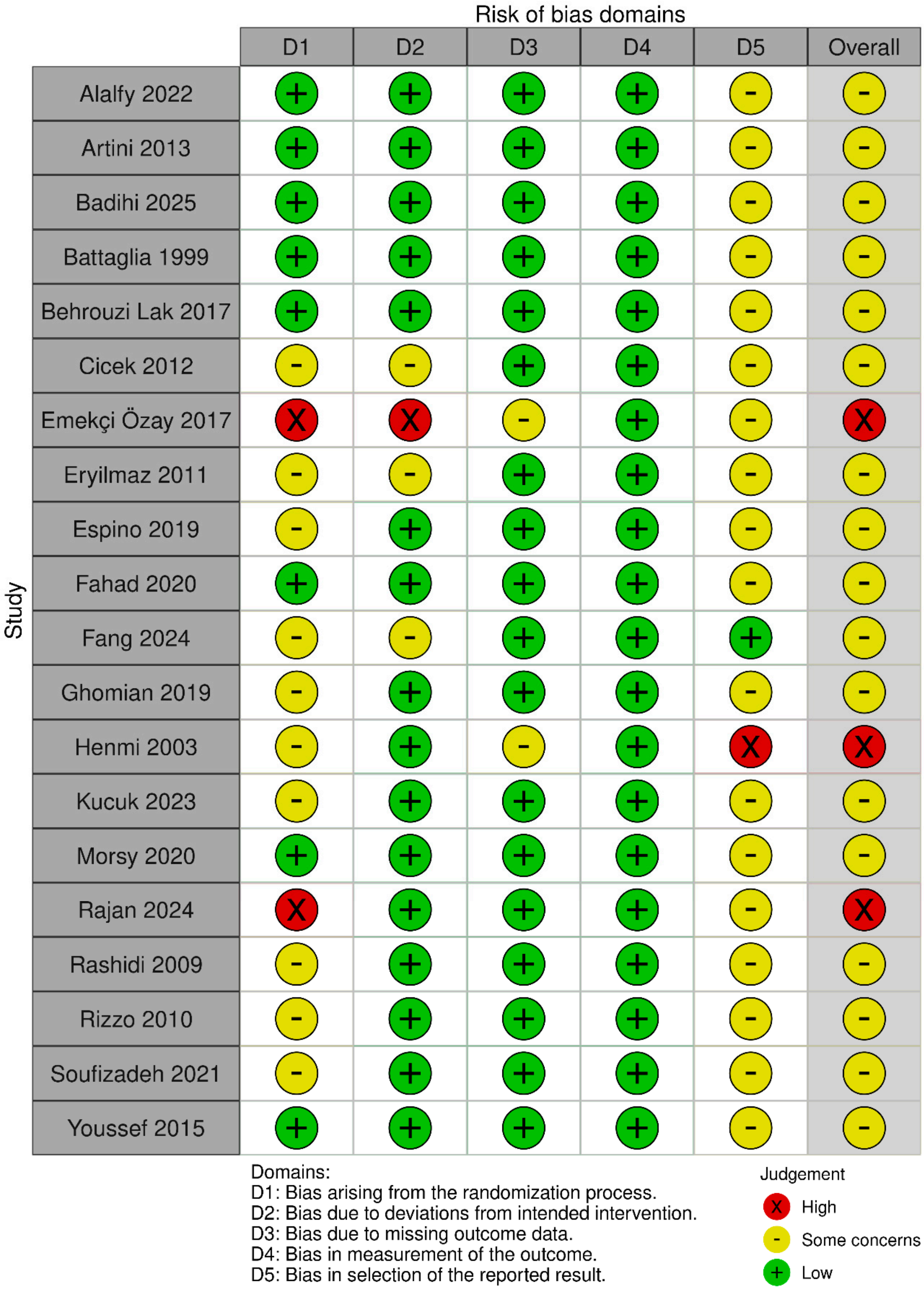

Figure S4. Traffic-light plot of risk of bias assessments on pregnancy, live birth and miscarriage outcomes in studies using no comparator treatment. The plot was created using the robvis© 2025 tool.

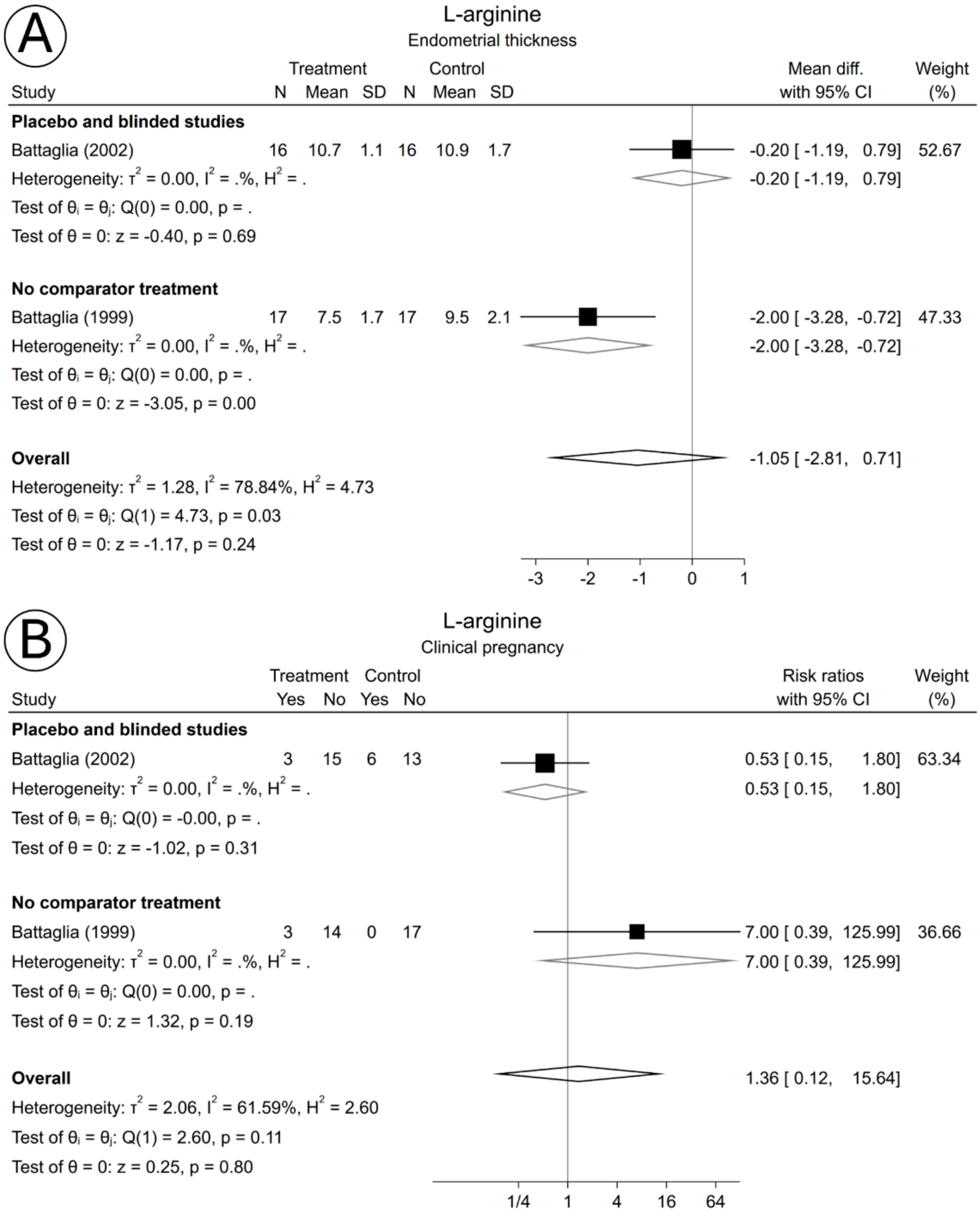

Figure S5. Forest plots of secondary analysis on the effect of L-arginine on (A) endometrial thickness and (B) clinical pregnancy.

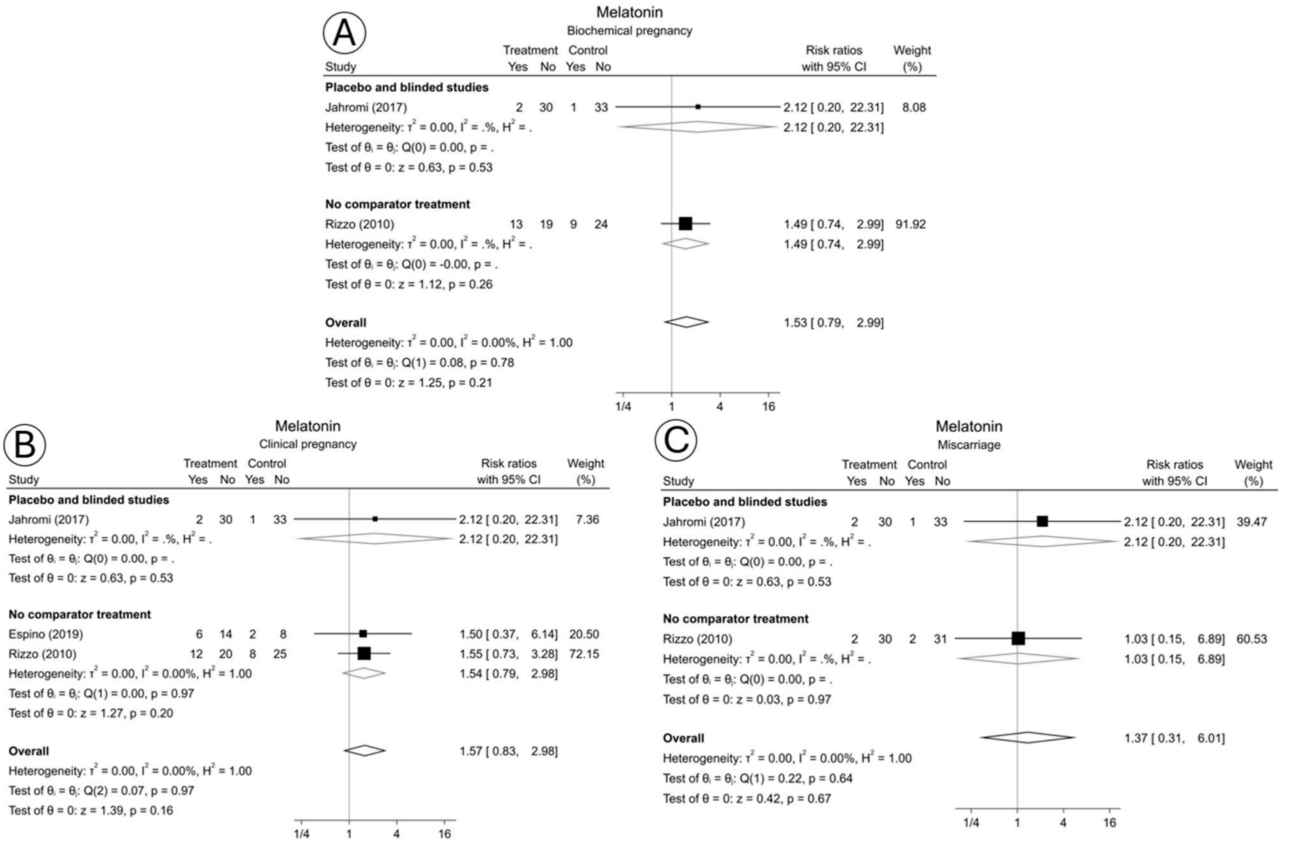

Figure S6. Forest plot of secondary analysis on the effect of melatonin on (A) biochemical pregnancy, (B) clinical pregnancy, and (C) miscarriage.

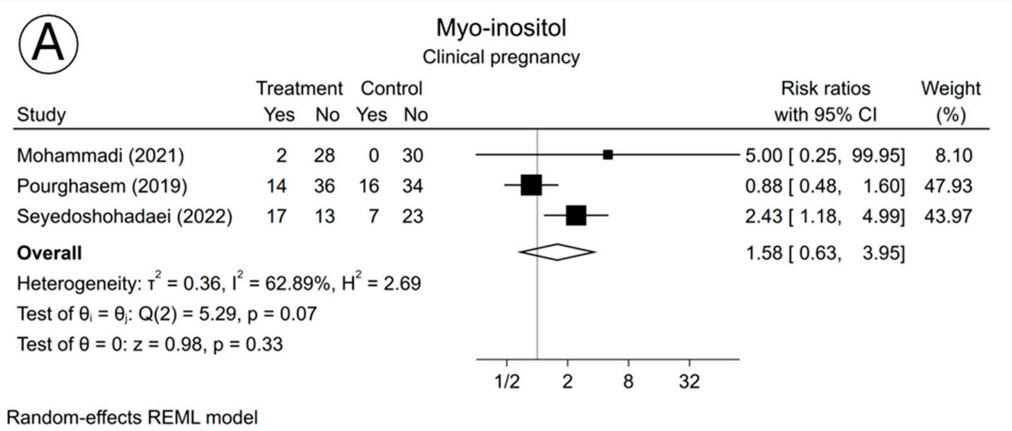

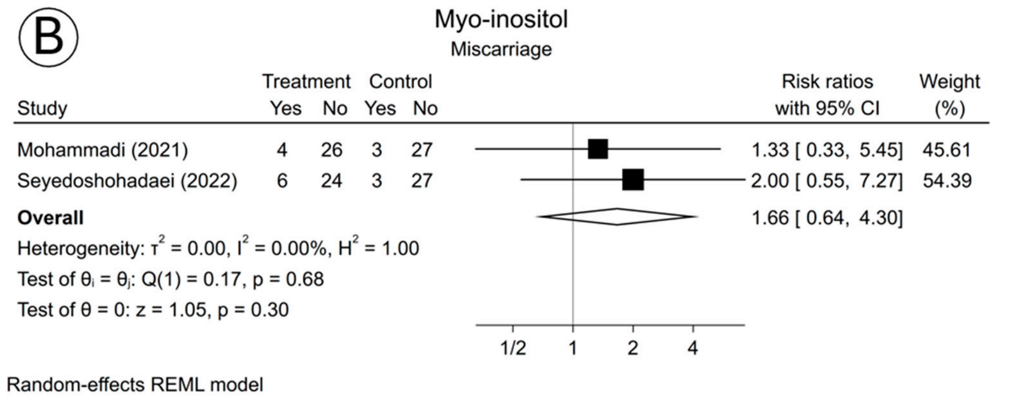

Figure S7. Forest plot of primary analysis on the effect of myo-inositol on (A) clinical pregnancy and (B) miscarriage.

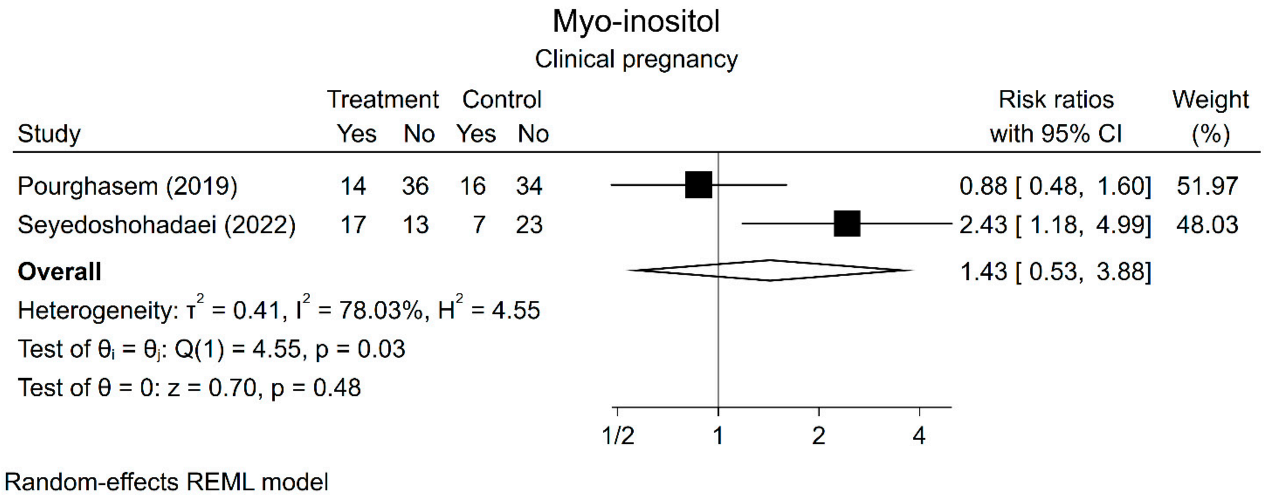

Figure S8. Forest plot of post hoc sensitivity analyses on the effect of myo-inositol on clinical pregnancy excluding zero-events studies.

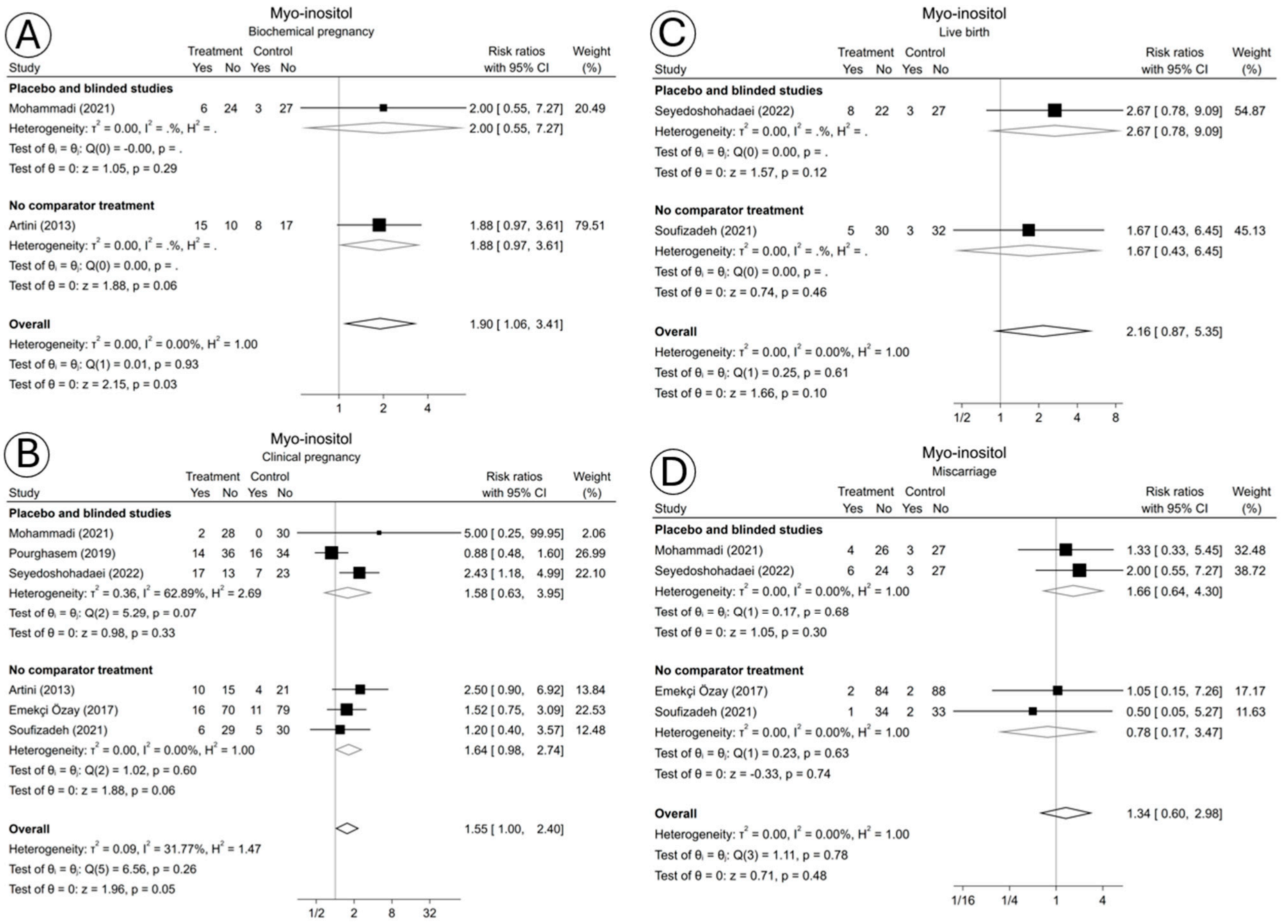

Figure S9. Forest plot of secondary analysis on the effect of myo-inositol on (A) biochemical pregnancy, (B) clinical pregnancy, (C) live birth and (D) miscarriage.

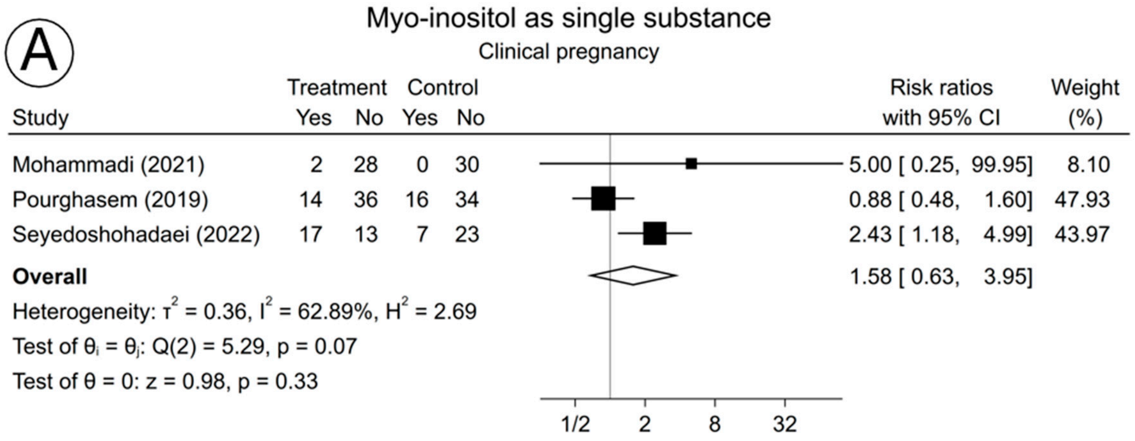

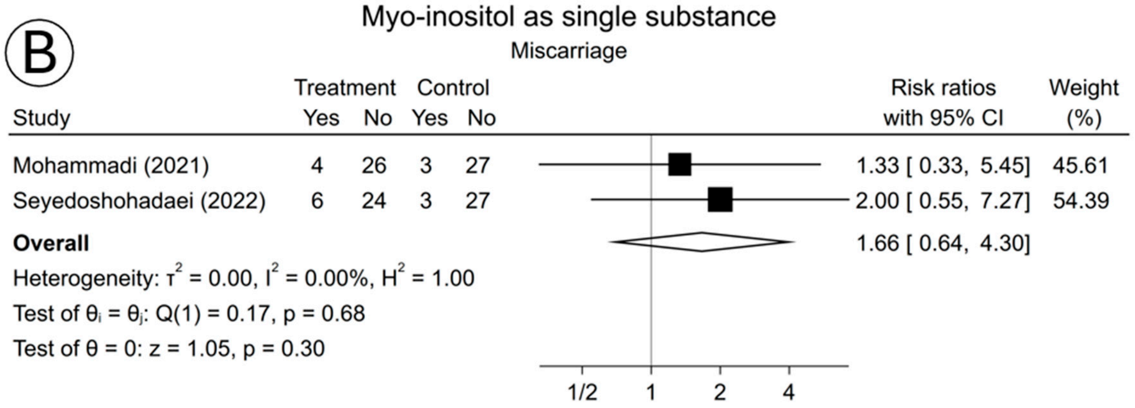

Figure S10. Forest plot of subgroup analysis on the effect of myo-inositol as a single substance on (A) clinical pregnancy and (B) miscarriage.

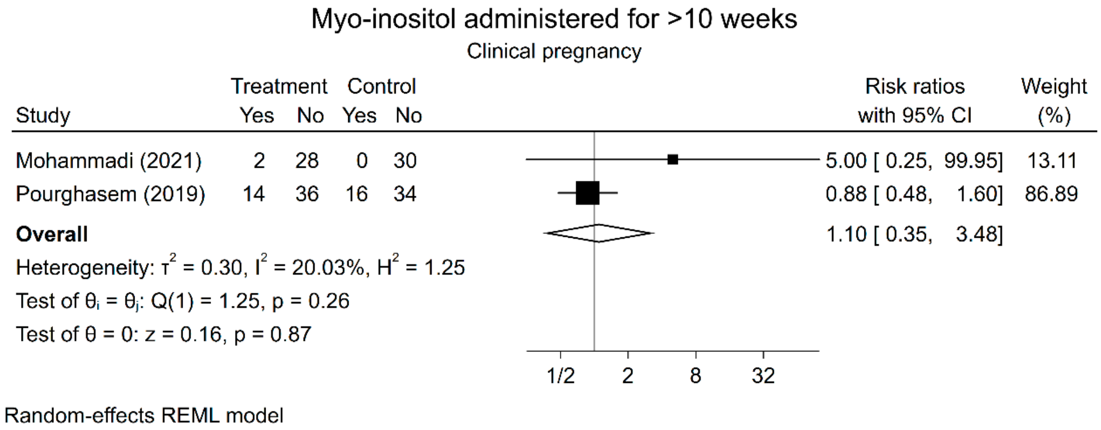

Figure S11. Forest plot of subgroup analysis on the effect of >10 weeks of myo-inositol on clinical pregnancy

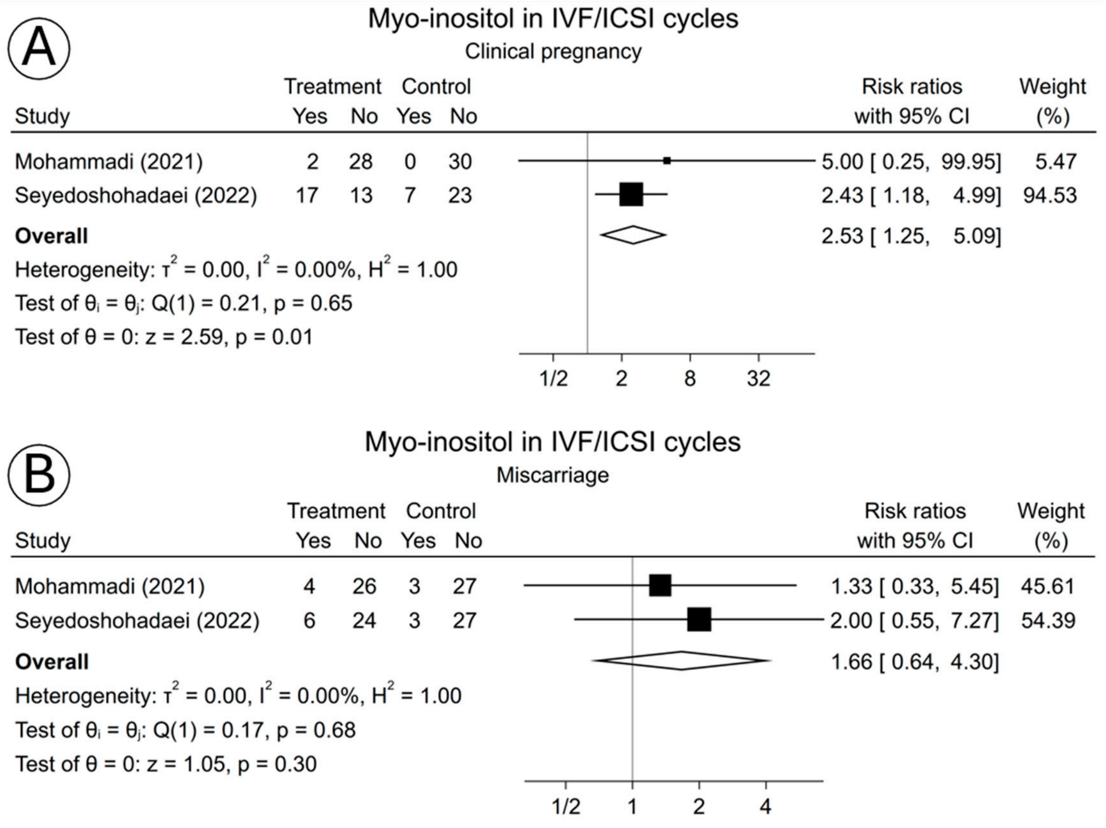

Figure S12. Forest plot of subgroup analysis on the effect of myo-inositol on (A) clinical pregnancy and (B) miscarriage in in vitro fertilization/intracytoplasmic sperm injection cycles.

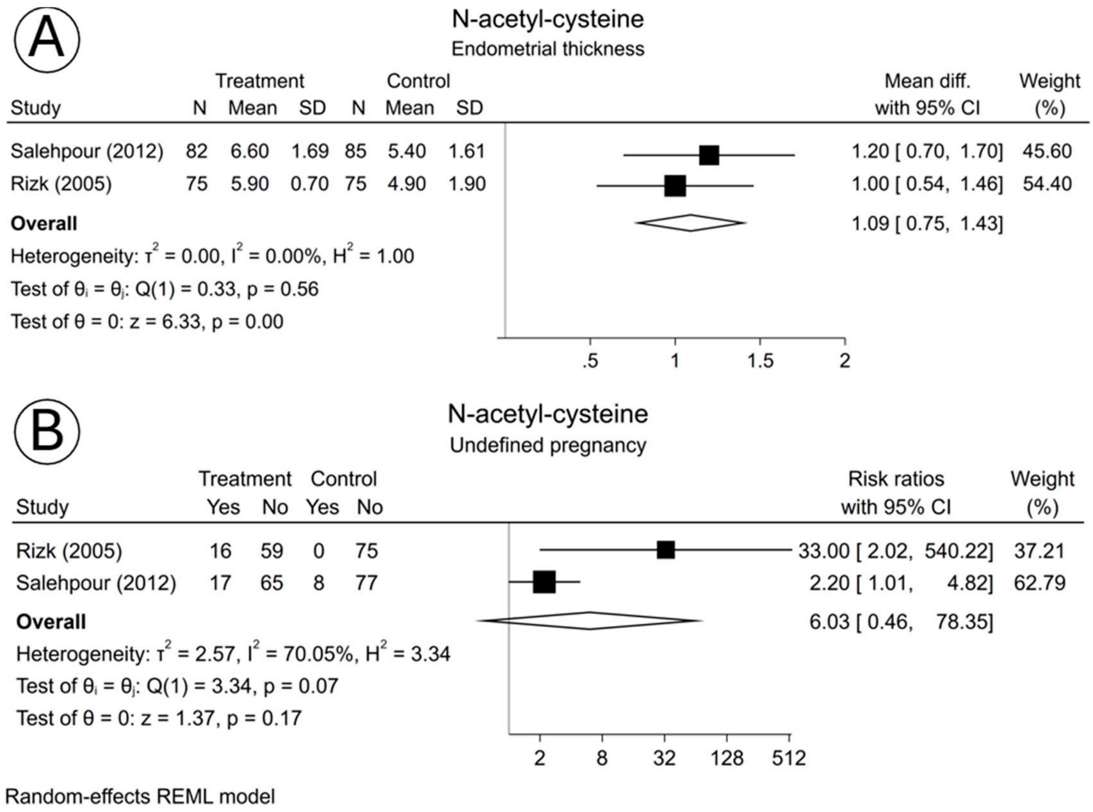

Figure S13. Forest plot of primary analysis on the effect of N-acetyl-cysteine on (A) endometrial thickness and (B) undefined pregnancy.

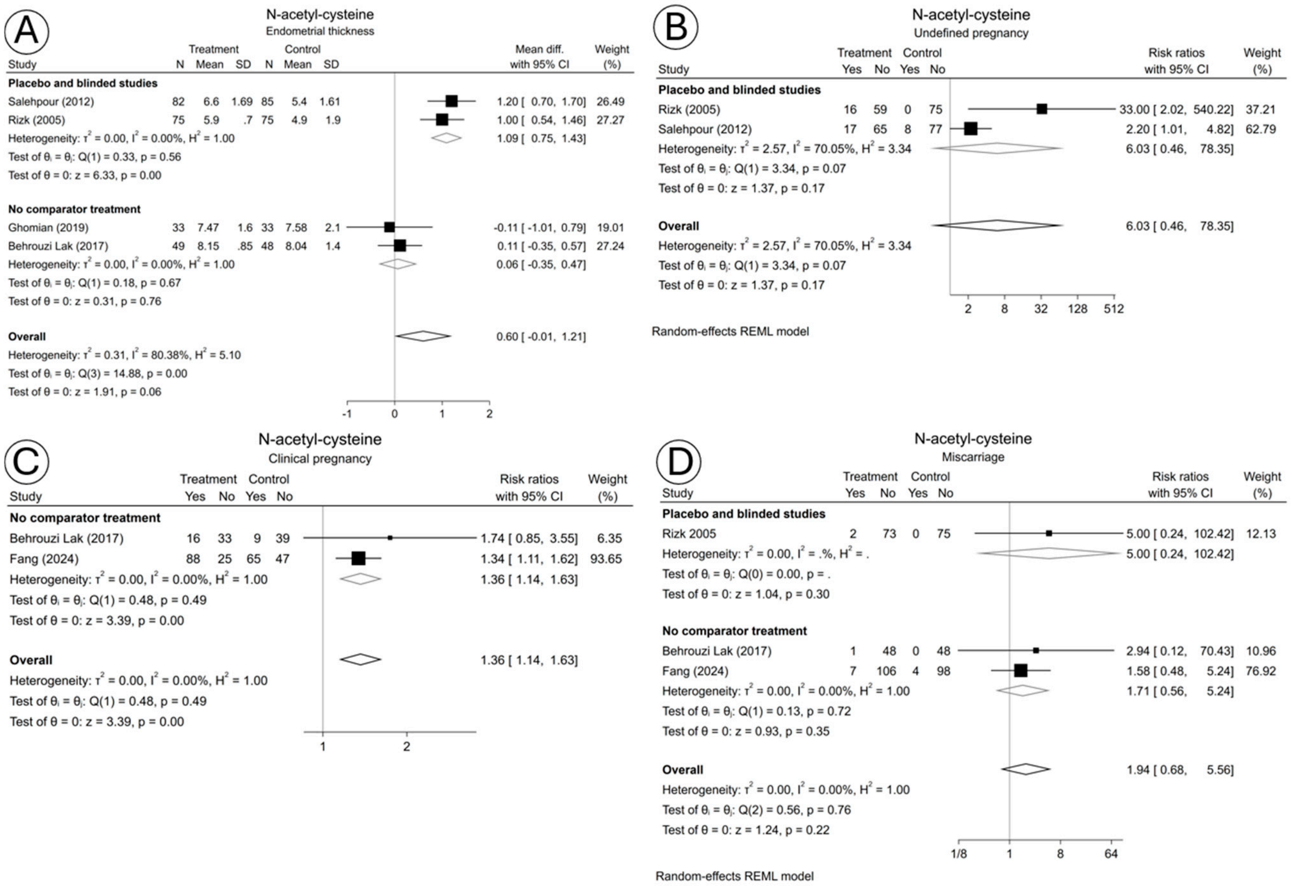

Figure S14. Forest plot of secondary analysis on the effect of N-acetyl-cysteine on (A) endometrial thickness, (B) undefined pregnancy, (C) clinical pregnancy, and (D) miscarriage.

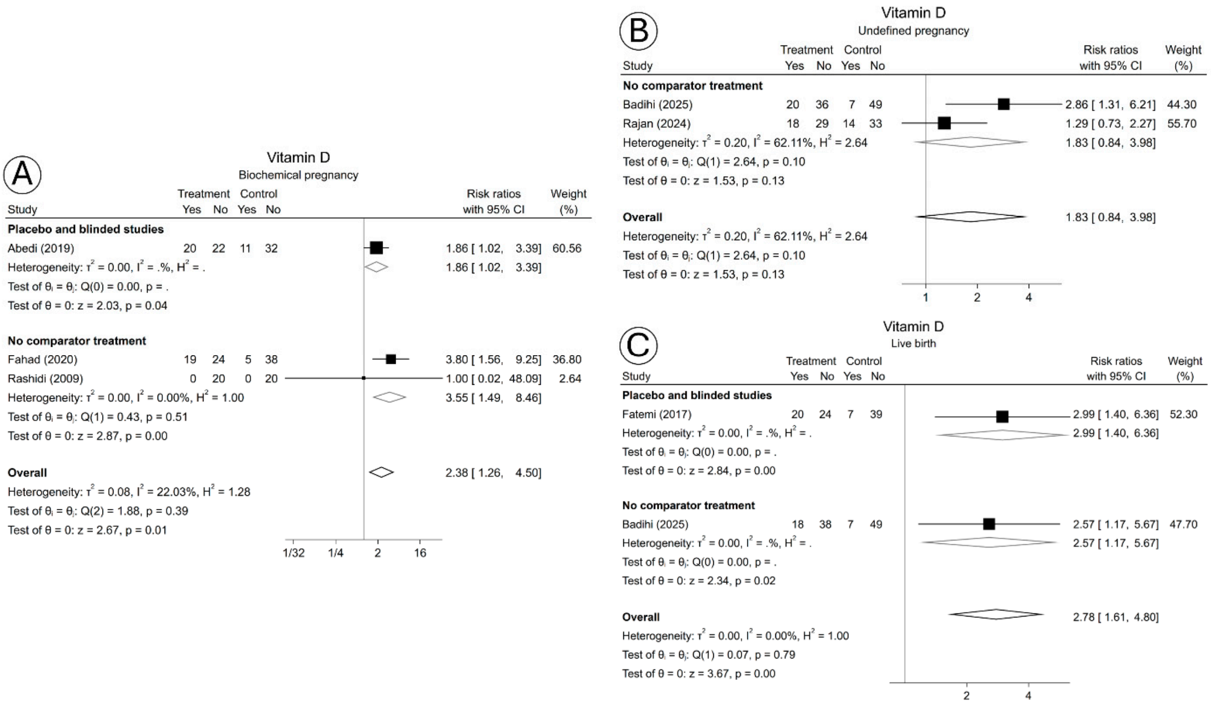

Figure S15. Forest plot of secondary analysis on the effect of vitamin D on (A) biochemical pregnancy, (B) undefined pregnancy, and (C) live birth.

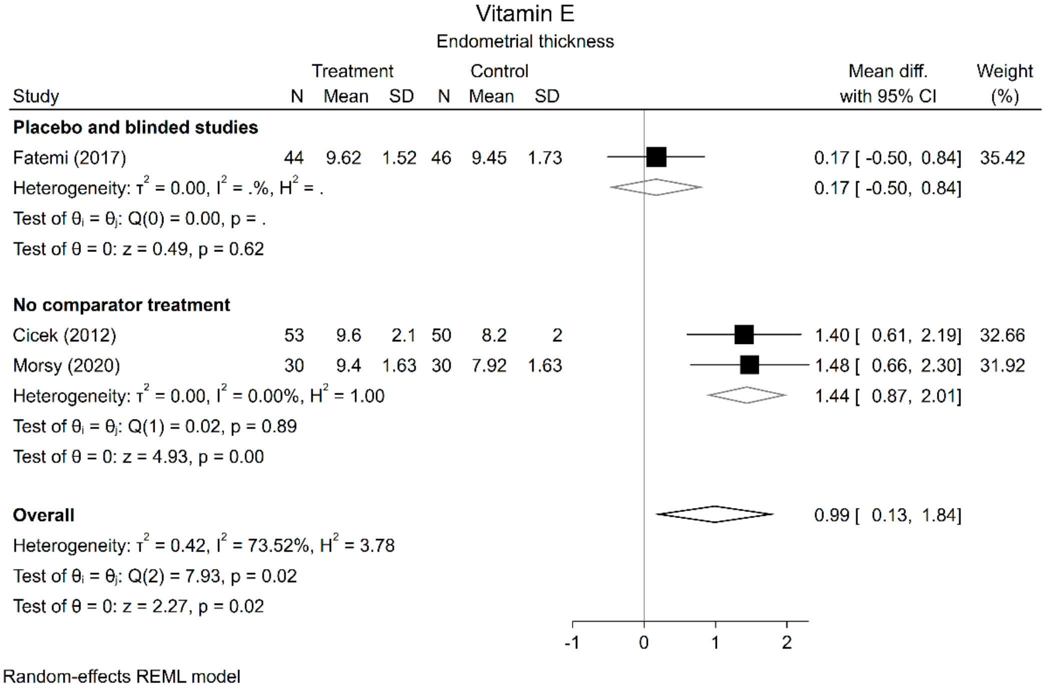

Figure S16. Forest plot of secondary analysis on the effect of Vitamin E on endometrial thickness.

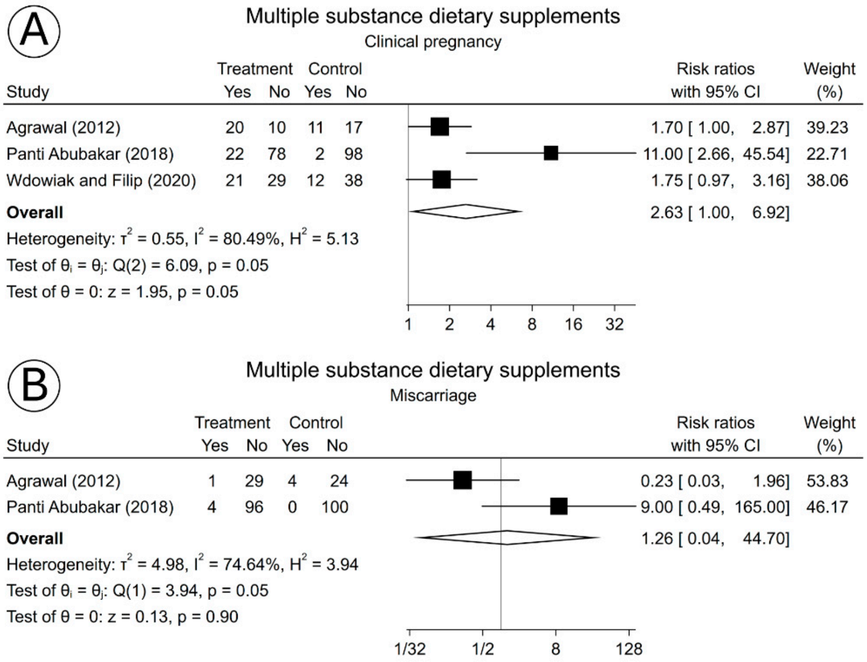

Figure S17. Forest plot of primary analysis on the effect of multiple substance dietary supplements on (A) clinical pregnancy and (B) miscarriage.

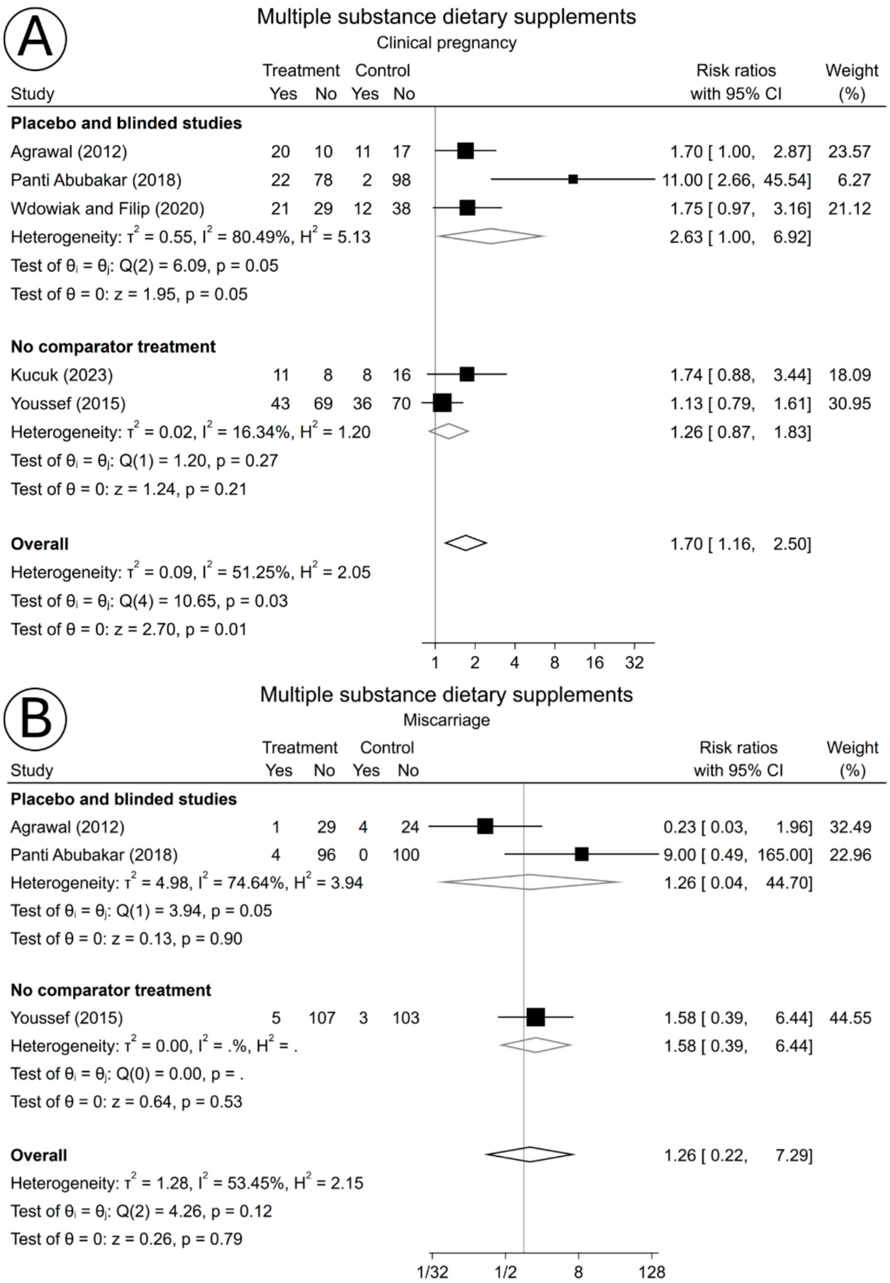

Figure S18. Forest plot of secondary analysis on the effect of multiple substance dietary supplements on (A) clinical pregnancy and (B) miscarriage.

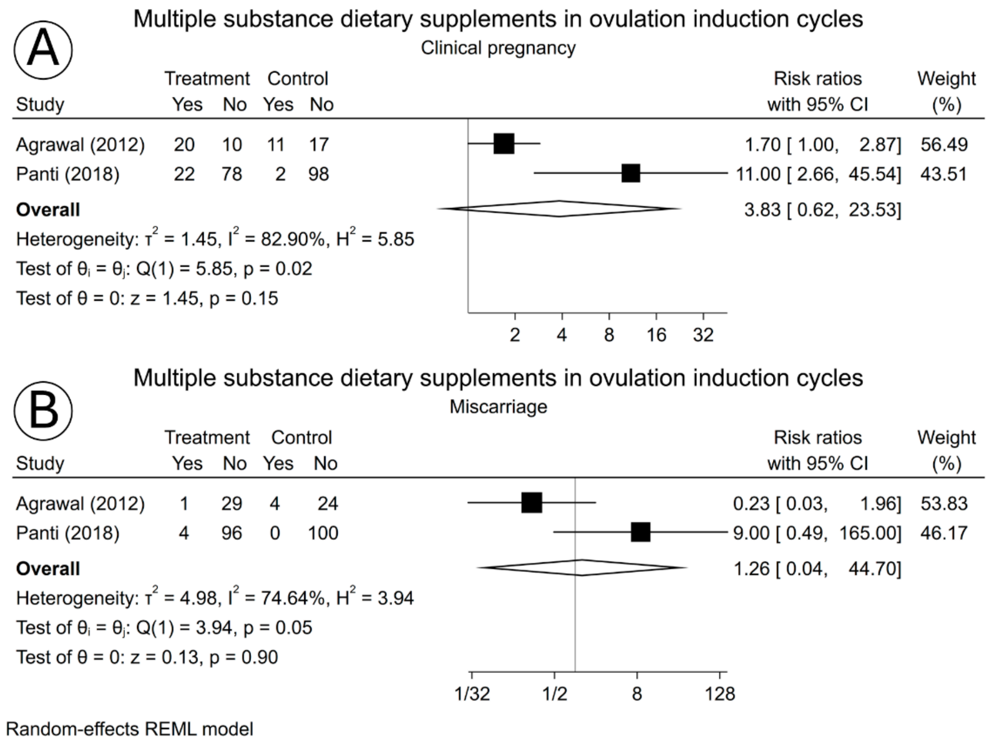

Figure S19. Forest plot of subgroup analysis on the effect of multiple substance dietary supplements on (A) clinical pregnancy and (B) miscarriage in in ovulation induction cycles.

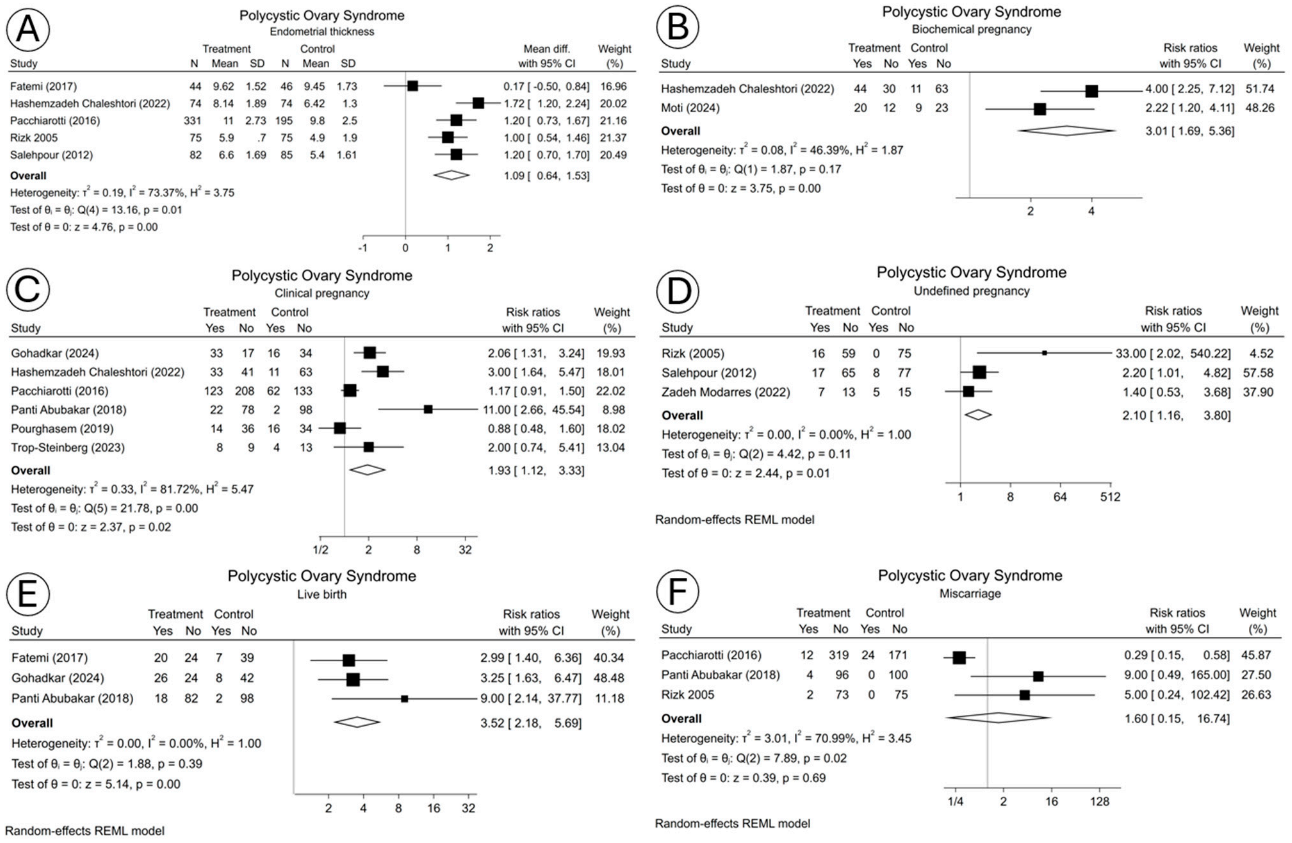

Figure S20. Forest plot on exploratory subgroup analysis on the effect of dietary supplements on (A) endometrial thickness, (B) biochemical pregnancy, (C) clinical pregnancy, (D) undefined pregnancy, (E) live birth, and (F) miscarriage in infertile women with polycystic ovary syndrome.

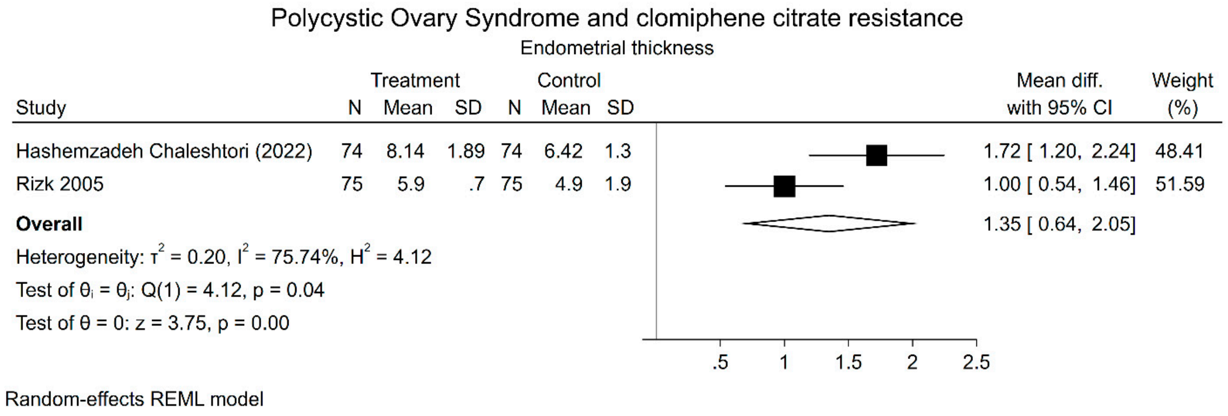

Figure S21. Forest plot on exploratory subgroup analysis on the effect of dietary supplements on endometrial thickness in infertile women with polycystic ovary syndrome and clomiphene citrate resistance.
